# Arl8b inactivates the Rab11a recycling pathway to promote LAMP1 sorting and lysosome biogenesis

**DOI:** 10.1083/jcb.202509040

**Published:** 2026-05-21

**Authors:** Priya Chouhan, Yogita Phogat, Kshitiz Walia, Saikat Debnath, Sandeep Choubey, Medha Gupta, Amit Tuli, Mahak Sharma

**Affiliations:** 1 Division of Cell Biology and Immunology, CSIR-Institute of Microbial Technology (IMTECH), Chandigarh, India; 2 Academy of Scientific and Innovative Research (AcSIR), Ghaziabad, India; 3 Department of Biological Sciences, Indian Institute of Science Education and Research (IISER), Mohali, India; 4 M. V. College, Buxar, India; 5 The Institute of Mathematical Sciences (IMSc), Chennai, India; 6 Homi Bhabha National Institute (HBNI), Mumbai, India

## Abstract

The small GTP-binding protein Arl8b is established as a regulator of lysosome positioning and fusion, yet its role in lysosome biogenesis remains unclear. Here, we investigate the role of Arl8b in the trafficking of newly synthesized LAMP1 to lysosomes using the Retention Using Selective Hook (RUSH) assay. We find that Arl8b localizes to post-endocytic LAMP1-containing vesicles prior to fusion with acidic lysosomes. Arl8b depletion leads to Rab11a-dependent recycling of LAMP1 to the plasma membrane, impairing its lysosomal delivery. Mechanistically, Arl8b recruits the Rab11a GAP, TBC1D9B, to LAMP1-positive membranes, and TBC1D9B depletion similarly disrupts LAMP1 sorting. Notably, TBC1D9B knockdown also impairs the retrieval of cation-independent mannose-6-phosphate receptor (CI-M6PR) from Rab11a- and Rab14-positive endosomes to the trans-Golgi network, impairing pro-cathepsin trafficking and cargo degradation. These findings reveal that Arl8b-mediated recruitment of Rab GAP TBC1D9B is crucial for inactivation of the Rab11a recycling pathway, leading to efficient sorting of lysosomal cargo to their functional location.

## Teaser

Lysosomal cargo sorting from the early endosomes should involve mechanisms that prevent their recycling to the plasma membrane. Here, Chouhan P and Phogat Y et al. report that the small GTP-binding protein Arl8b recruits the Rab11a GAP, TBC1D9B, to inactivate Rab11a-mediated recycling of newly synthesized LAMP1 and mediate its efficient sorting to lysosomes.

## Introduction

Lysosomes are a heterogeneous collection of membrane-bound compartments, canonically known as the recycling center of the cell for their role in cargo degradation and recycling of the building blocks for *de novo* macromolecular synthesis. Importantly, only a subpopulation of lysosomes are acidic and degradative, and this degradative potential is required for activation of the lysosomally localized master regulator kinase mTORC1, which in turn regulates lysosome biogenesis and acidification in response to nutrient availability (Condon and Sabatini, 2019; Johnson et al., 2016; Ratto et al., 2022; Sancak et al., 2010). Thus, lysosomes are now regarded as the control center for cellular metabolism. In addition to their role in cargo degradation, lysosomes also participate in other subcellular processes, such as maintaining the morphology and distribution of organelles, including ER and mitochondria, cholesterol homeostasis, plasma membrane repair, and cell migration (Ballabio and Bonifacino, 2020; Meng et al., 2020).

Lysosomes receive cargo for degradation via fusion with late endosomes, phagosomes, and autophagosomes, forming hybrid compartments, such as endolysosomes, where cargo degradation takes place (Bright et al., 2016; Lőrincz and Juhász, 2020; Nguyen and Yates, 2021). Here, we employ the term “endolysosomes” or “active lysosomes” to denote the acidic and degradative compartments. Intriguingly, lysosomes are reformed by the tubulation and fission of the hybrid endolysosomal, autolysosomal, and phagolysosomal compartments (Bright et al., 1997; Levin et al., 2016; Yang and Wang, 2021; Yu et al., 2010). Small GTPases, Rab7a, Rab2a, and Arf-like GTPase Arl8b, are the major regulators of lysosomal fusion, coordinating membrane tethering and fusion events (Guerra and Bucci, 2016; Khatter et al., 2015a; Lőrincz et al., 2017; Marwaha et al., 2017; Schleinitz et al., 2023). Arl8b regulates the tethering and fusion of late endosomes and autophagosomes with lysosomes through interaction with the Rab7 effector PLEKHM1 and Vps41, a subunit of the late endosomal/lysosomal multisubunit tethering factor HOPS complex, facilitating the assembly of fusion machinery (Khatter et al., 2015a; Marwaha et al., 2017). Rab2a interacts with Vps39, another subunit of the HOPS complex, and mediates fusion of late endosomes and autophagosomes with Arl8b-positive lysosomes (Lőrincz et al., 2017; Schleinitz et al., 2023). Apart from its role in membrane fusion, Arl8b also regulates the motor-dependent transport of lysosomes on microtubule tracks by recruiting effectors PLEKHM2/SKIP and RUFY3, which in turn recruit kinesin-1 and the JIP4–dynein–dynactin complex to mediate the anterograde and retrograde motility of lysosomes, respectively (Hofmann and Munro, 2006; Keren-Kaplan et al., 2022; Kumar et al., 2022; Rosa-Ferreira and Munro, 2011). Importantly, in addition to conventional degradative lysosomes, Arl8b is also localized on lysosome-related organelles such as lytic granules in natural killer cells and secretory lysosomes in osteoclasts (Tuli et al., 2013; Walia et al., 2026). Moreover, Arl8b regulates presynaptic vesicle biogenesis by regulating axonal co-transport of synaptic vesicles and active zone proteins in presynaptic lysosome-related vesicles in neurons (Klassen et al., 2010; Vukoja et al., 2018).

In a previous study, we identified a novel localization and function of Arl8b on Rab14-positive early/recycling endosomes (REs), wherein it interacts with the Rab14 effector, RUFY1, and is required for RUFY1 endosomal localization. RUFY proteins act as dynein adaptors; specifically, RUFY1 mediates dynein-dependent retrograde trafficking of cation-independent mannose-6-phosphate receptor (CI-M6PR) from Rab14-positive endosomes to the trans-Golgi network (TGN) (Rawat et al., 2023). Interestingly, RUFY1 also directly interacts with the AP-3 adaptor protein complex (Ivan et al., 2012). AP-3 is the sorting adaptor for lysosomal glycoproteins LAMP1, LAMP2, and CD63 that binds to the YXXΦ-type sorting motif in their cytoplasmic tails, thereby mediating their transport from early endosomes to lysosomes (Braulke and Bonifacino, 2009; Ma et al., 2021). Consequently, loss of AP-3 expression leads to missorting of these lysosomal cargoes toward the cell surface (Ivan et al., 2012; Peden et al., 2004).

Recent studies employing the Retention Using Selective Hook (RUSH) assay have clarified the biosynthetic transport route of lysosomal glycoproteins LAMP1 and LAMP2 to their steady-state lysosomal distribution (Chen et al., 2017; Ecard et al., 2024; Li et al., 2024; Pereira et al., 2023). These studies have shown that LAMP1 and CI-M6PR are segregated in the Golgi itself, and LAMP1/LAMP2 primarily follow an “indirect” route to reach lysosomes, as they exit the Golgi complex in tubular carriers that fuse with the plasma membrane, followed by AP-2–dependent endocytosis and delivery from early endosomes to lysosomes. LAMP1 and LAMP2 also traffic from the Golgi in “LAMP carriers,” which are uncoated vesicles that mediate direct delivery of LAMPs to late endosomes (Pols et al., 2013b). The “direct” pathway of LAMP1 sorting is regulated by the HOPS subunit Vps41, the vesicle-associated SNARE protein VAMP7, and Arl8b, which mediates the association of Vps41 with the TGN-derived LAMP carriers (Pols et al., 2013b; Sanzà et al., 2025).

The current study began with an unexpected observation that depletion or knockout (KO) of Arl8b resulted in increased LAMP1 levels at the cell surface. Based on our earlier observations that AP-3 binder RUFY1 is an Arl8b interactor, we hypothesized that Arl8b may regulate LAMP1 sorting from AP-3–positive early endosomes (Ivan et al., 2012; Rawat et al., 2023). Here, we report that Arl8b localizes to the newly synthesized post-endocytic vesicles containing LAMP1, and depletion of Arl8b led to a delay in LAMP1 sorting from AP-3–positive early endosomes to LysoTracker (LTR)-positive vesicles (hereafter referred to as active lysosomes). We found that newly synthesized LAMP1 was colocalized with Rab11a-positive REs upon Arl8b depletion, resulting in enhanced recycling of LAMP1 to the cell surface in a Rab11a-dependent manner. Intriguingly, we found that the Tre2-Bub2-Cdc16 (TBC) domain–containing protein TBC1D9B, a known Rab11a GTPase-activating protein (GAP), interacts with Arl8b (Gallo et al., 2014; Nishino et al., 2019). Arl8b recruits TBC1D9B on non-acidic LAMP1-positive vesicles, which may represent lysosomal cargo en route to fusion with active lysosomes. Depletion of TBC1D9B, similar to Arl8b, led to Rab11a-dependent missorting of LAMP1 to the cell surface. TBC1D9B depletion also led to a defect in retrieval of CI-M6PR from Rab11a- and Rab14-positive REs to the TGN, impairing delivery of pro-cathepsin D to lysosomes. Consistent with a defect in lysosomal composition, TBC1D9B depletion led to impaired degradation of endocytic and autophagic cargoes. Our study suggests that Arl8b, through its effector TBC1D9B, regulates the efficient sorting and delivery of LAMP1 and the endosome-to-TGN retrieval of CI-M6PR for the sorting of lysosomal hydrolases, thereby mediating lysosome biogenesis.

## Results

### Arl8b depletion leads to an increase in surface LAMP1, which is independent of active lysosome exocytosis

**Figure S1.**
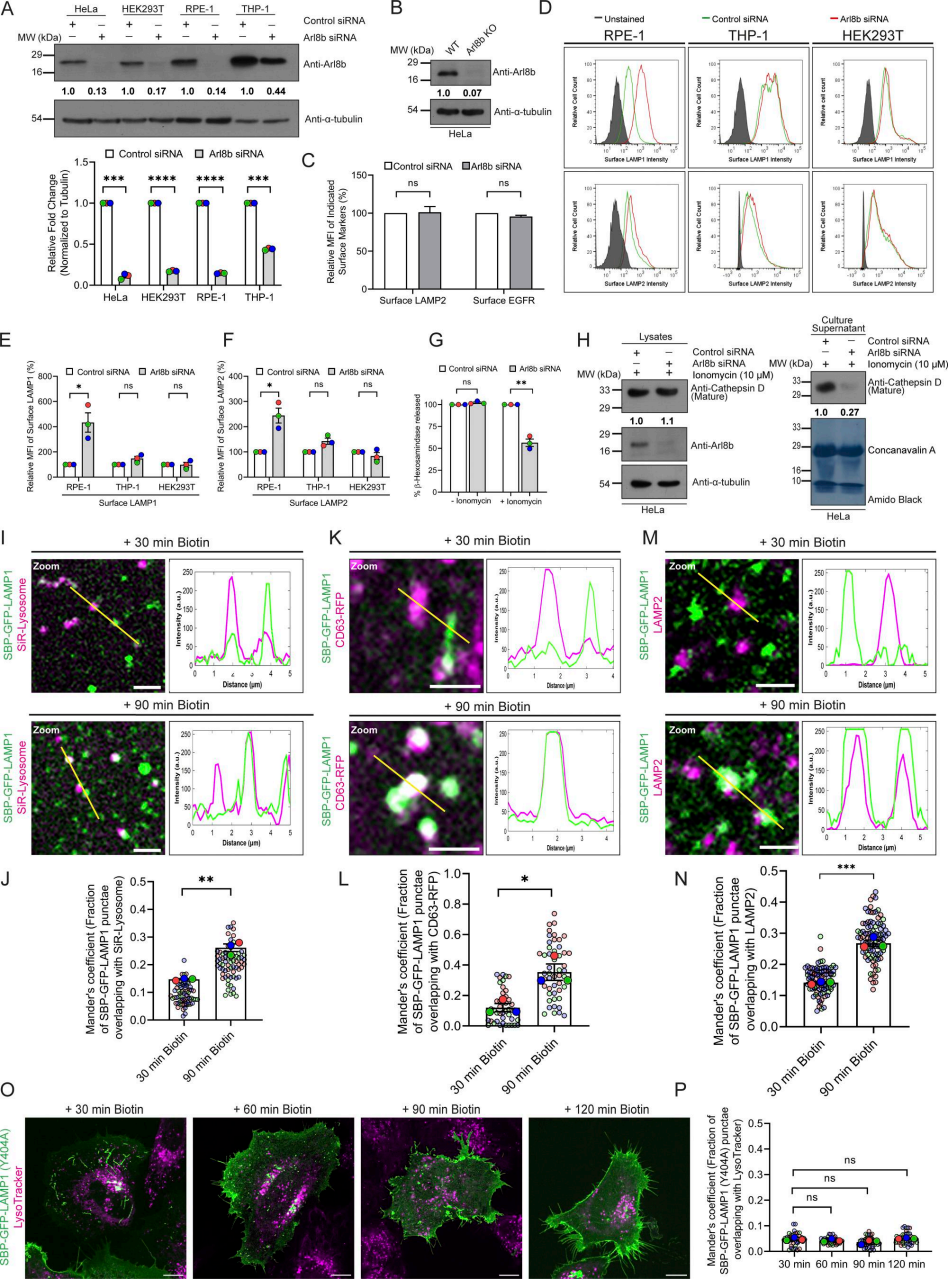
**Increase in surface LAMP1 levels upon Arl8b depletion is independent of active lysosome exocytosis. (A)** Lysates of HeLa, HEK293T, RPE-1, and THP-1 cells treated with control or Arl8b siRNA were IB with the indicated antibodies. The values indicate densitometric analysis of Arl8b levels normalized to α-tubulin. The bar graph represents the relative fold change in densitometric values of Arl8b normalized to α-tubulin. Statistical significance was calculated using the one-sample *t* test (*n* = 3; ***P = 0.0005 (HeLa); ***P = 0.0002 (THP-1); ****P < 0.0001). The values are represented as the mean ± SEM. **(B)** Wild-type or Arl8b KO HeLa cell lysates were IB for the indicated proteins. The values represent densitometric analysis of Arl8b levels normalized to α-tubulin. **(C)** Graph represents the MFI of surface LAMP2 and surface EGFR levels upon Arl8b siRNA treatment and normalized to control siRNA (*n* = 3). Statistical significance was calculated using the one-sample *t* test (n.s., non-significant). The values are represented as the mean ± SEM. **(D)** Representative histogram showing MFI of surface LAMP1 and LAMP2 in control or Arl8b siRNA-treated RPE-1, THP-1, and HEK293T cells as analyzed by flow cytometry. **(E and F)** Graphs represent the MFI of surface LAMP1 (E) (*n* = 3; each dot represents a single experiment, *P *=* 0.0488; n.s., non-significant) and LAMP2 (F) (*n* = 3; each dot represents a single experiment, *P *=* 0.0392; n.s., non-significant) upon Arl8b siRNA treatment and normalized to control siRNA. Statistical significance was calculated using the one-sample *t* test. The values are represented as the mean ± SEM. **(G)** Percentage of β-hexosaminidase release was quantified from the culture supernatant of HeLa cells treated with control or Arl8b siRNA upon ionomycin addition (*n* = 3; each dot represents a single experiment). Statistical significance was calculated using the one-sample *t* test (**P = 0.0092, n.s., non-significant). The values are represented as the mean ± SEM. **(H)** Cathepsin D levels in lysates and culture supernatants of HeLa cells treated with control or Arl8b siRNA upon ionomycin addition were measured by immunoblotting, and amido black staining was performed to visualize proteins. The values represent densitometric analysis of cathepsin D levels normalized to α-tubulin (lysate) or total protein (supernatant). **(I)** Representative confocal micrographs from live-cell imaging of HeLa cells expressing SBP-GFP-LAMP1 and lysosomes labeled with the SiR-Lysosome probe (magenta). Representative inset images at 30 and 90 min after biotin addition are shown. The line profiles indicate fluorescence intensity of SBP-GFP-LAMP1 (green) and SiR-Lysosome probe (magenta) along the yellow line. Scale bar: 2 µm. **(J)** Quantification of Manders’ colocalization coefficient of SBP-GFP-LAMP1 with the SiR-Lysosome probe at different time points is shown from three independent experiments (*n* = 3). Colors in the SuperPlots indicate individual experiments, with each dot representing a single cell. The mean value for each experiment is indicated by a larger dot. Statistical significance was calculated using unpaired Student’s *t* test (**P = 0.0012). The values are represented as the mean ± SEM. **(K)** Confocal micrographs from live-cell imaging of HeLa cells co-expressing SBP-GFP-LAMP1 and CD63-RFP at 30 and 90 min after biotin addition are shown. The line profiles indicate fluorescence intensity of SBP-GFP-LAMP1 (green) and CD63-RFP (magenta) along the yellow line. Scale bar: 2 µm. **(L)** Quantification of Manders’ colocalization coefficient of SBP-GFP-LAMP1 with CD63-RFP at different time points is shown from three independent experiments (*n* = 3). Colors in the SuperPlots indicate individual experiments, with each dot representing a single cell. The mean value for each experiment is indicated by a larger dot. Statistical significance was calculated using unpaired Student’s *t* test (*P = 0.0182). The values are represented as the mean ± SEM. **(M)** Confocal micrographs of HeLa cells expressing SBP-GFP-LAMP1 and immunostained for LAMP2 (magenta) at 30 and 90 min after biotin addition are shown. The line profiles indicate fluorescence intensity of SBP-GFP-LAMP1 (green) and LAMP2 (magenta) along the yellow line. Scale bar: 2 µm. **(N)** Quantification of Manders’ colocalization coefficient of SBP-GFP-LAMP1 with LAMP2 at different time points is shown from three independent experiments (*n* = 3). Colors in the SuperPlots indicate individual experiments, with each dot representing a single cell. The mean value for each experiment is indicated by a larger dot. Statistical significance was calculated using unpaired Student’s *t* test (***P = 0.0003). The values are represented as the mean ± SEM. **(O)** HeLa cells expressing SBP-GFP-LAMP1 (Y404A) are labeled with LTR (magenta). Confocal micrographs of live-cell imaging are shown at different time points after biotin addition. Scale bar: 10 µm. **(P)** Graph indicates Manders’ colocalization coefficient quantification of SBP-GFP-LAMP1 (Y404A) with the LTR from three independent experiments (*n* = 3). Colors in the SuperPlots indicate individual experiments, with each dot representing a single cell. The mean value for each experiment is indicated by a larger dot. Statistical significance was calculated by one-way ANOVA with Dunnett’s multiple comparisons test (n.s., non-significant). The values are represented as the mean ± SEM. Source data are available for this figure: SourceData FS1.

**Figure 1.**
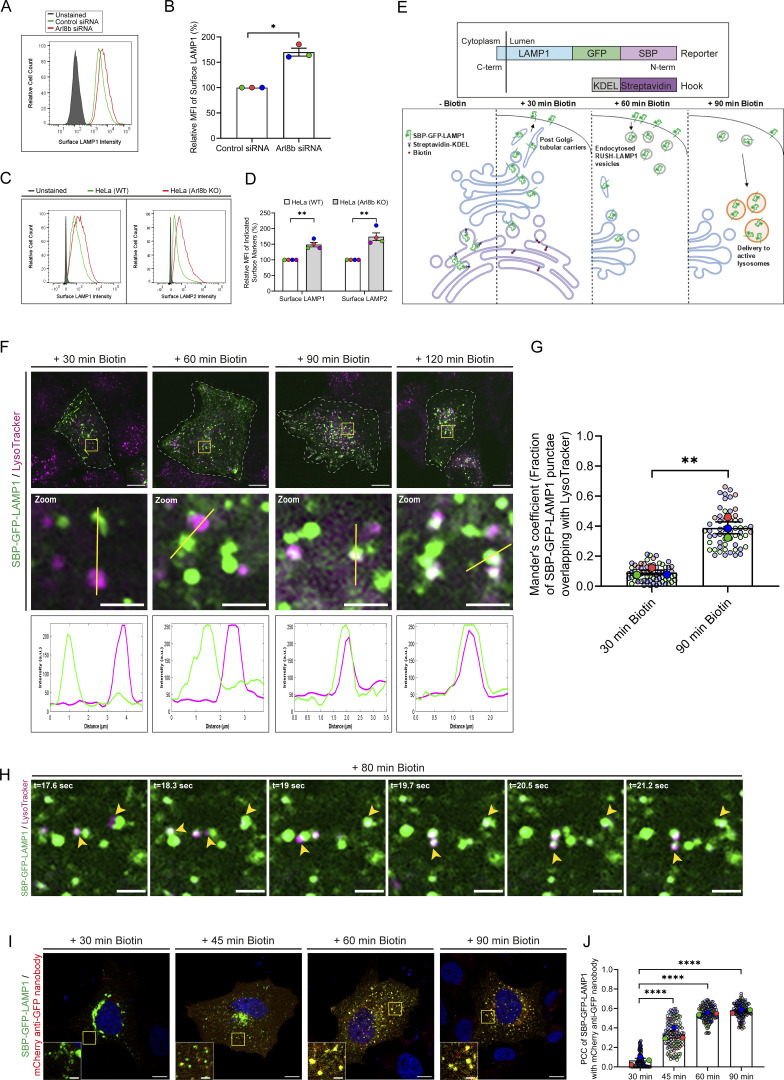
**Arl8b depletion leads to an increase in surface LAMP1 levels and characterization of newly synthesized LAMP1 trafficking to active lysosomes. (A)** Representative histogram showing MFI of surface LAMP1 in control or Arl8b siRNA-treated HeLa cells as analyzed by flow cytometry. **(B)** Bar graph represents the percent MFI of surface LAMP1 in HeLa cells treated with indicated siRNAs normalized to control siRNA from three independent experiments (*n* = 3). Statistical significance was calculated using one-sample *t* test (*P = 0.0122). The values are represented as the mean ± SEM. **(C)** Representative histogram of wild-type (WT) or Arl8b KO HeLa cells showing MFI of surface LAMP1 and LAMP2 as analyzed by flow cytometry. **(D)** Bar graph represents the percent MFI of surface LAMP1 and LAMP2 in Arl8b KO cells normalized to WT cells from four independent experiments (*n* = 4). Statistical significance was calculated using one-sample *t* test (**P = 0.0064 [LAMP1]; **P *=* 0.0095 [LAMP2]). The values are represented as the mean ± SEM. **(E)** Schematic depiction of the RUSH assay used to investigate the trafficking of newly synthesized LAMP1. ER hook streptavidin-KDEL, along with a reporter (LAMP1) fused with SBP-GFP, is expressed in HeLa cells. The KDEL hook enables retention of the reporter in the ER through interaction between streptavidin and SBP. Biotin addition allows the reporter to be released and subsequently trafficked via the secretory pathway. The schematic is created using BioRender. **(F)** Representative confocal micrographs from a live-cell imaging experiment of HeLa cells expressing the RUSH reporter (SBP-GFP-LAMP1) and labeled with LTR. The line profiles indicate fluorescence intensity along the yellow lines for both channels: SBP-GFP-LAMP1 (green) and LTR (magenta). Scale bars: 10 μm (main); 2 μm (inset). **(G)** Quantification of Manders’ colocalization coefficient of SBP-GFP-LAMP1 with LTR is shown from three independent experiments (*n* = 3). Colors in the SuperPlots indicate individual experiments, with each dot representing a single cell. The mean value for each experiment is indicated by a larger dot. Statistical significance was calculated using unpaired Student’s *t* test (**P = 0.0022). The values are represented as the mean ± SEM. **(H)** Representative live-cell time-lapse images of HeLa cells expressing SBP-GFP LAMP1 (green) and LTR (magenta) after 80 min of biotin addition. The yellow arrowheads indicate SBP-GFP-LAMP1– and LTR-positive vesicles undergoing kiss-and-run events. Scale bar: 2 µm. **(I)** Representative confocal micrographs of HeLa cells expressing SBP-GFP-LAMP1. Cells were incubated with mCherry-tagged anti-GFP nanobody at the time of biotin addition, followed by fixation at the indicated time points. Scale bars: 10 μm (main); 2 μm (inset). **(J)** PCC of SBP-GFP-LAMP1 with mCherry-tagged anti-GFP nanobody at different time points of biotin addition from three independent experiments (*n* = 3). Colors in the SuperPlots indicate individual experiments, with each dot representing a single cell. The mean value for each experiment is indicated by a larger dot. Statistical significance was calculated by one-way ANOVA with Dunnett’s multiple comparisons test (****P < 0.0001). The values are represented as the mean ± SEM. MFI, mean fluorescence intensity; PCC, pearson's correlation coefficient.

Arl8b is a well-established key regulator of kinesin and dynein-dependent lysosome motility and lysosome fusion with late endosomes and autophagosomes (Bagshaw et al., 2006; Garg et al., 2011a; Hofmann and Munro, 2006; Keren-Kaplan et al., 2022; Khatter et al., 2015a; Kumar et al., 2022; Marwaha et al., 2017; Rosa-Ferreira and Munro, 2011; Sindhwani et al., 2017). However, its role in lysosome biogenesis has been less explored. We made a surprising observation that Arl8b depletion or KO in HeLa cells led to a significant increase in surface levels of lysosomal glycoprotein LAMP1, as compared to the control (Fig. S1, A and B; and Fig. 1, A–D). Notably, surface levels of LAMP2 were similar to control upon siRNA-mediated silencing of Arl8b, but a significant increase was observed in Arl8b KO cells (Fig. S1 C; and Fig. 1, C and D). As a control, we analyzed surface levels of another single-pass transmembrane receptor, epidermal growth factor receptor (EGFR), which was not altered upon Arl8b knockdown (Fig. S1 C). A recent study has shown that lysosome number, area, and proteolytic capacity are heterogeneous across commonly employed epithelial and macrophage cell lines (Bussi and Gutierrez, 2024). Taking this lysosomal heterogeneity into account, we assessed the surface LAMP1 and LAMP2 levels in RPE-1, THP-1, and HEK293T cell lines that were also employed in this previous study (Bussi and Gutierrez, 2024). We found that Arl8b depletion led to a significant increase in surface LAMP1 and LAMP2 levels in RPE-1 cells, whereas no change in LAMP1 or LAMP2 levels was observed in HEK293T cells (Fig. S1, A and D–F). In THP-1 macrophages, we found a modest but not significant increase in surface LAMP1 and LAMP2 levels; however, the knockdown efficiency in these cells was also suboptimal as compared to other cell lines (Fig. S1, A and D–F). It is plausible that differential expression of Arl8 paralogs may contribute to the observed heterogeneity in surface LAMP1 levels across these different cell lines.

An increase in surface LAMP1 could imply enhanced exocytosis of active lysosomes in Arl8b-depleted cells. However, no change was observed in β-hexosaminidase release upon Arl8b knockdown; rather, consistent with a previous study, we found that ionomycin-induced lysosomal exocytosis (assessed by measuring β-hexosaminidase and mature cathepsin D release) was reduced upon Arl8b depletion (Michelet et al., 2018) (Fig. S1, G and H). These observations suggest a role of Arl8b in regulating LAMP1 trafficking, which might explain LAMP1 missorting to the cell surface upon its depletion.

### Arl8b localizes to newly synthesized LAMP1-containing endocytic vesicles prior to their fusion with active lysosomes

To test whether Arl8b regulates trafficking of LAMP1 as it reaches its functional location, we utilized the RUSH system. To this end, HeLa cells were transfected with a LAMP1 construct with an N-terminal streptavidin-binding protein (SBP) tag followed by a GFP tag (for simplicity, hereafter referred to as “RUSH-LAMP1”) and co-expressing streptavidin fused to the ER retention signal sequence, KDEL (hook). Following biotin addition, RUSH-LAMP1 will be released from the hook, allowing its trafficking over time to active lysosomes (Fig. 1 E). In line with previous studies, RUSH-LAMP1 was observed to traffic from the Golgi in tubular carriers within 30 min of biotin addition, and colocalization with acidic (LTR-positive) and active cathepsin-containing (SiR-Lysosome–positive) lysosomes was observed by 90–120 min of biotin treatment (Fig. 1, F and G; Fig. S1, I and J; and Video 1) (Chen et al., 2017; Ecard et al., 2024; Li et al., 2024; Pereira et al., 2023; Robinson et al., 2024). We observed multiple kiss-and-run events between RUSH-LAMP1 vesicles and active (i.e., LTR-positive) lysosomes near 90 min of biotin addition, suggesting that LAMP1 is delivered to its functional location via tethering and fusion events (Fig. 1 H and Video 2). The delivery of newly synthesized RUSH-LAMP1 to its lysosomal location was also validated by colocalization with CD63-RFP and endogenous LAMP2 (Fig. S1, K–N).

**Video 1.** **Time-lapse imaging of HeLa cells expressing SBP-GFP-LAMP1 and incubated with LTR to label active lysosomes.** The cells were incubated with biotin, and the route of SBP-GFP-LAMP1 trafficking was followed from 40 to 91 min after biotin addition. The video is captured at 2.77 frames/sec with a 3-min time interval between the frames. The movie is shown at 1 frame/sec, and the total number of frames displayed is 18.

**Video 2.** **Time-lapse imaging of HeLa cells expressing SBP-GFP-LAMP1 depicting fusion with active lysosomes.** The cells were incubated with biotin, and the route of SBP-GFP-LAMP1 trafficking was followed after 80 min of biotin addition. The video is captured at 2.54 frames/sec with no time interval between the frames. The movies (whole cell and marked inset) are shown at 8 frames/sec, and the total number of frames displayed is 50 for each. The yellow arrows in the inset movie depict kiss-and-run events between RUSH-LAMP1–positive vesicles with LTR-labeled active lysosomes.

Consistent with previous studies, we observed that RUSH-LAMP1 traffics via the plasma membrane (indirect pathway) for its delivery to active lysosomes, as the majority of RUSH-LAMP1 vesicles were bound to the mCherry-anti-GFP nanobody provided extracellularly to monitor its endocytosis (Fig. 1, I and J) (Chen et al., 2017; Ecard et al., 2024; Pereira et al., 2023). To further validate that newly synthesized LAMP1 traffics via the indirect pathway, we analyzed trafficking of an endocytosis-defective form of RUSH-LAMP1, i.e., LAMP1 Y404A (with a mutation in the “YQTI” AP-2–binding motif) (Traub and Bonifacino, 2013), to active lysosomes. In line with previous reports, we also observed RUSH-LAMP1 (Y404A) mutant trafficking in tubular carriers from the Golgi to the cell surface; however, unlike the WT protein, the LAMP1 (Y404A) mutant showed minimal colocalization with active lysosomes (Fig. S1, O–P) (Chen et al., 2017; Ecard et al., 2024; Pereira et al., 2023). This suggests that the majority of the newly synthesized LAMP1 is delivered to the active lysosomes after endocytosis from the cell surface.

**Figure S2.**
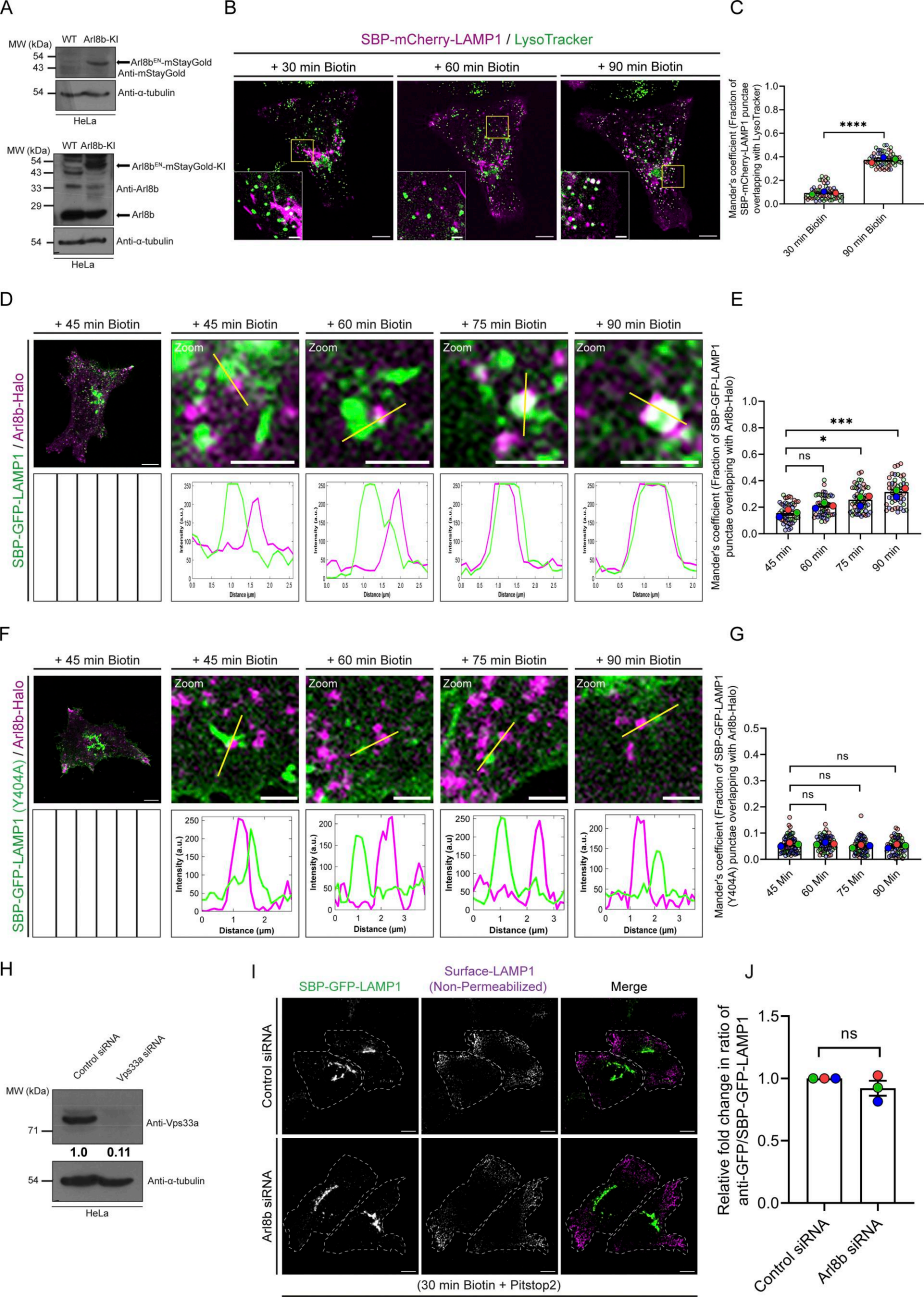
**Arl8b localizes to the newly synthesized post-endocytic LAMP1 vesicles. (A)** Lysates of WT and Arl8b-KI HeLa cells were IB with indicated antibodies. **(B)** Representative confocal micrographs from a live-cell imaging experiment of HeLa cells expressing the RUSH reporter (SBP-mCherry-LAMP1) and labeled with LTR. Scale bars: 10 μm (main); 2 μm (inset). **(C)** Quantification of Manders’ colocalization coefficient of SBP-mCherry-LAMP1 with LTR is shown from three independent experiments (*n* = 3). Colors in the SuperPlots indicate individual experiments, with each dot representing a single cell. The mean value for each experiment is indicated by a larger dot. Statistical significance was calculated using unpaired Student’s *t* test (****P < 0.0001). The values are represented as the mean ± SEM. **(D)** Representative images of HeLa cells co-expressing SBP-GFP-LAMP1 (green) and Arl8b-Halo (magenta), followed by live-cell imaging. Cells were incubated with biotin, and image insets for indicated time points are shown. The box with vertical lines represents blank space. Scale bars: 10 μm (main); 2 μm (inset). **(E)** Quantification of Manders’ colocalization coefficient of SBP-GFP-LAMP1 with Arl8b-Halo is shown from three independent experiments (*n* = 3). Colors in the SuperPlots indicate individual experiments, with each dot representing a single cell. The mean value for each experiment is indicated by a larger dot. Statistical significance was calculated by one-way ANOVA with Dunnett’s multiple comparisons test (*P = 0.0114; ***P = 0.0007; n.s., non-significant). The values are represented as the mean ± SEM. **(F)** Representative images of HeLa cells co-expressing SBP-GFP-LAMP1 (Y404A) (green) and Arl8b-Halo (magenta), followed by live-cell imaging. Cells were incubated with biotin, and image insets for indicated time points are shown. The black box with vertical lines represents blank space. Scale bars: 10 μm (main); 2 μm (inset). **(G)** Quantification of Manders’ colocalization coefficient of SBP-GFP-LAMP1 (Y404A) with Arl8b-Halo at the indicated time points is shown from three independent experiments (*n* = 3). Colors in the SuperPlots indicate individual experiments, with each dot representing a single cell. The mean value for each experiment is indicated by a larger dot. Statistical significance was calculated by one-way ANOVA with Dunnett’s multiple comparisons test (n.s., non-significant). The values are represented as the mean ± SEM. **(H)** HeLa cell lysates treated with control or Vps33a siRNAs were IB for the indicated proteins. **(I)** HeLa cells treated with the control or Arl8b siRNA and expressing SBP-GFP-LAMP1 were imaged after 30 min of biotin addition along with pitstop2 (30 µM). The trafficking of newly synthesized RUSH-LAMP1 from the TGN to the plasma membrane was visualized by surface staining with an anti-GFP antibody, followed by labeling with Alexa Fluor 568–conjugated secondary antibody. Scale bar: 10 µm. **(J)** Graph represents the relative ratio of surface signal (measured by an anti-GFP antibody) to total GFP signal of SBP-GFP-LAMP1 in HeLa cells treated with indicated siRNAs (*n* = 3; each dot represents a single experiment). Statistical significance was calculated using the one-sample *t* test (n.s., non-significant). The values are represented as the mean ± SEM. Source data are available for this figure: SourceData FS2.

**Figure 2.**
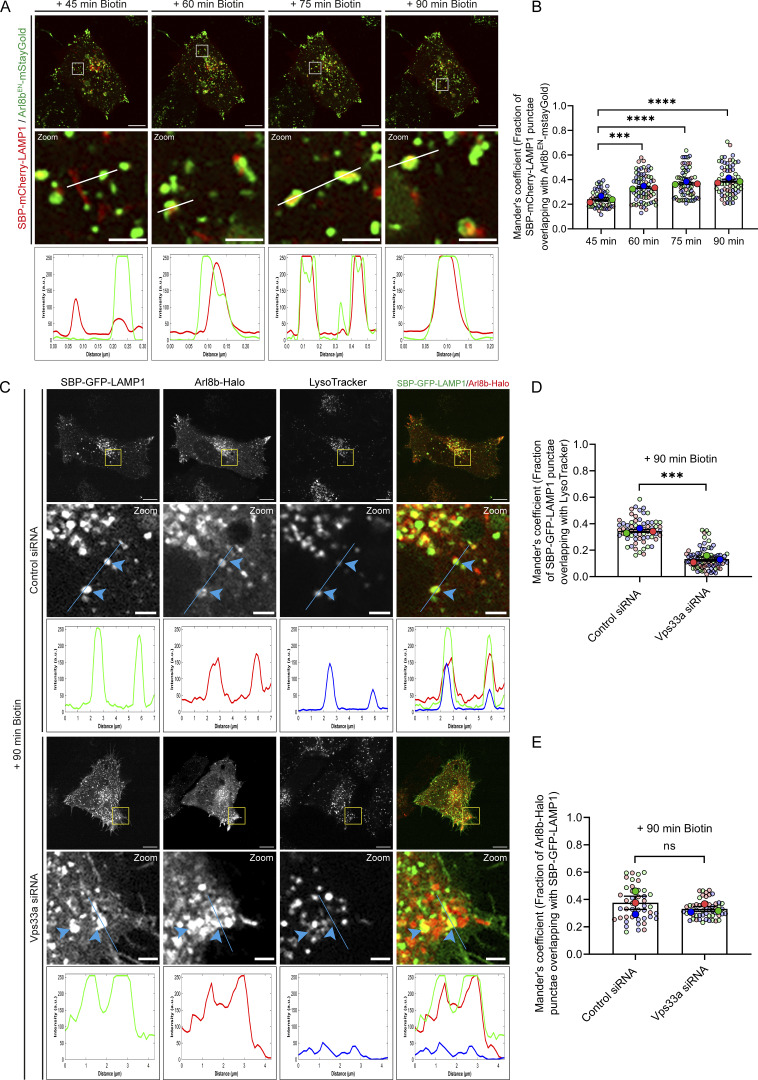
**Arl8b localizes to the newly synthesized post-endocytic RUSH-LAMP1 vesicles prior to fusion with active lysosomes. (A)** Representative confocal images of Arl8b^EN^-mStayGold KI HeLa cells expressing SBP-mCherry-LAMP1. Live-cell imaging was performed after biotin addition, and representative images for indicated time points are shown. Images are maximum-intensity projections of z-stack micrographs. The line profiles indicate fluorescence intensity along the white lines for both channels: SBP-mCherry-LAMP1 (red) and Arl8b^EN^-mStayGold (green). Scale bars: 10 μm (main); 2 μm (inset). **(B)** Quantification of Manders’ colocalization coefficient of SBP-mCherry-LAMP1 with Arl8b^EN^-mStayGold at the indicated time points is shown from three independent experiments (*n* = 3). Colors in the SuperPlots indicate individual experiments, with each dot representing a single cell. The mean value for each experiment is indicated by a larger dot. Statistical significance was calculated by one-way ANOVA with Dunnett’s multiple comparisons test (***P = 0.0006; ****P < 0.0001). The values are represented as the mean ± SEM. **(C)** Representative confocal micrographs of HeLa cells treated with control or Vps33a siRNA, followed by co-expression of SBP-GFP-LAMP1 (green) and Arl8b-Halo (red). Live-cell imaging was performed after cells were incubated with LTR (blue) dye to label acidic compartments, followed by biotin addition. Blue arrowheads indicate SBP-GFP-LAMP1– and Arl8b-Halo–positive vesicles. The line profiles indicate fluorescence intensity along the blue lines for all channels: SBP-GFP-LAMP1 (green), Arl8b-Halo (red), and LTR (blue). Scale bars: 10 μm (main); 2 μm (inset). **(D)** Quantification of Manders’ colocalization coefficient of SBP-GFP-LAMP1 with LTR in HeLa cells treated with the indicated siRNAs is shown from three independent experiments (*n* = 3). Colors in the SuperPlots indicate individual experiments, with each dot representing a single cell. The mean value for each experiment is indicated by a larger dot. Statistical significance was calculated using unpaired Student’s *t* test (***P = 0.0003). The values are represented as the mean ± SEM. **(E)** Quantification of Manders’ colocalization coefficient for SBP-GFP-LAMP1 with Arl8b-Halo in HeLa cells treated with the control or Vps33a siRNA is shown from three independent experiments (*n* = 3). Colors in the SuperPlots indicate individual experiments, with each dot representing a single cell. The mean value for each experiment is indicated by a larger dot. Statistical significance was calculated using unpaired Student’s *t* test (n.s., non-significant). The values are represented as the mean ± SEM.

Although Arl8b is known to localize to lysosomes, whether it also localizes to lysosomal cargo-containing secretory or endocytic vesicles is not known. To analyze whether endogenous Arl8b localizes on lysosomal cargo-containing vesicles, we visualized SBP-mCherry-LAMP1 in Arl8b-mStayGold knock-in (KI) HeLa cells (Arl8b^EN^-mStayGold). As expected, the Arl8b-mStayGold fusion protein was only detected in the KI cells and not the parental HeLa cells (Fig. S2 A). As these cells were a heterogeneous population and not clonally selected (to avoid any selection artifacts), the untagged endogenous Arl8b expression was also observed in both parental and the Arl8b^EN^-mStayGold KI cells (Fig. S2 A). The kinetics of RUSH-LAMP1 trafficking to active lysosomes was similar when mCherry was used as a reporter instead of GFP (Fig. S2, B and C). We observed that Arl8b^EN^-mStayGold did not colocalize with the post-Golgi tubular RUSH-LAMP1 carriers at 45 min after biotin treatment (Fig. 2, A and B). Similar results were observed with the overexpressed Halo-tagged Arl8b, which also showed minimal overlap with the post-Golgi tubular RUSH-LAMP1 carriers (Fig. S2, D–E). We also observed minimal or no colocalization of Arl8b with the endocytosis-defective RUSH-LAMP1 (Y404A) mutant that continues to traffic via post-Golgi carriers to the cell surface, suggesting that Arl8b does not localize to the post-Golgi tubular RUSH-LAMP1 carriers (Fig. S2, F and G).

Arl8b localization on the RUSH-LAMP1–positive vesicles was observed with increasing duration of biotin treatment, specifically from 45 to 60 min, and a further increase in colocalization was observed at 75 and 90 min of biotin addition (Fig. 2, A and B). Similarly, transiently expressed Halo-tagged Arl8b was recruited to the RUSH-LAMP1–positive vesicles at 75–90 min of biotin treatment (Fig. S2, D and E; and Video 3). These observations suggest that Arl8b is recruited on the post-endocytic RUSH-LAMP1 vesicles prior to their acquisition of an active lysosome identity. Indeed, a recent study has shown Arl8b localization on RUSH-LAMP1 vesicles between 20 min and 1 h of biotin treatment, which is before LAMP1 delivery to the pre-existing lysosomes (Li et al., 2024). It is relevant to note here that besides its lysosomal localization, Arl8b also localizes to a subset of early/REs that are positive for Rab14 and EEA1 and where it interacts with the Rab14 effector, RUFY1 (Rawat et al., 2023). Mass spectrometry–based high-throughput analysis has also identified Arl8b and its interaction partners, RUFY proteins, and the BLOC-one–related complex (BORC) in EEA1-positive membrane fractions, providing additional evidence for Arl8b localization on early endosomes (Park et al., 2022).

**Video 3.** **Time-lapse imaging of HeLa cells co-expressing SBP-GFP-LAMP1 and Arl8b-Halo.** The cells were incubated with biotin, and the route of SBP-GFP-LAMP1 trafficking was followed from 40 to 103 min after biotin addition. The video is captured at 2.22 frame/sec with a 3-min time interval between the frames. The movie is shown at 2 frame/sec, and the total number of frames displayed is 22.

To better visualize Arl8b recruitment on the newly synthesized LAMP1-containing endocytic vesicles before their fusion with active lysosomes, we employed a strategy to inhibit fusion of RUSH-LAMP1 vesicles with LTR-labeled active lysosomes. Because of its established role as a lysosomal tether and known interaction with Arl8b, we hypothesized that the HOPS complex regulates tethering and fusion of lysosomal cargo vesicles with the existing endolysosomes (Khatter et al., 2015a; Pols et al., 2013a; Ungermann and Moeller, 2025). Indeed, we found a significant decrease in RUSH-LAMP1 delivery to active lysosomes in cells depleted of the HOPS subunit Vps33a at 90 min of biotin addition (Fig. S2 H; and Fig. 2, C and D). Importantly, Arl8b colocalization with RUSH-LAMP1 was similar in both control and Vps33a-depleted cells, suggesting that Arl8b is recruited to the RUSH-LAMP1–containing endocytic vesicles prior to their fusion with active lysosomes (Fig. 2, C and E).

### Arl8b depletion results in the recycling of the newly synthesized LAMP1 to the cell surface

We next investigated whether Arl8b regulates the delivery of RUSH-LAMP1 to active lysosomes. To this end, we first analyzed RUSH-LAMP1 trafficking from the TGN to the plasma membrane upon Arl8b depletion. As LAMP1 is rapidly endocytosed from the cell surface in a clathrin-dependent manner, we treated cells with the chemical inhibitor pitstop2 (to block its endocytosis) and measured surface RUSH-LAMP1 at 30 min after biotin addition (Fig. S2, I and J) (Janvier and Bonifacino, 2005). Consistent with our prior observations that Arl8b does not localize to the Golgi-derived tubular LAMP1 carriers, we observed similar levels of RUSH-LAMP1 at the cell surface in control and Arl8b knockdown, suggesting that Arl8b does not regulate LAMP1 trafficking from the TGN to the cell surface (Fig. S2, I and J).

**Figure 3.**
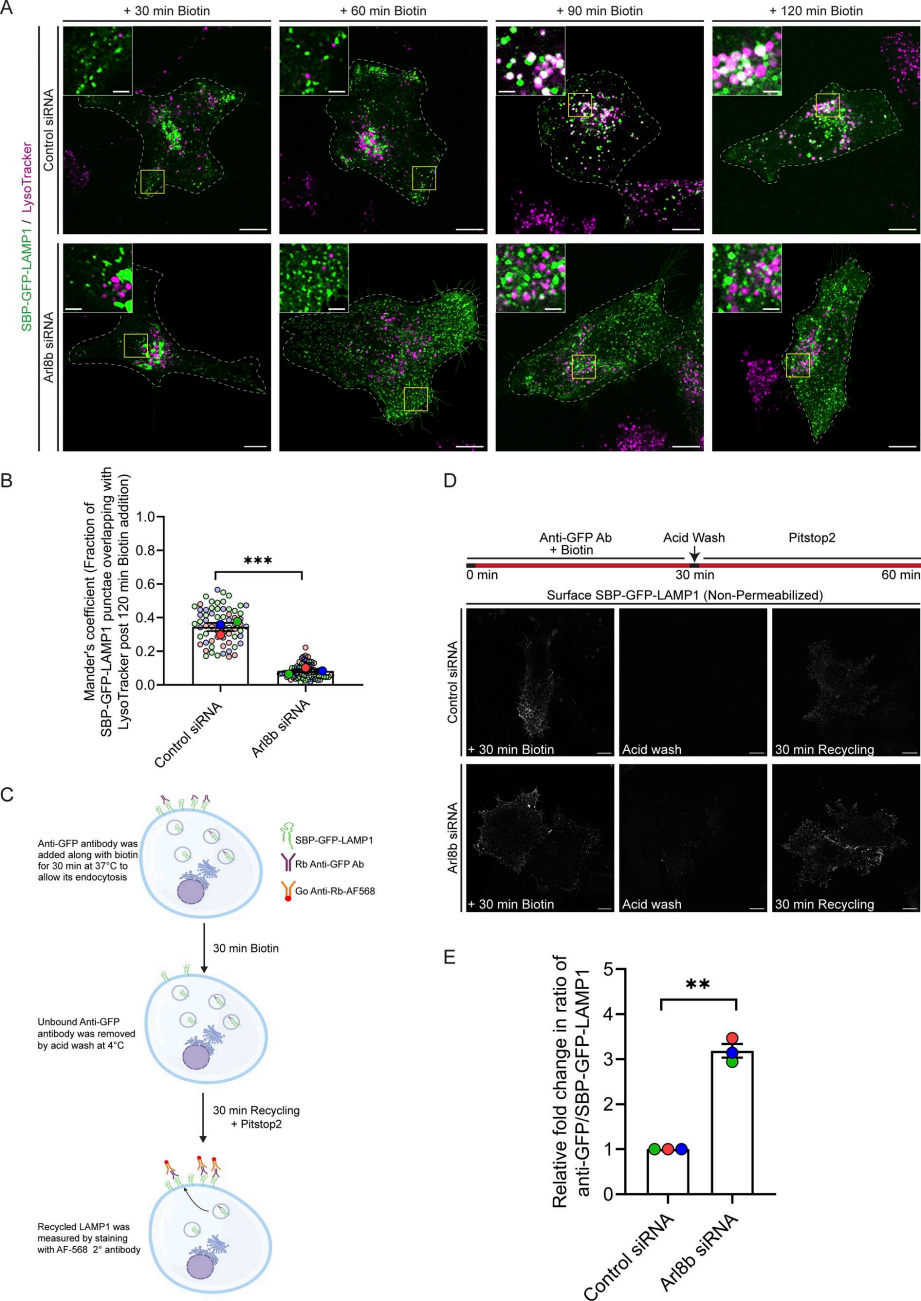
**Arl8b depletion enhances recycling of the newly synthesized LAMP1 to the plasma membrane, impairing its lysosomal delivery. (A)** Representative confocal micrographs of HeLa cells treated with control or Arl8b siRNA, transfected with SBP-GFP-LAMP1, followed by labeling with LTR (magenta). Images are taken at indicated time points after biotin addition. Scale bars: 10 µm (main); 2 μm (inset). **(B)** Quantification of Manders’ colocalization coefficient of SBP-GFP-LAMP1 with LTR in HeLa cells treated with indicated siRNAs is shown from three independent experiments (*n* = 3). Colors in the SuperPlots indicate individual experiments, with each dot representing a single cell. The mean value for each experiment is indicated by a larger dot. Statistical significance was calculated using unpaired Student’s *t* test (***P = 0.0006). The values are represented as the mean ± SEM. **(C)** Schematic representation of the recycling assay showing endocytosis of anti-GFP antibody–labeled SBP-GFP-LAMP1 after 30 min of biotin addition, followed by acid wash to remove non-internalized anti-GFP antibody from the cell surface. Following this, pitstop2 (30 µM) was added to block endocytosis, and cells were incubated for 30 min to allow recycling of internalized anti-GFP antibody–bound SBP-GFP-LAMP1 back to the cell surface. Cells were fixed, and the surface staining was performed using an Alexa Fluor 568–conjugated secondary antibody without permeabilization. The schematic is created using BioRender. **(D)** Recycling assay was performed in HeLa cells treated with control or Arl8b siRNA, and representative micrographs depicting staining of surface-recycled SBP-GFP-LAMP1 are shown. Scale bar: 10 µm. **(E)** Graph represents the relative ratio of surface-recycled signal intensity (measured by anti-GFP antibody) to the total intensity of SBP-GFP-LAMP1 (total GFP signal) (*n* = 3; each dot represents a single experiment). Statistical significance was calculated using the one-sample *t* test (**P = 0.0048). The values are represented as the mean ± SEM.

Next, we assessed RUSH-LAMP1 delivery to active lysosomes upon Arl8b depletion. As previously noted, RUSH-LAMP1 is delivered to active lysosomes, which are generally located in the perinuclear region at 90–120 min of biotin addition (Fig. 3, A and B; and Video 4). In contrast to control cells, Arl8b-depleted cells showed an accumulation of RUSH-LAMP1–positive vesicles near the cell periphery at 90 min after biotin addition with a significant reduction in colocalization with active lysosomes at 120 min after biotin addition (Fig. 3, A and B; and Video 4). Given the peripheral accumulation of RUSH-LAMP1–positive vesicles in Arl8b-depleted cells, we assessed whether there was an enhanced recycling of LAMP1 back to the cell surface. To this end, we performed an antibody-based recycling assay wherein anti-GFP antibody was employed for monitoring the fate of endocytosed RUSH-LAMP1 in control and Arl8b-depleted cells (Fig. 3 C). Pitstop2 was added to both control and Arl8b-depleted cells during the last 30 min of the chase to prevent the re-endocytosis of recycled RUSH-LAMP1. We found that Arl8b depletion resulted in a significant increase in the recycling of RUSH-LAMP1 as compared to the control (Fig. 3, D and E). These findings suggest that Arl8b depletion impairs LAMP1 delivery to active lysosomes, and instead, endocytosed LAMP1 is recycled back to the cell surface.

**Video 4.** **Time-lapse imaging of control and Arl8b siRNA-treated HeLa cells expressing SBP-GFP-LAMP1 and incubated with LTR to label active lysosomes.** The cells were incubated with biotin, and the route of SBP-GFP-LAMP1 trafficking was followed after 90 min of biotin addition. The control and Arl8b siRNA videos are captured at 2.44 frames/sec with no time interval between the frames. The movies are shown at 2 frames/sec, and the total number of frames displayed is 25.

### Arl8b depletion leads to recycling of newly synthesized LAMP1 to the cell surface in a Rab11a-dependent manner

**Figure 4.**
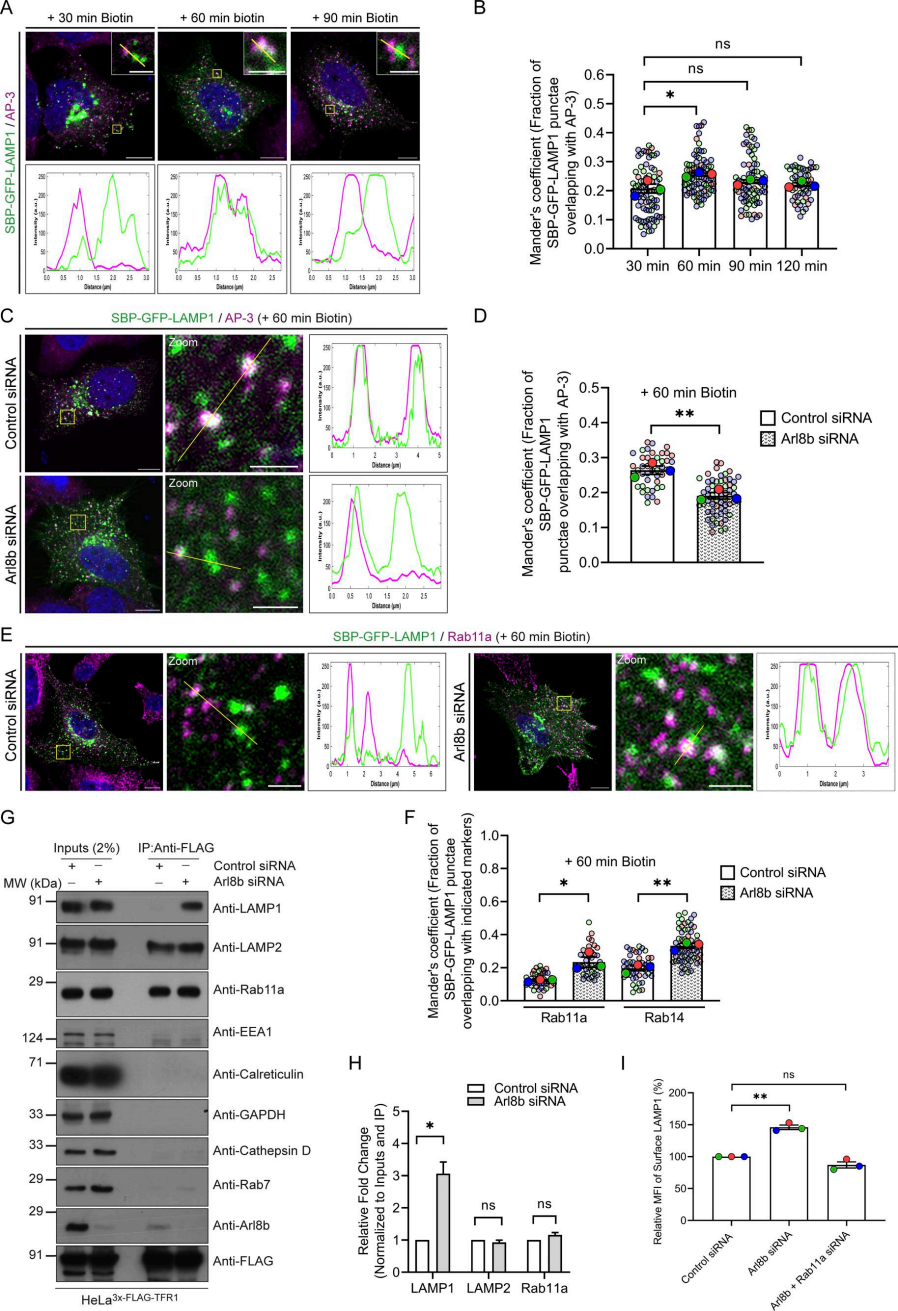
**Arl8b depletion results in LAMP1 missorting to Rab11a-positive REs. (A)** Representative confocal images of HeLa cells expressing SBP-GFP-LAMP1 (green) and stained for endogenous AP-3 (magenta) at the indicated time points after biotin addition. The line profiles indicate fluorescence intensity along the yellow lines for both channels: SBP-GFP-LAMP1 (green) and AP-3 (magenta). Scale bars: 10 μm (main); 2 μm (inset). **(B)** Manders’ colocalization coefficient quantification of SBP-GFP-LAMP1 with AP-3 at the indicated time points after biotin addition is shown from three independent experiments (*n* = 3). Colors in the SuperPlots indicate individual experiments, with each dot representing a single cell. The mean value for each experiment is indicated by a larger dot. Statistical significance was calculated by one-way ANOVA with Dunnett’s multiple comparisons test (*P = 0.0146; n.s., non-significant). The values are represented as the mean ± SEM. **(C)** Representative confocal micrographs of HeLa cells treated with control or Arl8b siRNA, followed by the expression of SBP-GFP-LAMP1 (green). Cells were fixed at 60 min after biotin addition and immunostained for endogenous AP-3 (magenta). The line profiles indicate fluorescence intensity along the yellow lines for both channels: SBP-GFP-LAMP1 (green) and AP-3 (magenta). Scale bars: 10 μm (main); 2 μm (inset). **(D)** Quantification of Manders’ colocalization coefficient of SBP-GFP-LAMP1 with AP-3 in HeLa cells treated with the indicated siRNAs is shown from three independent experiments (*n* = 3). Colors in the SuperPlots indicate individual experiments, with each dot representing a single cell. The mean value for each experiment is indicated by a larger dot. Statistical significance was calculated using unpaired Student’s *t* test (**P = 0.0088). The values are represented as the mean ± SEM. **(E)** Representative confocal images of HeLa cells treated with control or Arl8b siRNA, followed by the expression of SBP-GFP-LAMP1 (green). Cells were fixed at 60 min after biotin addition and immunostained for endogenous Rab11a (magenta). The line profiles indicate fluorescence intensity along the yellow lines for both channels: SBP-GFP-LAMP1 (green) and Rab11a (magenta). Scale bars: 10 μm (main); 2 μm (inset). **(F)** Quantification of Manders’ colocalization coefficient of SBP-GFP-LAMP1 with Rab11a or Rab14 in HeLa cells treated with the indicated siRNAs is shown from three independent experiments (*n* = 3). Colors in the SuperPlots indicate individual experiments, with each dot representing a single cell. The mean value for each experiment is indicated by a larger dot. Statistical significance was calculated using unpaired Student’s *t* test (*P = 0.0237; **P = 0.0028). The values are represented as the mean ± SEM. **(G)** HeLa cells stably expressing 3x-FLAG-TFR1 were treated with control or Arl8b siRNA, and the lysates were subjected to anti-FLAG IP. The precipitates were IB with the indicated antibodies. **(H)** Graph represents the relative fold change in densitometric values of the indicated proteins normalized to input and direct IP of 3x-FLAG-TFR1. Statistical significance was calculated using the one-sample *t* test (*n* = 4; *P = 0.0108; n.s., non-significant). The values are represented as the mean ± SEM. **(I)** Bar graph represents the percent MFI of surface LAMP1 in HeLa cells treated with the indicated siRNAs and normalized to control siRNA (*n* = 3; each dot represents a single experiment). Statistical significance was calculated using the one-sample *t* test (**P = 0.0061; n.s., non-significant). The values are represented as the mean ± SEM. IP, immunoprecipitation; IB, immunoblotted. Source data are available for this figure: SourceData F4.

**Figure S3.**
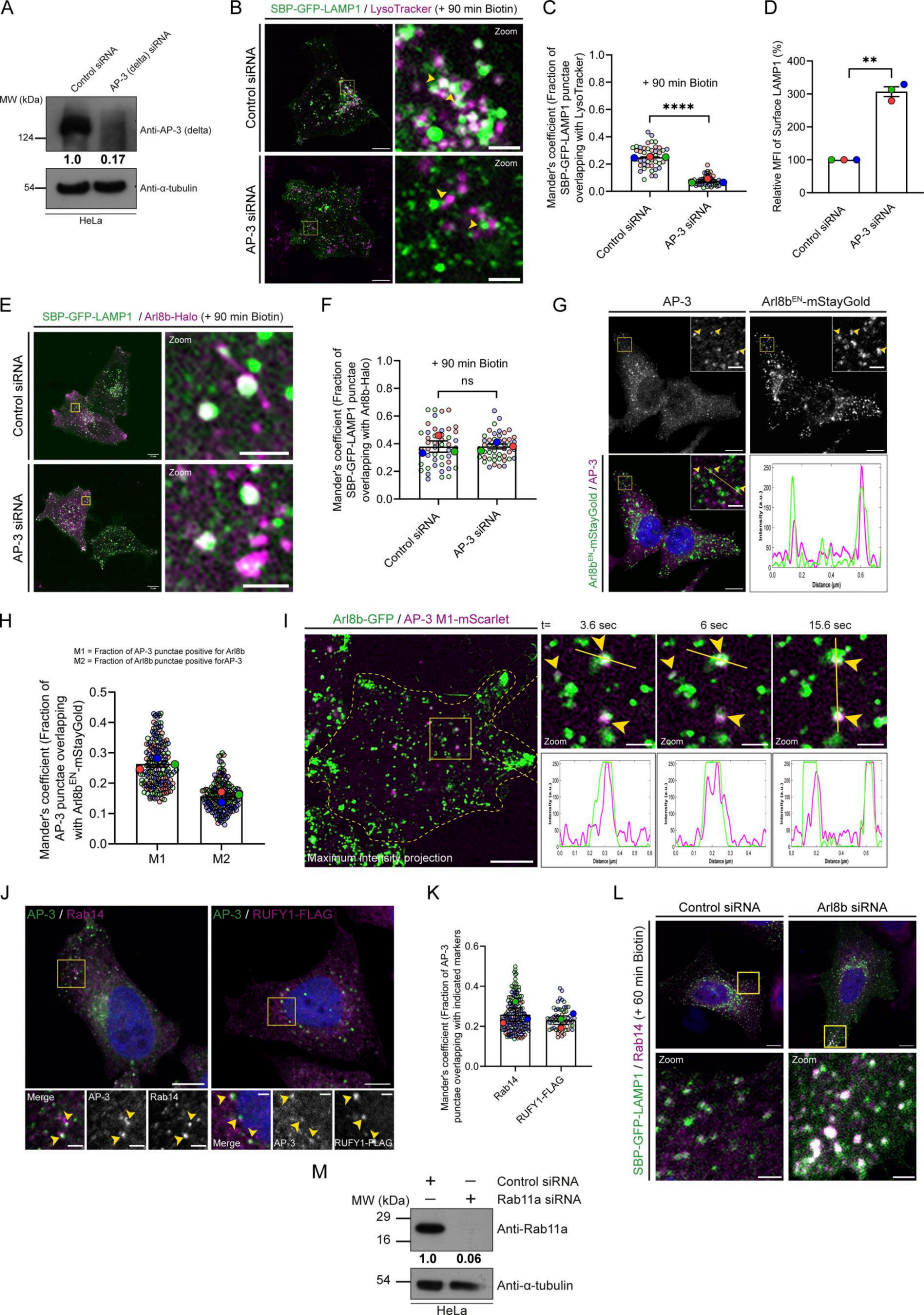
**Arl8b-positive vesicles transiently interact with AP-3–positive endosomes, which represent the sorting station for the newly synthesized LAMP1 to active lysosomes. (A)** HeLa cell lysates treated with control or AP-3 siRNA were IB for the indicated proteins. The values represent densitometric analysis of AP-3 levels normalized to α-tubulin. **(B)** Representative confocal micrographs of HeLa cells treated with control or AP-3 siRNA, followed by the expression of SBP-GFP-LAMP1 (green). Live-cell imaging was performed after cells were incubated with LTR (magenta) dye to label acidic lysosomes, followed by biotin addition. Yellow arrowheads in the insets denote the colocalized pixels. Scale bars: 10 μm (main); 2 μm (inset). **(C)** Manders’ colocalization coefficient quantification of SBP-GFP-LAMP1 with LTR in HeLa cells treated with control or AP-3 siRNA is shown from three independent experiments (*n* = 3). Colors in the SuperPlots indicate individual experiments, with each dot representing a single cell. The mean value for each experiment is indicated by a larger dot. Statistical significance was calculated using unpaired Student’s *t* test (****P < 0.0001). The values are represented as the mean ± SEM. **(D)** Bar graph represents the percent MFI of surface LAMP1 in HeLa cells treated with the indicated siRNAs and normalized to control siRNA (*n* = 3; each dot represents a single experiment). Statistical significance was calculated using the one-sample *t* test (**P = 0.0050). The values are represented as the mean ± SEM. **(E)** Representative confocal images of HeLa cells treated with control or AP-3 siRNA, followed by co-expression of SBP-GFP-LAMP1 (green) and Arl8b-Halo (magenta). Cells were incubated with biotin, and live-cell imaging was performed. Scale bars: 10 μm (main); 2 μm (inset). **(F)** Quantification of Manders’ colocalization coefficient of SBP-GFP-LAMP1 with Arl8b-Halo in HeLa cells treated with the indicated siRNAs is shown from three independent experiments (*n* = 3). Colors in the SuperPlots indicate individual experiments, with each dot representing a single cell. The mean value for each experiment is indicated by a larger dot. Statistical significance was calculated using unpaired Student’s *t* test (n.s., non-significant). The values are represented as the mean ± SEM. (**G)** Representative confocal micrographs of Arl8b^EN^-mStayGold KI (green) HeLa cells immunostained for endogenous AP-3 (magenta). Yellow arrowheads in the insets denote the colocalized pixels. The line profiles indicate fluorescence intensity along the yellow lines for both channels: Arl8b^EN^-mStayGold (green) and AP-3 (magenta). Scale bars: 10 μm (main); 2 μm (inset). **(H)** Quantification of Manders’ colocalization coefficient (M1 and M2) of Arl8b^EN^-mStayGold with AP-3 in HeLa cells is shown from three independent experiments (*n* = 3). Colors in the SuperPlots indicate individual experiments, with each dot representing a single cell. The mean value for each experiment is indicated by a larger dot. The values are represented as the mean ± SEM. **(I)** Representative confocal micrograph from a live-cell imaging experiment of HeLa cells co-expressing Arl8b-GFP (green) and AP-3 (M1)-mScarlet (magenta); insets show time-lapse images. Micrographs are maximum-intensity projections of z-stack images from a live-cell video. Yellow arrowheads in the insets denote the colocalized pixels. The line profiles indicate fluorescence intensity along the yellow lines for both channels: Arl8b-GFP (green) and AP-3 (M1)-mScarlet (magenta). Scale bars: 10 μm (main); 2 μm (inset). **(J)** Representative confocal micrographs of HeLa cells immunostained for endogenous AP-3 (magenta) and Rab14 (green) (left panel). Representative confocal images of HeLa cells expressing RUFY1-FLAG were immunostained with anti-FLAG (green) and anti-AP-3 antibodies (magenta) (right panel). Yellow arrowheads in the insets denote the colocalized pixels. Scale bars: 10 μm (main); 2 μm (inset). **(K)** Quantification of Manders’ colocalization coefficient of AP-3 with Rab14 and RUFY1-FLAG, respectively, from three independent experiments (*n* = 3). Colors in the SuperPlots indicate individual experiments, with each dot representing a single cell. The mean value for each experiment is indicated by a larger dot. The values are represented as the mean ± SEM. **(L)** Representative confocal images of HeLa cells treated with control or Arl8b siRNA, followed by the expression of SBP-GFP-LAMP1 (green). Cells were fixed at 60 min after biotin addition and immunostained for endogenous Rab14 (magenta). Scale bars: 10 μm (main); 2 μm (inset). **(M)** HeLa cell lysates treated with the control or Rab11a siRNAs were IB for the indicated proteins. The values represent densitometric analysis of Rab11a levels normalized to α-tubulin. Source data are available for this figure: SourceData FS3.

To decipher the mechanism by which Arl8b regulates LAMP1 trafficking, we first analyzed LAMP1 colocalization with AP-3, which is the sorting adaptor that mediates LAMP1 sorting from the early endosomes to lysosomes (Chapuy et al., 2008; Ma et al., 2021; Peden et al., 2004). To this end, we first deciphered the kinetics of AP-3 recruitment on RUSH-LAMP1–containing vesicles. We found that endogenous AP-3 colocalized with RUSH-LAMP1 at 60 min after biotin addition, which was modestly reduced by 90 min, consistent with the known role of AP-3 as a sorting adaptor on the early endosomes (Fig. 4, A and B). Expectedly, there was a significant delay in RUSH-LAMP1 delivery to active lysosomes in cells depleted of AP-3 (Fig. S3, A–C). In line with the previous studies (Ivan et al., 2012; Peden et al., 2004), we also observed increased levels of endogenous LAMP1 on the cell surface in AP-3–depleted cells, reinforcing that impaired sorting of LAMP1 from early endosomes leads to its increased recycling to the cell surface (Fig. S3 D). No change in Arl8b colocalization with RUSH-LAMP1 was observed in AP-3 knockdown as compared to the control, supporting our earlier findings that Arl8b recruitment occurs prior to LAMP1 delivery to active lysosomes (Fig. S3, E and F).

Next, to investigate whether Arl8b plays a role in LAMP1 sorting from early endosomes, we first assessed its localization on the AP-3^+^ sorting endosomes. We observed that a subset of Arl8b^EN^-mStayGold-positive vesicles colocalized with endogenous AP-3 (Fig. S3, G and H). We also analyzed the dynamics of Arl8b and AP-3 association by live-cell imaging and found several instances of the Arl8b-positive vesicles docking and interacting with AP-3–positive vesicles (Fig. S3 I and Video 5). The Rab protein, Rab14, and its effector RUFY1, which also binds to Arl8b, are also localized on AP-3^+^ sorting endosomes (Fig. S3, J and K) (Rawat et al., 2023; Yamamoto et al., 2010). RUFY1 has been previously reported to interact with AP-3 (Ivan et al., 2012). These findings suggest that Arl8b interaction with RUFY1 occurs on the AP-3^+^ sorting endosomes and may play a role in the sorting of LAMP1 and other lysosomal cargoes.

**Video 5.** **Time-lapse imaging of HeLa cells co-expressing AP-3 (M1)-mScarlet (magenta) and Arl8b-GFP (green).** The video is captured at 1.34 frames/sec with no time interval between the frames. The video is the maximum-intensity projection of three z-stacks captured. The movies (whole cell and marked inset) are shown at 4 frames/sec, and the total number of frames displayed is 50 for each. The yellow arrows in the inset movie depict the Arl8b-positive vesicles docking and interacting with AP-3–positive endosomes.

We hypothesized that depletion of Arl8b leads to an altered fate of the newly synthesized RUSH-LAMP1 pool, i.e., reduced localization to the AP-3^+^ sorting endosomes and increased recycling to the cell surface. Indeed, we found that RUSH-LAMP1 colocalization with AP-3 was significantly reduced in Arl8b-depleted cells at 60 min after biotin addition (Fig. 4, C and D). Also, consistent with enhanced recycling of RUSH-LAMP1 to the cell surface, its colocalization with the recycling endosomal Rab protein, Rab11a, was significantly increased upon Arl8b depletion (Fig. 4, E and F). Furthermore, RUSH-LAMP1 colocalization with Rab14, which has an overlapping localization and function with Rab11a in receptor recycling, was also enhanced upon Arl8b depletion (Fig. S3 L and Fig. 4 F) (Kelly et al., 2010; Qi et al., 2013).

Finally, to test whether endogenous LAMP1 (similar to RUSH-LAMP1) is also present in REs upon Arl8b depletion, we employed a previously described approach of immuno-IP of transferrin receptor (TfR) to isolate recycling membrane fractions (Hundley et al., 2024). We found that Arl8b knockdown led to a significant increase in endogenous LAMP1 levels in the RE membrane fractions, supporting the findings of the RUSH assay (Fig. 4, G and H). The isolated RE membranes were devoid of markers for active lysosomes (cathepsin D), late endosomes (Rab7), ER (calreticulin), early endosomes (EEA1), and cytosol (GAPDH), confirming the specificity of the assay (Fig. 4 G). Notably, Arl8b was weakly detected in the RE membranes, while the same was not observed for Rab7 or cathepsin D, reinforcing that Arl8b is present in non-lysosomal compartments (Fig. 4 G). We noted that LAMP2 appeared to be more than LAMP1 in REs; moreover, there was no change in LAMP2 levels in the RE membrane fractions upon Arl8b depletion, which is consistent with our previous result that Arl8b knockdown did not affect LAMP2 levels at the cell surface (Fig. 4, G and H). Although the significance of these differential levels of LAMP2 versus LAMP1 in REs is not clear, a previous study had also shown differential kinetics of trafficking of newly synthesized LAMP1 versus LAMP2 to lysosomes (Ecard et al., 2024).

Next, to determine whether LAMP1 recycling to the plasma membrane upon Arl8b depletion is Rab11a-dependent, we performed a dual knockdown of Rab11a along with Arl8b and measured surface LAMP1 levels (Fig. S3 M and Fig. 4 I). Indeed, cells co-depleted of Rab11a and Arl8b showed surface LAMP1 levels comparable to the control, suggesting that LAMP1 recycling to the cell surface in Arl8b depletion is dependent on Rab11a (Fig. 4 I). These findings suggest a model whereby Arl8b recruitment on the LAMP1 sorting subdomain of early/REs inactivates Rab11a-dependent recycling of LAMP1 to the cell surface, facilitating its efficient sorting (and likely of other lysosomal cargo) to active lysosomes.

### Rab11a GAP-TBC1D9B interacts with Arl8b via its N-terminal region encompassing the GRAM1 domain

**Figure S4.**
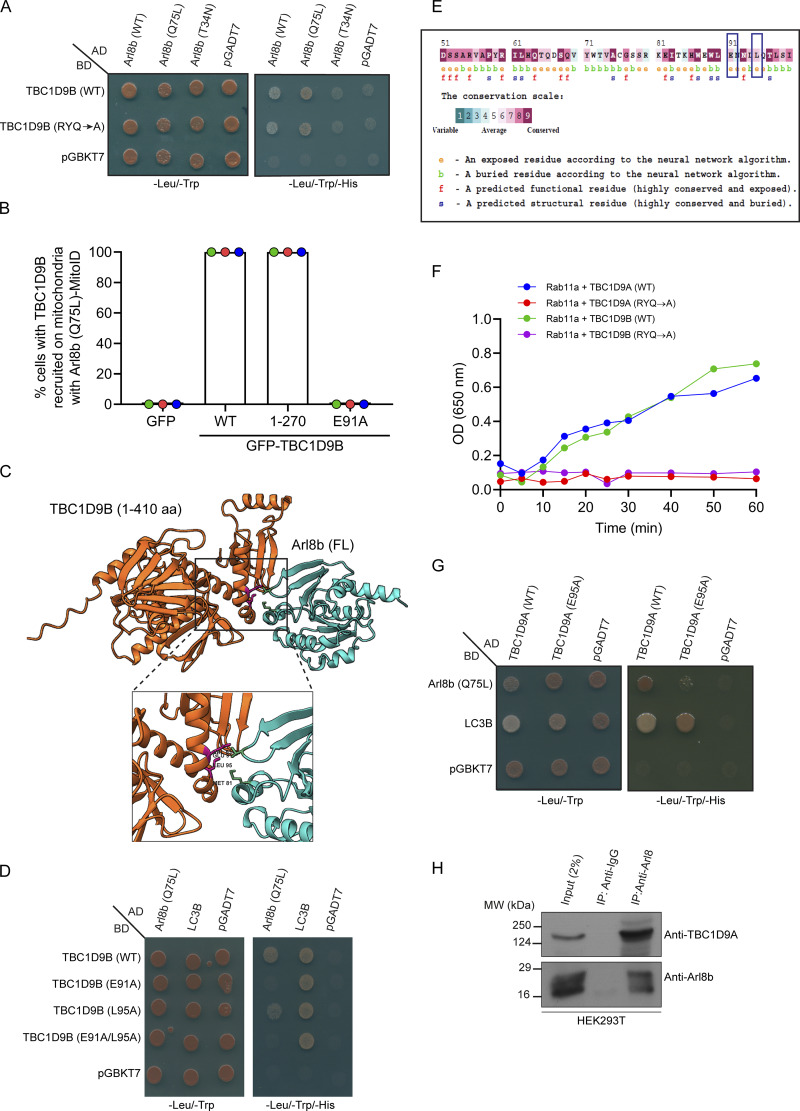
**TBC1D9A is a Rab11a GAP and interacts with Arl8b via its N-terminal region encompassing the GRAM1 domain. (A)** Yeast two-hybrid assay to detect the interaction between TBC1D9B (WT and RYQ →A mutant) and different forms of Arl8b. Co-transformants were spotted on -Leu/-Trp and -Leu/-Trp/-His media to confirm viability and interactions, respectively. **(B)** Percentage of cells showing relocalization of GFP or GFP-TBC1D9B (WT and its mutants) to mitochondria with HA-tagged Arl8b (Q75L)-MitoID (*n* = 3; each dot represents a single experiment). The values are represented as the mean ± SEM. **(C)** Structural model of interactions between Arl8b and the N-terminal fragment of TBC1D9B (1–410 residues) was generated using the AlphaFold3 tool and visualized using Chimera software. The cyan chain denotes Arl8b, and the orange chain indicates TBC1D9B (1–410 residues). **(D)** Yeast two-hybrid assay to detect interaction of TBC1D9B (WT or indicated mutants) with Arl8b (Q75L). Co-transformants were spotted on -Leu/-Trp and -Leu/-Trp/-His media to confirm viability and interactions, respectively. In the assay, LC3B was used as a positive control for binding to TBC1D9B. **(E)** ConSurf server tool was used to predict the evolutionary conservation score (represented by a color-coded scale) of Arl8b binding–defective mutants of TBC1D9B (highlighted in blue) predicted by the AlphaFold3 server. **(F)** Kinetic analysis of GTP hydrolysis activity of Rab11a in the presence of TBC1D9A (WT or RYQ→A) or TBC1D9B (WT or RYQ→A) was performed by measuring the release of free inorganic phosphate (P_i_) using malachite green reagent. Absorbance was recorded at 650 nm, and plotted values were calculated by subtracting the signal of GDP-loaded samples from GTP-loaded samples. **(G)** Yeast two-hybrid assay was performed to check the interaction of Arl8b (Q75L) and LC3B with TBC1D9A (WT or E95A). The co-transformants were spotted on -Leu/-Trp and -Leu/-Trp/-His media to confirm viability and interactions, respectively. **(H)** Endogenous immunoprecipitation was performed by incubating HEK293T cell lysates with an anti-Arl8 antibody, followed by immunoblotting with the indicated antibodies. Source data are available for this figure: SourceData FS4.

**Figure 5.**
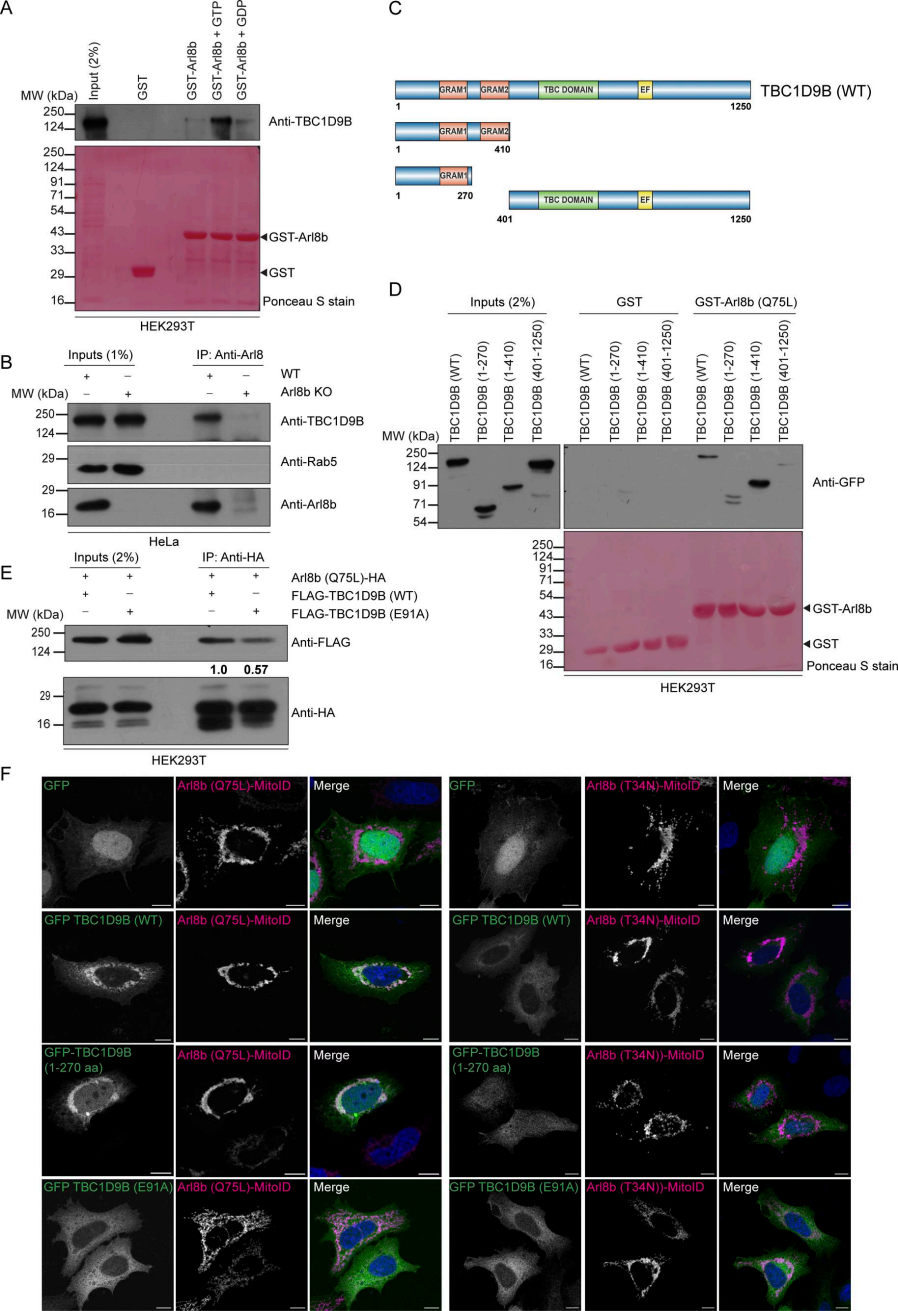
**TBC1D9B interacts with Arl8b via its N-terminal region encompassing the GRAM1 domain. (A)** GST and GST-Arl8b proteins were immobilized on glutathione resins and loaded with GTP or GDP, followed by incubation with HEK293T cell lysates. The precipitates were IB with anti-TBC1D9B antibody, and Ponceau S staining was done to visualize purified proteins. **(B)** WT and Arl8b KO HeLa cell lysates were subjected to endogenous IP using anti-Arl8b antibody, followed by IB with the indicated antibodies. **(C)** Domain architecture of TBC1D9B and its domain deletion mutants showing N-terminal GRAM1 and GRAM2 domains, TBC domain, and C-terminal EF hand. The image was created using IBS 2.0: Illustrator for Biological Sequences. **(D)** GST and GST-Arl8b (Q75L) proteins were immobilized on glutathione resins, followed by incubation with HEK293T cell lysates expressing GFP-tagged TBC1D9B or its domain deletion mutants. The precipitates were IB with anti-GFP antibody, and Ponceau S staining was done to visualize purified proteins. **(E)** HEK293T cell lysates expressing the indicated proteins were immunoprecipitated with anti-HA antibody and IB with the indicated antibodies. The values represent densitometric analysis of indicated proteins normalized to input and direct IP of Arl8b (Q75L)-HA. **(F)** HeLa cells were co-transfected with GFP alone or GFP-TBC1D9B (WT, 1–270 aa, or E91A) with Arl8b (Q75L or T34N)-MitoID (HA-tagged) and immunostained with an anti-HA (magenta) antibody. Scale bar: 10 µm. Source data are available for this figure: SourceData F5.

We conducted a literature survey of known and potential Arl8b interaction partners or effectors to gain insights into how Arl8b could promote inactivation of Rab11a. Interestingly, TBC1D9B, a well-characterized GAP for Rab11a, was reported as a putative Arl8b interaction partner (Gallo et al., 2014; Garg et al., 2011b; Keren-Kaplan et al., 2022; Nishino et al., 2019; Quirion et al., 2024). Using yeast two-hybrid and GST-pulldown assays, we found that TBC1D9B showed a preferential binding to the constitutively GTP-bound mutant of Arl8b (Q75L) and the GTP-loaded Arl8b (WT) purified protein, as compared to Arl8b (WT), respectively (Fig. S4 A and Fig. 5 A). In contrast, minimal binding was observed with the constitutively GDP-bound mutant of Arl8b (T34N) and with the GDP-loaded Arl8b (WT) purified protein (Fig. S4 A and Fig. 5 A).

We also mutated the Arg (R) and Gln (Q) finger within the IxxDxxR and YxQ motifs, respectively, that are critical for TBC domain–mediated GTP hydrolysis of Rabs (Pan et al., 2006). Notably, we observed that GAP-deficient TBC1D9B mutant (R559A/Y592A/Q594A, referred to as RYQ→A) showed a similar binding to Arl8b as TBC1D9B WT (Fig. S4 A). Next, we validated the interaction between TBC1D9B and Arl8b at endogenous expression levels by the co-immunoprecipitation (co-IP) assay. Endogenous TBC1D9B, but not Rab5 (used as a negative control), co-precipitated with Arl8b, while no co-IP was observed from Arl8b KO HeLa cell lysates, ruling out the possibility that the binding was non-specific in nature (Fig. 5 B).

To narrow down the domain of TBC1D9B required for interaction with Arl8b, we created N-terminal and C-terminal domain–deletion mutants of TBC1D9B and performed a GST-pulldown assay using GST-Arl8b (Q75L) as bait (Fig. 5 C). We observed that the N-terminal GRAM1 domain–containing region (1–270 amino acids [aa]) was sufficient for Arl8b binding; moreover, the binding was strengthened when both the GRAM domains (1–410 aa) were present, whereas minimal binding was observed with the TBC1D9B mutant (401–1250 aa) lacking the N-terminal GRAM domain–containing region (Fig. 5 D).

We next corroborated these results with the recently described mitochondrial relocalization approach for identification of effectors of small GTPases (Gillingham et al., 2019). The mito-localized constitutively GTP-bound form of Arl8b (Q75L), but not its constitutively GDP-bound form (T34N), was sufficient to recruit TBC1D9B to mitochondria (Fig. 5 F and Fig. S4 B). Consistent with our previous finding, the GRAM1 domain–containing region of TBC1D9B was recruited to mitochondria by the mito-localized Arl8b (Q75L) form but not by the Arl8b (T34N) form (Fig. 5 F and Fig. S4 B). Next, we employed the AlphaFold3 structure prediction tool (Abramson et al., 2024) to determine the binding interface residues of Arl8b in complex with the 1–410 aa fragment of TBC1D9B that showed better binding than 1–270 aa (Fig. S4 C). This analysis identified E91 and L95 of TBC1D9B as two key residues mediating interaction with Arl8b. Notably, mutating residue E91 to alanine (E91A) reduced the binding affinity of TBC1D9B for Arl8b, as demonstrated by yeast two-hybrid and co-IP assays (Fig. S4 D and Fig. 5 E). Moreover, recruitment of the E91A mutant of TBC1D9B to mitochondria was not observed with the mito-localized Arl8b (Q75L) form, supporting its lack of binding to Arl8b (Fig. 5 F and Fig. S4 B). Mutation of another predicted binding interface residue, L95 to alanine (L95A), also reduced Arl8b binding but less significantly in comparison with the E91A mutation (Fig. S4 D). These mutants retained their ability to bind LC3B, which was previously shown to interact with TBC1D9B and was employed here as a positive control (Fig. S4 D) (Liao et al., 2018). E91 and L95 residues exhibit strong evolutionary conservation with a score of 7, as predicted by the ConSurf tool (Berezin et al., 2004); moreover, E91A and L95A mutations showed high AlphaMissense (Tordai et al., 2024) scores of 0.8 and 0.9, respectively, reinforcing the functional significance of the two residues (Fig. S4 E and Table S1).

TBC1D9B shares a 62% identity and 74% similarity at the protein level with its paralog TBC1D9A (Table S2). Using an *in vitro* GAP assay, we verified that TBC1D9A, similar to TBC1D9B, also enhanced the intrinsic GTP hydrolysis activity of Rab11a (Fig. S4 F). Additionally, as expected, the GAP-defective mutants of TBC1D9A (R566A/Y599A/Q601A) and TBC1D9B (R559A/Y592A/Q594A) did not show the catalytic activity against Rab11a (Fig. S4 F). Notably, we found that TBC1D9A also interacts with Arl8b both by yeast two-hybrid assay and co-IP of endogenous proteins (Fig. S4, G and H). Moreover, mutation of E95 to alanine in TBC1D9A, which is homologous to the E91 residue of TBC1D9B, resulted in highly reduced binding to Arl8b, but not to LC3B, a known TBC1D9A-binding partner (Fig. S4 G) (Popovic et al., 2012). This suggests that both TBC1D9 paralogs are Arl8b-binding partners and have a similar binding interface for interaction with Arl8b.

### Arl8b recruits TBC1D9B to peripheral non-acidic LAMP1-positive vesicles

**Figure 6.**
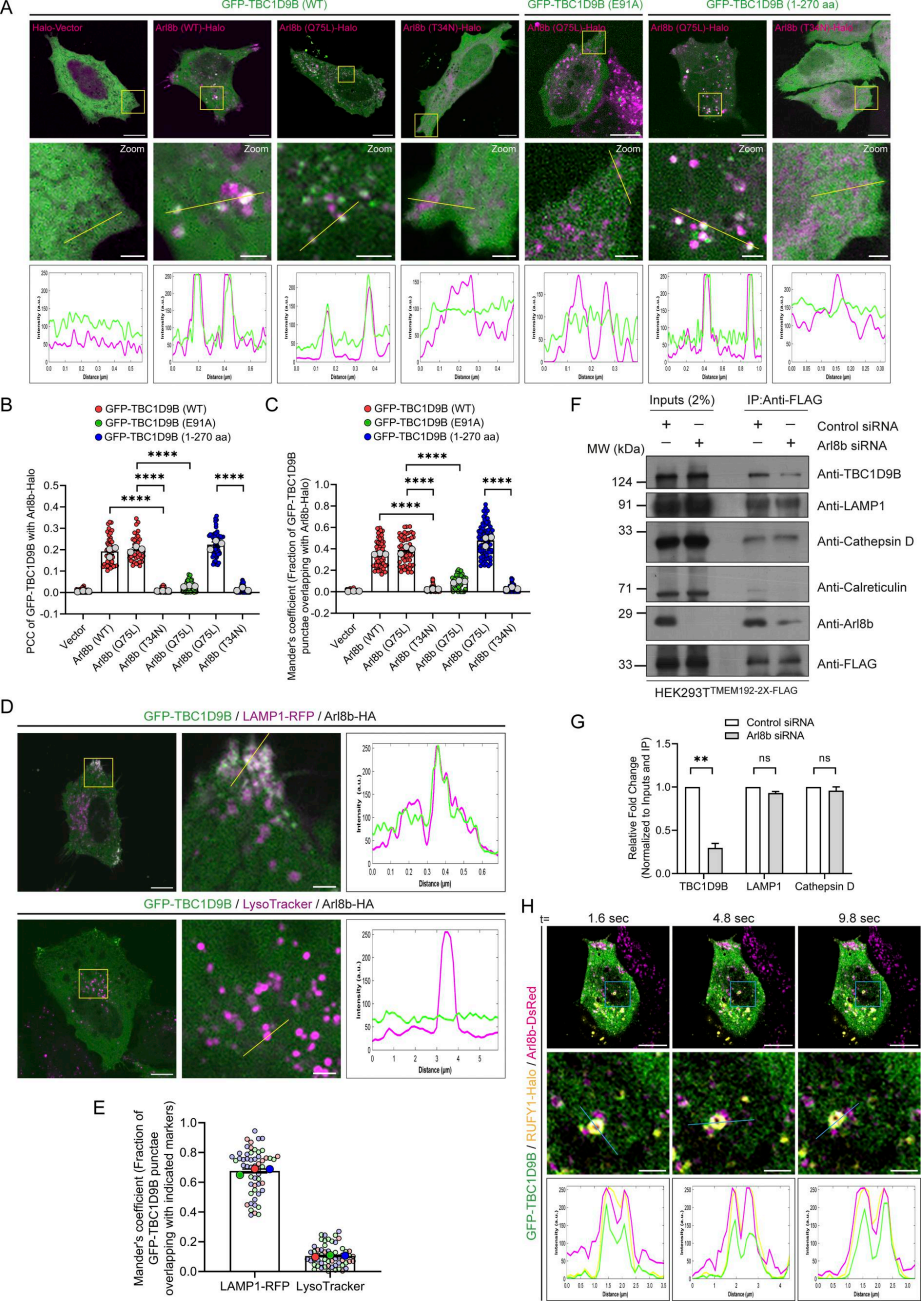
**TBC1D9B localizes on peripheral non-acidic LAMP1-positive vesicles in an Arl8b-dependent manner. (A)** Representative confocal micrographs of HeLa cells co-transfected with GFP-TBC1D9B (WT, E91A, or 1–270 aa) with Arl8b (WT, Q75L, or T34N)-Halo, followed by live-cell imaging. Micrographs are maximum-intensity projections of z-stack images from live-cell video. The line profiles indicate fluorescence intensity of GFP-TBC1D9B (green) and Arl8b-Halo (magenta) along the yellow line. Scale bars: 10 µm (main); 2 µm (inset). **(B)** Quantification of PCC of GFP-TBC1D9B with Arl8b-Halo is shown from three independent experiments (*n* = 3). Colors in the SuperPlots indicate GFP-TBC1D9B (WT, E91A, or 1–270 aa), with each dot representing a single cell. The mean value for each experiment is indicated by a larger dot. Statistical significance was calculated by one-way ANOVA with Tukey’s multiple comparisons test (****P < 0.0001). The values are represented as the mean ± SEM. **(C)** Quantification of Manders’ colocalization coefficient of GFP-TBC1D9B with Arl8b-Halo is shown from three independent experiments (*n* = 3). Colors in the SuperPlots indicate GFP-TBC1D9B (WT, E91A, or 1–270 aa), with each dot representing a single cell. The mean value for each experiment is indicated by a larger dot. Statistical significance was calculated by one-way ANOVA with Tukey’s multiple comparisons test (****P < 0.0001). The values are represented as the mean ± SEM. **(D)** Representative images of HeLa cells co-expressing GFP-TBC1D9B and LAMP1-RFP (upper image)/LTR (lower image) with Arl8b-HA (not shown). The line profiles indicate fluorescence intensity of GFP-TBC1D9B (green) and LAMP1-RFP or LTR (magenta) along the yellow line. Scale bars: 10 µm (main); 2 µm (inset). **(E)** Quantification of Manders’ colocalization coefficient of GFP-TBC1D9B with indicated markers is shown from three independent experiments (*n* = 3). Colors in the SuperPlots indicate individual experiments, with each dot representing a single cell. The mean value for each experiment is indicated by a larger dot. The values are represented as the mean ± SEM. **(F)** HEK293T cells stably expressing TMEM192-2x-FLAG were treated with control or Arl8b siRNA, and lysates were subjected to anti-FLAG IP. The precipitates were IB with the indicated antibodies. **(G)** Bar graph represents the relative fold change in densitometric values of the indicated protein normalized to input and direct IP of TMEM192-2x-FLAG. Statistical significance was calculated using the one-sample *t* test (*n* = 3; **P = 0.0054; n.s., non-significant). The values are represented as the mean ± SEM. **(H)** Representative time-lapse confocal micrograph of a HeLa cell, co-expressing GFP-TBC1D9B (green), RUFY1-Halo (yellow), and Arl8b-DsRed (magenta). Micrographs are maximum-intensity projections of z-stack images from a live-cell video. The line profiles indicate fluorescence intensity along the blue lines for all channels: GFP-TBC1D9B (green), RUFY1-Halo (yellow), and Arl8b-DsRed (magenta). Scale bars: 10 µm (main); 2 µm (inset). Source data are available for this figure: SourceData F6.

To investigate the role of Arl8b binding in regulating membrane localization of TBC1D9B, we employed epitope-tagged constructs due to the unavailability of commercial antibodies that work in immunofluorescence. In accordance with the canonical role of small G proteins in recruiting their effectors, GTP-bound Arl8b was required for TBC1D9B membrane localization, as GFP-tagged TBC1D9B was completely cytosolic when expressed alone (with the vector control) but was recruited to punctate structures in the presence of WT and the constitutively GTP-bound (Q75L) form of Arl8b (Fig. 6 A). This was also reflected in the colocalization coefficient quantification of TBC1D9B (WT) with vector (control) and Arl8b WT or its Q75L mutant (Fig. 6, B and C). In contrast, minimal or no colocalization of TBC1D9B (WT) was observed with the constitutively GDP-bound (T34N) form of Arl8b (Fig. 6, A–C). Consistent with the previously described results, the Arl8b binding–defective mutant of TBC1D9B (TBC1D9B (E91A)) was cytosolic even in the presence of the constitutively GTP-bound form of Arl8b (Fig. 6, A–C). We noted that in comparison with the WT version that localized only to a minor subset of peripheral Arl8b^+^ vesicles, recruitment of the GRAM domain–containing region (1–270 aa) of TBC1D9B with Arl8b (Q75L) was visibly better (Fig. 6 A). This might indicate that other domains of TBC1D9B, such as the TBC domain or EF-hand motifs, might play a regulatory role in determining interaction with Arl8b.

Arl8b localizes to both acidic and degradative as well as non-acidic peripheral LAMP1-positive vesicles (Johnson et al., 2016; Khatter et al., 2015a; Marwaha et al., 2017; Rawat et al., 2023). Intriguingly, we found that TBC1D9B showed colocalization with LAMP1 but not with LTR^+^ vesicles, suggesting that TBC1D9B localizes to the non-acidic Arl8b- and LAMP1-positive peripheral vesicles (Fig. 6 D and E; and Video 6). Next, we isolated lysosomal membranes using the Lyso-IP method from control and Arl8b-depleted cells to establish whether Arl8b is required for TBC1D9B localization to LAMP1^+^ membranes under physiological expression levels (Abu-Remaileh et al., 2017). As shown in Fig. 6, F and G, endogenous TBC1D9B was present in Lyso-IP fractions, and its levels were significantly reduced upon Arl8b depletion, corroborating that Arl8b recruits TBC1D9B on membranes containing lysosomal proteins.

**Video 6.** **Time-lapse imaging of HeLa cells expressing Arl8b-HA (not stained) and GFP-TBC1D9B and either transfected with LAMP1-RFP or incubated with LTR to label active lysosomes.** The videos are captured at 2.04 frames/sec with no time interval between the frames. The movies are shown at 4 frames/sec, and the total number of frames displayed is 25 each.

**Figure S5.**
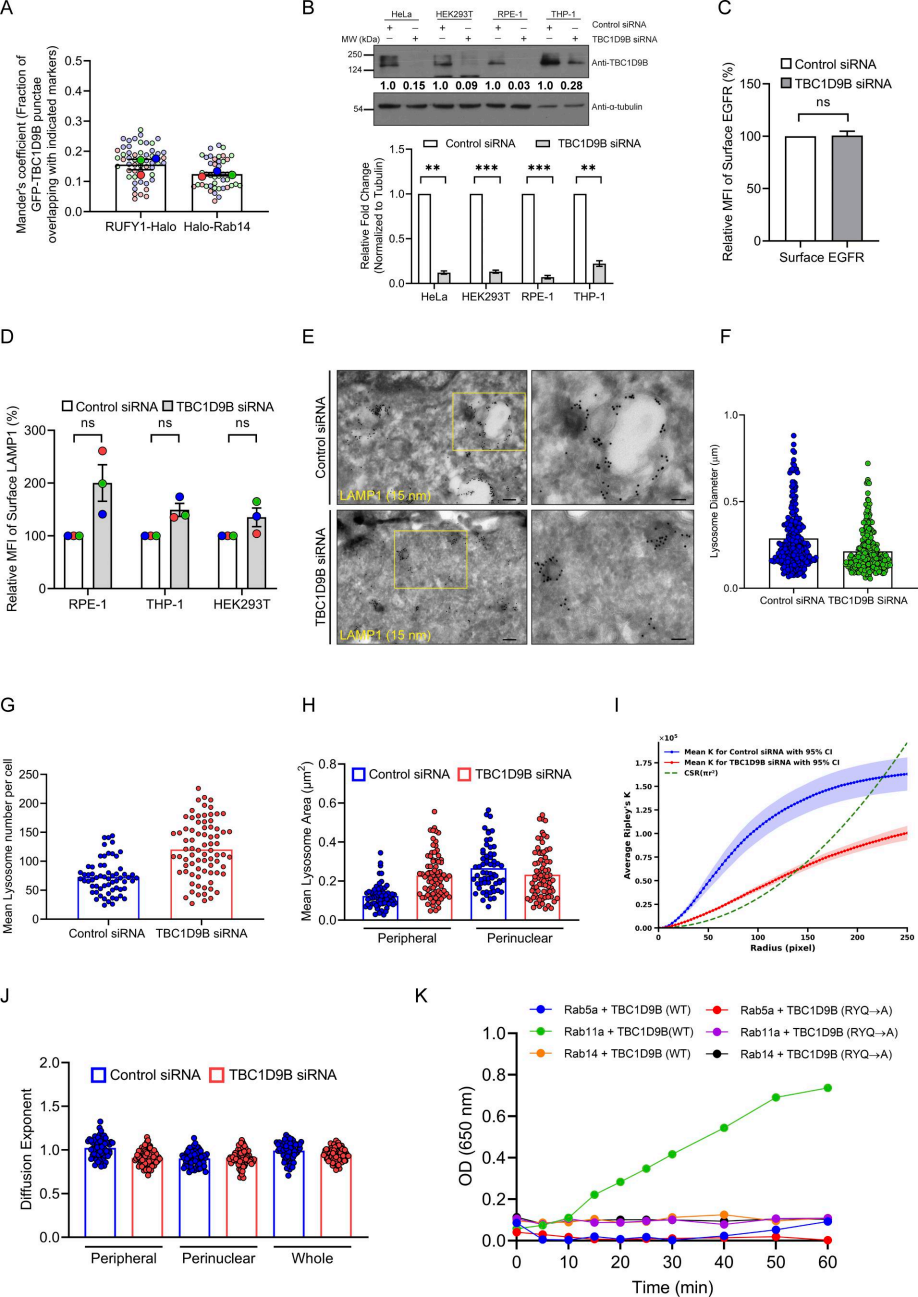
**TBC1D9B depletion leads to an increase in surface LAMP1 levels and alters the number, size, and distribution of lysosomes. (A)** Quantification of Manders’ colocalization coefficient of GFP-TBC1D9B with indicated markers is shown from three independent experiments (*n* = 3). Colors in the SuperPlots indicate individual experiments, with each dot representing a single cell. The mean value for each experiment is indicated by a larger dot. The values are represented as the mean ± SEM. **(B)** Lysates of HeLa, HEK293T, RPE-1, and THP-1 cells treated with control or TBC1D9B siRNA were IB with the indicated antibodies. The values indicate densitometric analysis of TBC1D9B levels normalized to α-tubulin. The bar graph represents the relative fold change in densitometric values of TBC1D9B normalized to α-tubulin. Statistical significance was calculated using the one-sample *t* test (*n* = 3; **P = 0.0011 [HeLa]; ***P = 0.0004 [HEK293T]; ***P = 0.0007 pRPE-1]; **P = 0.007 [THP-1]). The values are represented as the mean ± SEM. **(C)** Bar graph represents the percent MFI of surface EGFR levels in HeLa cells treated with indicated siRNAs and normalized to control siRNA (*n* = 3). Statistical significance was calculated using the one-sample *t* test (n.s., non-significant). The values are represented as the mean ± SEM. **(D)** Graph represents the MFI of surface LAMP1 levels normalized to control siRNA (*n* = 3; each dot represents a single experiment). Statistical significance was calculated using the one-sample *t* test (n.s., non-significant). The values are represented as the mean ± SEM. **(E)** Representative immuno-EM images of HeLa cells treated with control or TBC1D9B siRNA and immunolabeled for LAMP1 (15 nm). Higher magnifications of LAMP1-positive vesicles are shown in the panels on the right. Scale bars: 200 nm (main); 100 nm (inset). **(F)** Lysosome diameter was quantified from immuno-EM images of HeLa cells treated with the indicated siRNAs (*n* = 1; 14 and 16 micrographs of control siRNA and TBC1D9B siRNA-treated cells, respectively). The values are represented as the mean. **(G–J)** HeLa cells treated with control or TBC1D9B siRNA were incubated overnight with Alexa Fluor 568–conjugated dextran, followed by a 6-h chase to label lysosomes, and live-cell imaging was performed. The graph (G) represents the mean lysosome number per cell in control or TBC1D9B-depleted cells analyzed from two independent experiments (*n* = 2; each dot represents a single cell). The values are represented as the mean. The graph (H) represents the mean lysosome area of peripheral and perinuclear lysosomes in control or TBC1D9B siRNA-treated HeLa cells analyzed from two independent experiments (*n* = 2; each dot represents a single cell). The values are represented as the mean. The graph (I) represents Ripley’s constant analysis. The green line depicts random distribution, whereas the blue and red lines denote control and TBC1D9B siRNA-treated samples, respectively. The graph **(J)** represents the diffusion exponent of dextran-loaded terminal lysosomes in control or TBC1D9B siRNA-treated cells and analyzed from two independent experiments (*n* = 2; each dot represents a single cell). The values are represented as the mean. **(K)** Kinetic analysis of GTPase activity for Rab5a, Rab11a, and Rab14 in the presence of TBC1D9B (WT) or its GAP-deficient mutant (RYQ→A). The plotted values were calculated by subtracting the signal of GDP-loaded samples from GTP-loaded samples. Source data are available for this figure: SourceData FS5.

As a subpopulation of Arl8b also localizes to Rab14^+^/AP-3^+^/RUFY1^+^ endosomes that mark the LAMP1 sorting vesicles (Rawat et al., 2023), we next analyzed whether TBC1D9B also localizes to this LAMP1 sorting compartment. Indeed, we found that TBC1D9B, along with Arl8b, was present on a subset of RUFY1- and Rab14-positive endosomes (Fig. 6 H, Fig. S5 A, and Video 7). Interestingly, a previous study has shown that TBC1D9B interacts with Rab4, an early endosomal G protein that also interacts with RUFY1 and colocalizes with Rab14 (Cormont et al., 2001; Gallo et al., 2014; Yamamoto et al., 2010). As TBC1D9B is not a GAP for Rab4 (Gallo et al., 2014), it would be exciting to test whether Rab4 regulates TBC1D9B recruitment to early endocytic vesicles where the LAMP1 sorting machinery is also localized.

**Video 7.** **Time-lapse imaging of HeLa cells expressing GFP-TBC1D9B (green), Arl8b-DsRed (magenta), and either RUFY1-Halo or Halo-Rab14 (yellow).** The videos are captured at 1 frame/sec with no time interval between the frames. The movies are shown at 4 frames/sec, and the total number of frames displayed is 25 each.

### TBC1D9B inactivates Rab11a for sorting of newly synthesized LAMP1 to active lysosomes

**Figure 7.**
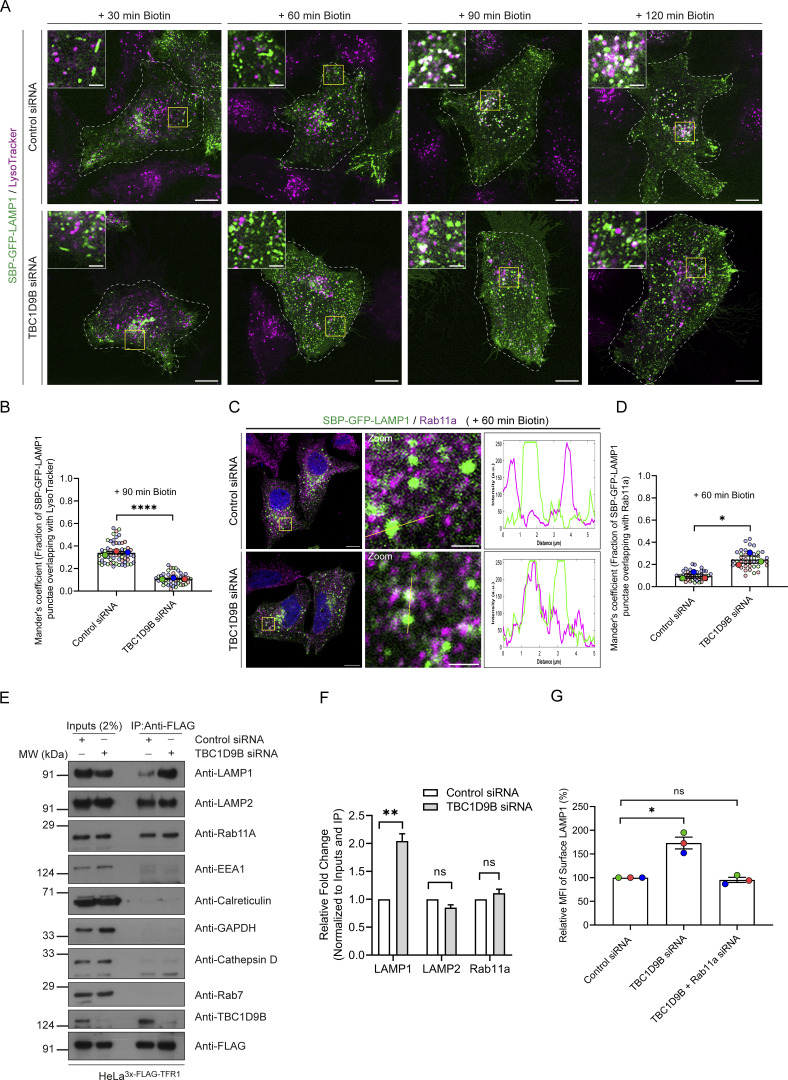
**TBC1D9B inactivates Rab11a for sorting of the newly synthesized LAMP1 to active lysosomes. (A)** Representative confocal micrographs of HeLa cells treated with control or TBC1D9B siRNA and transfected with SBP-GFP-LAMP1. Cells were labeled with LTR (magenta) followed by biotin addition and imaged at the indicated time points. Scale bars: 10 µm (main); 2 μm (inset). **(B)** Quantification of Manders’ colocalization coefficient of SBP-GFP-LAMP1 with LTR in HeLa cells treated with indicated siRNAs is shown from three independent experiments (*n* = 3). Colors in the SuperPlots indicate individual experiments, with each dot representing a single cell. The mean value for each experiment is indicated by a larger dot. Statistical significance was calculated using unpaired Student’s *t* test (****P < 0.0001). The values are represented as the mean ± SEM. **(C)** Representative confocal images of HeLa cells treated with control or TBC1D9B siRNA and expressing SBP-GFP-LAMP1. Cells were fixed at 60 min after biotin addition and immunostained for endogenous Rab11a (magenta). The line profiles indicate fluorescence intensity along the yellow lines for both channels: SBP-GFP-LAMP1 (green) and Rab11a (magenta). Scale bars: 10 μm (main); 2 μm (inset). **(D)** Manders’ colocalization coefficient quantification of SBP-GFP-LAMP1 with Rab11a in HeLa cells treated with the indicated siRNAs is shown from three independent experiments (*n* = 3). Colors in the SuperPlots indicate individual experiments, with each dot representing a single cell. The mean value for each experiment is indicated by a larger dot. Statistical significance was calculated using unpaired Student’s *t* test (*P = 0.0168). The values are represented as the mean ± SEM. **(E)** HeLa cells stably expressing 3x-FLAG-TFR1 were treated with control or TBC1D9B siRNA, and lysates were subjected to anti-FLAG IP. The precipitates were IB with the indicated antibodies. **(F)** Bar graph represents the relative fold change in densitometric values of the indicated protein normalized to input and direct IP of 3x-FLAG-TFR1. Statistical significance was calculated using the one-sample *t* test (*n* = 4; **P = 0.0039; n.s., non-significant). The values are represented as the mean ± SEM. **(G)** Bar graph represents percent MFI of surface LAMP1 in HeLa cells treated with indicated siRNAs and normalized to control siRNA (*n* = 3; each dot represents a single experiment). Statistical significance was calculated using the one-sample *t* test (*P = 0.0275; n.s., non-significant). The values are represented as the mean ± SEM. Source data are available for this figure: SourceData F7.

Based on the results thus far, we hypothesize that Arl8b recruits TBC1D9B on newly synthesized LAMP1 cargo–containing vesicles to mediate Rab11a inactivation for regulating LAMP1 recycling to the cell surface. To investigate TBC1D9B role in LAMP1 trafficking, we followed the fate of RUSH-LAMP1 vesicles upon biotin addition in TBC1D9B-depleted cells (knockdown efficiency was confirmed to be >90%, as shown in Fig. S5 B). We found that there was a significant delay in RUSH-LAMP1 delivery to the active lysosomes marked by LTR (Fig. 7, A and B; and Video 8). Importantly, akin to Arl8b depletion, there was an accumulation of peripheral RUSH-LAMP1 vesicles positive for Rab11a in TBC1D9B-depleted cells (Fig. 7, C and D).

**Video 8.** **Time-lapse imaging of control and TBC1D9B siRNA-treated HeLa cells expressing SBP-GFP-LAMP1 and incubated with LTR to label active lysosomes.** The videos are captured at 1.42 frames/sec with no time interval between the frames. The movies are shown at 2 frames/sec, and the total number of frames displayed is 25.

Consistent with the evidence showing TBC1D9B depletion abrogates RUSH-LAMP1 delivery to active lysosomes, endogenous LAMP1 was significantly more in the RE membrane fractions in TBC1D9B-depleted cells as compared to the control (Fig. 7, E and F). In line with its role as a Rab11a GAP, TBC1D9B was also enriched in the RE membrane fractions (Fig. 7 E). Corroborating this observation, LAMP1 levels at the cell surface, but not of EGFR (used as a control), were increased upon TBC1D9B knockdown (Fig. 7 G and Fig. S5 C). Additionally, co-depletion of Rab11a along with TBC1D9B reverted the increased surface LAMP1 phenotype observed in cells depleted of TBC1D9B alone (Fig. 7 G). Similar to Arl8b depletion, TBC1D9B depletion also led to an increased surface LAMP1 in RPE-1 cells, while a modest but not significant increase was observed in HEK293T cells (Fig. S5 D). In THP-1 macrophages, we found a modest but not significant increase in surface LAMP1 levels; however, the knockdown efficiency in these cells was less as compared to other cell lines (Fig. S5, B and D). Taken together, these findings suggest that Arl8b effector TBC1D9B mediates inactivation of Rab11a, regulating LAMP1 recycling to the cell surface.

### TBC1D9B depletion alters lysosome characteristics, impairs cathepsin processing, and results in impaired cargo degradation

**Figure 8.**
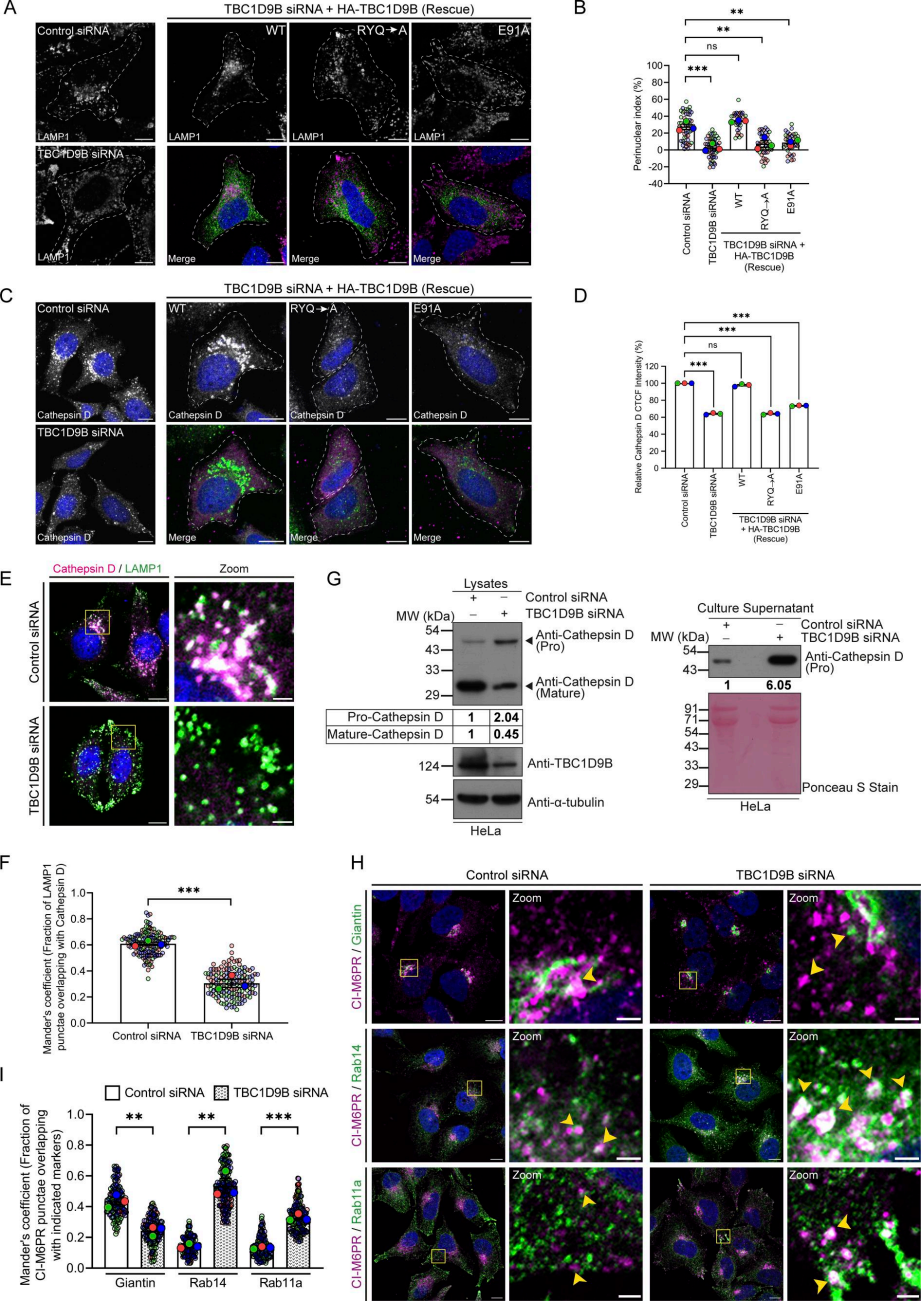
**TBC1D9B depletion alters lysosome characteristics and impairs CI-M6PR retrieval to the TGN, compromising cathepsin processing. (A)** Representative confocal micrographs of HeLa cells treated with control or TBC1D9B siRNA and transfected with the HA-TBC1D9B (WT, RYQ→A, or E91A rescue) construct. Cells were fixed and immunostained with anti-HA (green) and anti-LAMP1 (magenta) antibodies. Scale bar: 10 μm. **(B)** Quantification of the perinuclear index of LAMP1-positive compartments in HeLa cells treated with control or TBC1D9B siRNA and transfected with the HA-TBC1D9B (WT, RYQ→A, or E91A rescue) construct from three independent experiments (*n* = 3); colors in the SuperPlots indicate individual experiments, with each dot representing a single cell. The mean value for each experiment is indicated by a larger dot. Statistical significance was calculated by one-way ANOVA with Dunnett’s multiple comparisons test (***P = 0.0003; **P = 0.0013 (RYQ→A); **P = 0.0023 (E91A); n.s., non-significant). The values are represented as the mean ± SEM. **(C)** Representative confocal images of HeLa cells treated with control or TBC1D9B siRNA and transfected with the HA-TBC1D9B (WT, RYQ→A, or E91A rescue) construct. Cells were fixed and immunostained with anti-HA (magenta) and anti-cathepsin D (green) antibodies. Scale bar: 10 μm. **(D)** The graph shows the relative percentage of CTCF values of the cathepsin D signal normalized to control siRNA (*n* = 3; each dot represents a single experiment). Statistical significance was calculated using the one-sample *t* test (***P = 0.0003 (TBC1D9B siRNA and RYQ→A); ***P = 0.0001 (E91A); n.s., non-significant). The values are represented as the mean ± SEM. **(E)** Representative confocal images of HeLa cells treated with control or TBC1D9B siRNA and immunostained with anti-LAMP1 (green) and anti-cathepsin D (magenta) antibodies. Scale bars: 10 μm (main); 2 μm (inset). **(F)** Manders’ colocalization coefficient quantification of LAMP1 with cathepsin D in HeLa cells treated with the indicated siRNAs from three independent experiments (*n* = 3). Colors in the SuperPlots indicate individual experiments, with each dot representing a single cell. The mean value for each experiment is indicated by a larger dot. Statistical significance was calculated using unpaired Student’s *t* test (***P = 0.0008). The values are represented as the mean ± SEM. **(G)** Western blot analysis of cathepsin D levels in lysates and the culture supernatant was performed in control and TBC1D9B siRNA-treated HeLa cells. The Ponceau S stain was done to visualize protein levels. The values represent densitometric analysis of cathepsin D levels normalized to α-tubulin (lysate) or total protein (media). **(H)** Representative confocal micrographs of HeLa cells treated with control or TBC1D9B siRNA and costained for CI-M6PR (magenta) and Giantin (green) or Rab14 (green) or Rab11a (green). The yellow arrowheads denote colocalized pixels. Scale bars: 10 μm (main); 2 μm (inset). **(I)** Quantification of Manders’ colocalization coefficient of CI-M6PR with Giantin, Rab14, or Rab11a in HeLa cells treated with the indicated siRNAs is shown from three independent experiments (*n* = 3). Colors in the SuperPlots indicate individual experiments, with each dot representing a single cell. The mean value for each experiment is indicated by a larger dot. Statistical significance was calculated using unpaired Student’s *t* test (**P = 0.0029 (Giantin); **P = 0.0014 [Rab14]; ***P = 0.0001 [Rab11a]). The values are represented as the mean ± SEM. CTCF, corrected total cell fluorescence. Source data are available for this figure: SourceData F8.

Since we found that TBC1D9B regulates the delivery of lysosomal cargo to their functional location, we next aimed to investigate how its depletion impacts lysosome morphology and function in cargo degradation. In TBC1D9B-depleted cells, LAMP1-positive punctae were more numerous, with an obvious depletion of the perinuclear pool and accumulation at the cell periphery (Fig. 8, A and B). This altered LAMP1 distribution was rescued in cells expressing siRNA-resistant TBC1D9B (WT) but not the GAP-deficient (TBC1D9B [RYQ→A]) and the Arl8b binding–defective mutant (TBC1D9B [E91A]), indicating that TBC1D9B binding to Arl8b and its function as a Rab GAP are required for normal LAMP1 distribution (Fig. 8, A and B). Using cryo-immunogold electron microscopy (EM), we observed that the average diameter of the LAMP1-positive vesicles was reduced upon TBC1D9B depletion (Fig. S5, E and F).

Next, to investigate how TBC1D9B depletion impacts the dynamics of lysosome distribution and motility, we performed live-cell imaging of dextran-loaded control and TBC1D9B-depleted cells. Analysis of the number, area, distribution, and motility of dextran-loaded terminal lysosomes showed an increase in the total lysosome number and mean area of peripheral lysosomes in TBC1D9B-depleted cells, whereas the mean area of perinuclear lysosomes was modestly reduced (Fig. S5, G and H; and Video 9). TBC1D9B-depleted cells also showed a decrease in lysosome clustering throughout the length scale analyzed, as reflected by Ripley’s K function analysis of dextran-loaded compartments (Fig. S5 I and Video 9). We also observed a decrease in the diffusion exponent of the dextran-loaded lysosomes upon TBC1D9B depletion in the cell periphery, suggesting a reduced directional motility of these compartments (Fig. S5 J and Video 9). These changes in the dextran-loaded lysosome behavior suggest an altered compartment identity of these vesicles upon TBC1D9B depletion.

**Video 9.** **Time-lapse imaging of control and TBC1D9B siRNA-treated HeLa cells incubated with Alexa Fluor 568–conjugated dextran overnight, followed by a 6-h chase to label terminal lysosomes.** The control and TBC1D9B siRNA videos are captured at 4.43 frames/sec with no time interval between the frames. The movies are shown at 4 frames/sec, and the total number of frames displayed is 100.

Previously, it has been shown that peripheral lysosomes are less accessible to biosynthetic cargo, and the margination of lysosomes to the cell periphery results in reduced cathepsin activity (Johnson et al., 2016). We next analyzed the degradative potential of lysosomes in TBC1D9B-depleted cells by analyzing active cathepsin levels. We found a significant decrease in cathepsin D total cell fluorescence and a significantly reduced colocalization of LAMP1 and cathepsin D upon TBC1D9B depletion (Fig. 8, C–F). The expression of the siRNA-resistant TBC1D9B (WT) but not its GAP-deficient (RYQ→A) and the Arl8b binding–defective mutant (E91A) rescued cathepsin D fluorescence, indicating that cathepsin D levels are regulated by TBC1D9B, and is dependent upon its GAP activity and binding to Arl8b (Fig. 8, C and D). Immunoblotting of cell lysates revealed a defect in cathepsin processing upon TBC1D9B depletion, as evident by reduced levels of mature cathepsin D and increased secretion of the pro-cathepsin D form in the extracellular media (Fig. 8 G).

In a previous study, we had identified that Arl8b-binding partner, RUFY1, regulates the endosomal retrieval of CI-M6PR from the Rab14-positive early/REs toward the TGN where it can mediate sorting of mannose-6-phosphate–tagged hydrolases to lysosomes (Rawat et al., 2023). As Rab14 and Rab11a have an overlapping localization on REs and share common effectors/partners, this led us to hypothesize that disruption of the Rab11a GTPase cycle in TBC1D9B-depleted cells could also affect cargo trafficking from the Rab14 compartment (Corteggio et al., 2022, *Preprint*; Kelly et al., 2009). Indeed, TBC1D9B-depleted cells showed increased retention of CI-M6PR in Rab14- and Rab11a-positive REs, while its overlap with Golgi was significantly reduced (Fig. 8, H and I). Notably, we found that TBC1D9B did not show GAP activity against Rab14, while its known GTP hydrolysis activity against Rab11a was observed in this assay (Fig. S5 K). Rab5 was used as a control in the *in vitro* GAP assay (Fig. S5 K). These observations suggest that TBC1D9B role as a Rab11a GAP is required for the efficient retrieval of CI-M6PR from Rab11a/Rab14-positive REs to the TGN.

**Figure 9.**
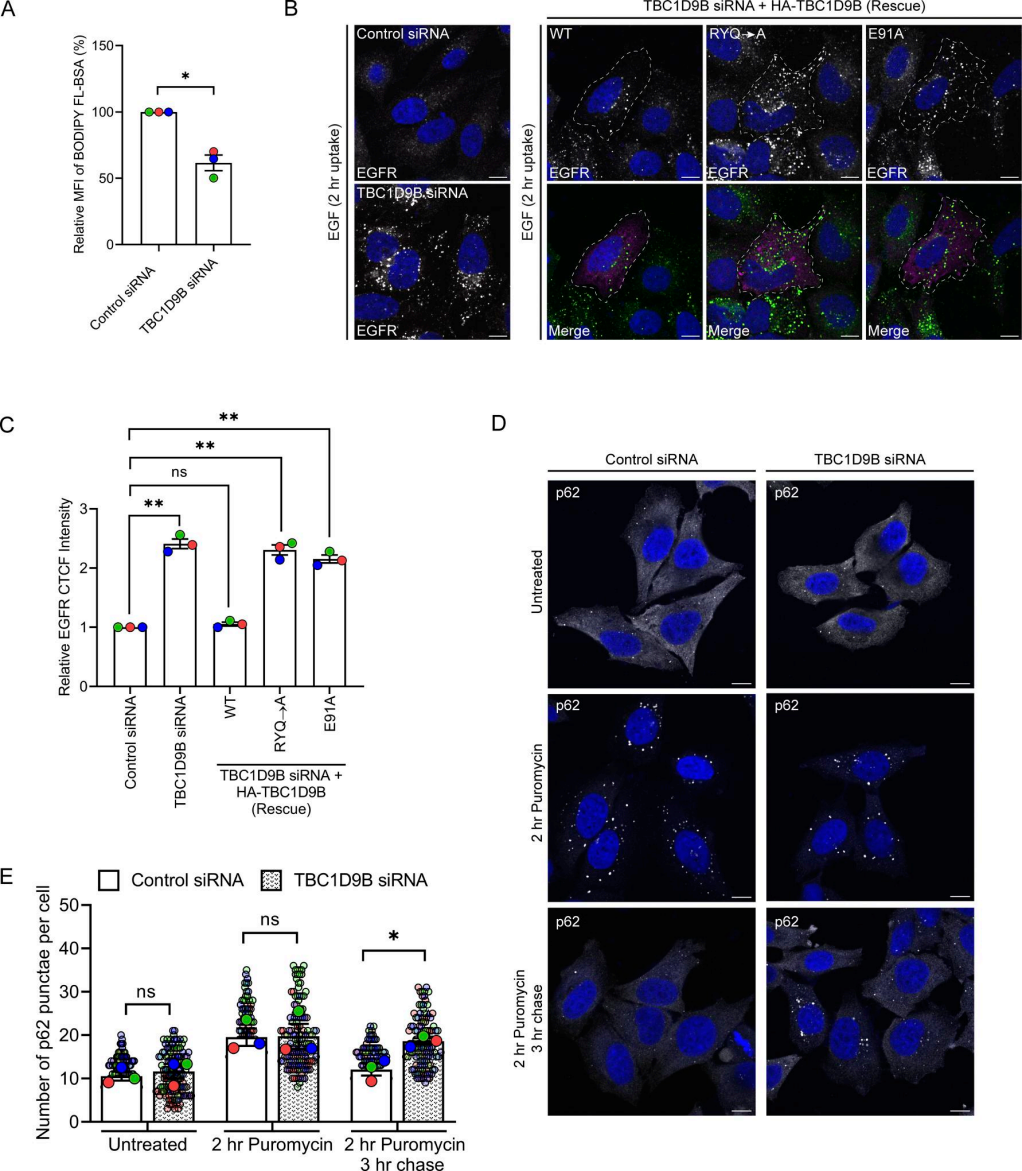
**Lysosomal cargo degradation is impaired upon TBC1D9B depletion. (A)** Graph represents the percent MFI of dequenched BODIPY FL-BSA in HeLa cells treated with control or TBC1D9B siRNA (*n* = 3; each dot represents a single experiment). Statistical significance was calculated using the one-sample *t* test (*P = 0.0231). The values are represented as the mean ± SEM. **(B)** Confocal micrographs of HeLa cells treated with the indicated siRNAs and transfected with the HA-TBC1D9B (WT, RYQ→A, or E91A rescue) construct, followed by EGF (100 ng/ml) uptake for 2 h. Cells were fixed and immunostained with anti-EGFR (green) and anti-HA (magenta) antibodies. Scale bar: 10 µm. **(C)** Graph shows quantification of CTCF of EGFR normalized to control siRNA (*n* = 3; each dot represents a single experiment). Statistical significance was calculated using the one-sample *t* test (**P = 0.0033 [TBC1D9B siRNA]; **P = 0.0042 [RYQ→A]; **P = 0.0034 [E91A]; n.s., non-significant). The values are represented as the mean ± SEM. **(D)** HeLa cells treated with control or TBC1D9B siRNA were incubated with puromycin (3 μg/ml) for 2 h, followed by a 3-h chase in complete media. Cells were fixed and immunostained with an anti-p62 antibody. Confocal micrographs of the indicated conditions are shown. Scale bar: 10 µm. **(E)** Quantification of the number of p62-positive punctae per cell for each condition in control or TBC1D9B siRNA is shown from three independent experiments (*n* = 3). Colors in the SuperPlots indicate individual experiments, with each dot representing a single cell. The mean value for each experiment is indicated by a larger dot. Statistical significance was calculated using unpaired Student’s *t* test (*P = 0.0143; n.s., non-significant). The values are represented as the mean ± SEM.

Finally, we assessed lysosomal cargo degradation in TBC1D9B-depleted cells. As compared to the control, TBC1D9B-depleted cells showed a ∼40% reduction in fluorescence intensity of BODIPY FL-BSA, an endocytic probe that fluoresces upon its proteolytic cleavage in lysosomes (Fig. 9 A). We next monitored the ligand-induced degradation of EGFR in control and TBC1D9B-depleted cells. There was a markedly reduced degradation of EGFR in TBC1D9B-depleted cells as compared to the control, which was rescued upon overexpression of siRNA-resistant TBC1D9B (WT) but not the GAP-deficient (RYQ→A) and the Arl8b binding–defective mutant (E91A) (Fig. 9, B and C). Next, we investigated autophagic clearance of puromycin-induced protein aggregates in control and TBC1D9B-depleted cells. After 2 h of puromycin treatment, both control and TBC1D9B siRNA-treated cells show protein aggregates marked with p62 (Fig. 9, D and E). To assess aggregate clearance, puromycin was washed off, and p62 punctae were measured after a 3-h chase in complete media (Fig. 9, D and E). Reduced clearance of p62 punctae was observed in TBC1D9B knockdown, suggesting a defect in clearance of autophagic cargo (Fig. 9, D and E). Taken together, these findings suggest that Arl8b and its effector TBC1D9B mediate Rab11a inactivation to prevent the recycling of newly synthesized lysosomal cargo LAMP1 and mediate its efficient sorting to promote lysosome biogenesis.

## Discussion

Arl8b was characterized as the first small GTP-binding protein on lysosomes based on its extensive colocalization with lysosomal glycoproteins, LAMP2 and CD63 (Bagshaw et al., 2006; Hofmann and Munro, 2006). The localization was found to be conserved across evolution, with a similar localization reported for the fly and worm orthologs of Arl8b (Hofmann and Munro, 2006; Nakae et al., 2010). Arl8b recruits its effectors to mediate lysosome fusion with other membranes and motility on microtubule tracks (Hofmann and Munro, 2006; Keren-Kaplan et al., 2022; Khatter et al., 2015a; Khatter et al., 2015b; Kumar et al., 2022; Kumar et al., 2024; Marwaha et al., 2017; Rosa-Ferreira and Munro, 2011; Walia et al., 2026). The mechanisms regulating Arl8b lysosomal localization, including the identity of its guanine nucleotide exchange factor (GEF) and GAP, are not well known. Notably, the BORC has been shown to interact with and regulate Arl8b lysosomal localization, although the underlying mechanism remains to be determined (Pu et al., 2015).

Besides its lysosomal distribution, Arl8b also localizes to vesicles positive for Rab14, which mark a subset of early/REs that harbor cargo for recycling to the plasma membrane, such as TfR, or cargo for retrieval to the TGN, such as CI-M6PR (Rawat et al., 2023; Yamamoto et al., 2010). Arl8b interacts with the Rab14 effector RUFY1 and mediates its stable membrane localization, which is required for CI-M6PR retrieval from endosomes to the TGN (Rawat et al., 2023). Corroborating that Arl8b and its interaction partners do localize to early endosomes, a recent study has shown that Arl8b, RUFY proteins (RUFY1, RUFY2, and RUFY3), Rab14, subunits of BORC, and LAMP1 are found in membrane fractions isolated by endo-IP of EEA1 (Park et al., 2022). Furthermore, an unbiased approach of crosslinking and native gel mass spectrometry of purified early endosomes revealed RUFY2 as a potential Arl8b interaction partner (Gonzalez-Lozano et al., 2025). Excitingly, a recent preprint has also shown that Rab14 GEF, DENND6A, has GTP hydrolysis activity toward Arl8b, suggesting an Arl8b-to-Rab14 transition might also be at play at this sorting compartment (Vignogna and Fromme, 2026, *Preprint*).

**Figure 10.**
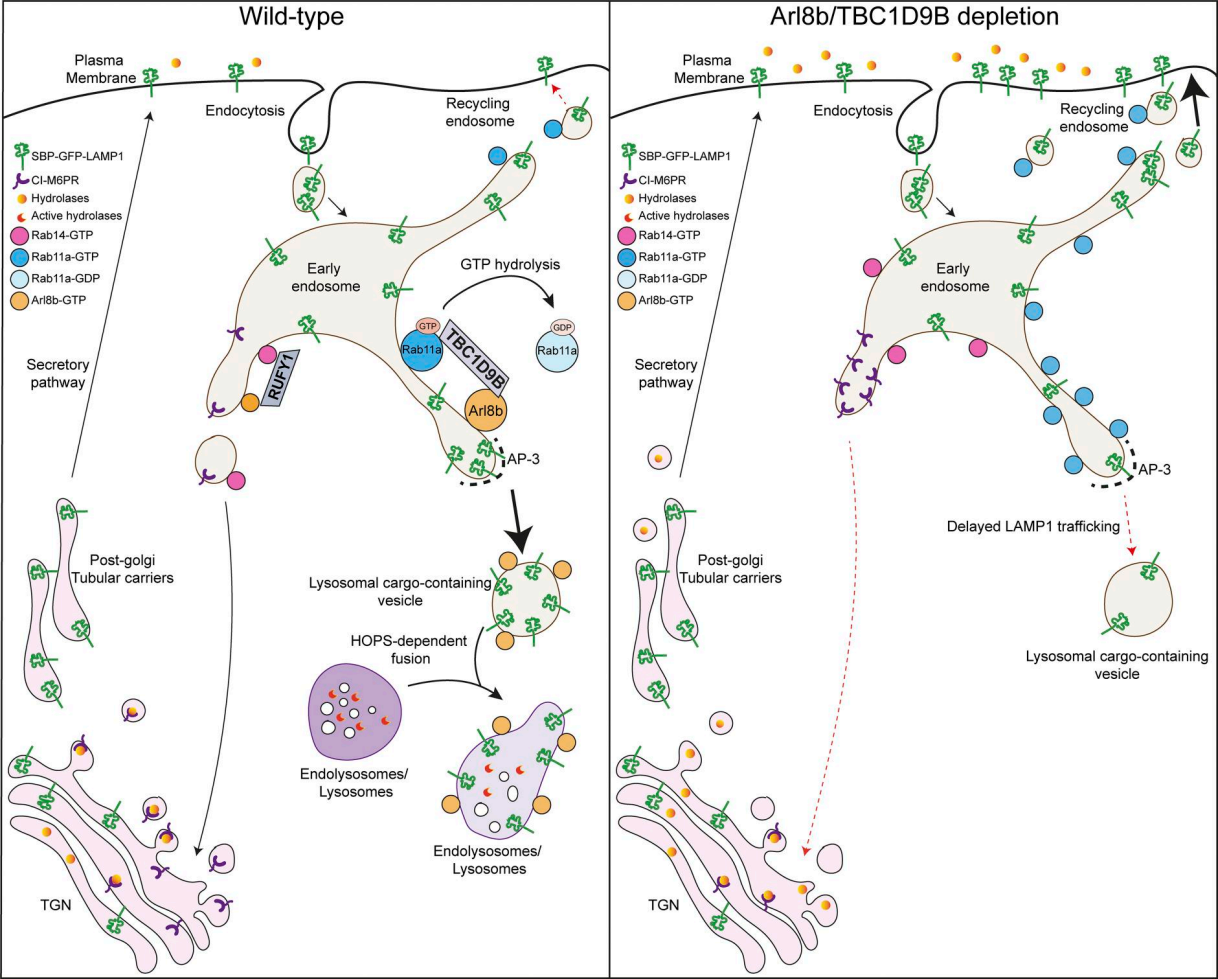
**Proposed role of Arl8b and its effector, TBC1D9B, in trafficking of the newly synthesized LAMP1 to active lysosomes.** Delivery of newly synthesized LAMP1 to active lysosomes follows an indirect trafficking pathway, wherein it exits the TGN in tubular carriers, followed by delivery to the plasma membrane. After endocytosis, LAMP1 is sorted from the AP-3–positive compartment in an Arl8b-dependent manner. Arl8b recruits the Rab11a GAP, TBC1D9B, which inactivates Rab11a to prevent recycling of LAMP1 to the cell surface and facilitates HOPS-mediated fusion of LAMP1-positive vesicles with pre-existing active lysosomes. Upon TBC1D9B depletion, Rab11a levels are increased on the newly synthesized LAMP1 vesicles, leading to recycling of LAMP1 to the cell surface.

The endosomes marked by RUFY1 and Rab14 represent an endocytic sorting station for TGN-bound cargo (Rawat et al., 2023), and also, since RUFY1 interacts with AP-3 (Ivan et al., 2012), it likely represents the sorting station for lysosomal glycoproteins such as LAMPs and CD63. Taking these findings into account, we hypothesized that Arl8b and a subset of its interaction partners regulate lysosome biogenesis. In this study, we report that Arl8b is a decision-maker for LAMP1 sorting, whereby the presence of Arl8b on newly synthesized LAMP1 endocytic vesicles suppresses their Rab11a-mediated recycling to the cell surface (Fig. 10). Indeed, both transiently expressed RUSH-LAMP1 and endogenous LAMP1 showed increased localization to REs upon Arl8b depletion, leading to an increase in surface LAMP1. Although this study focused on Arl8b′s role in LAMP1 sorting, it is likely that other lysosomal transmembrane proteins traffic together with LAMP1 on the same cargo vesicle. It will be relevant in future studies to employ high-throughput approaches to identify the lysosomal cargoes that are missorted upon Arl8b depletion.

We found that Arl8b is recruited to newly synthesized LAMP1-containing endocytic vesicles before their fusion with active lysosomes and, in turn, recruits the Rab11a GAP, TBC1D9B, on these non-acidic LAMP1-positive vesicles. Interestingly, TBC1D9B localized to a subset of Arl8b- and LAMP1-positive vesicles that were generally more peripheral, and its localization was not observed on the perinuclear acidic and degradative lysosomes. TBC1D9 paralogs contain GRAM domains that are known to recognize cholesterol and phosphoinositides; thus, it is possible that besides Arl8b, coincident detection of lipids is required for TBC1D9B membrane localization (Doerks et al., 2000; Ercan et al., 2021; Tsujita et al., 2004). Furthermore, as the colocalization of the N-terminal fragment of TBC1D9B (1–270 aa) containing the first GRAM domain with Arl8b was better than the full-length protein, this suggests a regulatory role of its other domains, including the TBC domain and the EF-hand motifs, in determining Arl8b binding and recruitment to membranes. Although TBC domain–containing proteins are characterized only as Rab GAPs (Fukuda, 2011), whether TBC1D9B also has GAP activity toward Arl8b was not clear. While this manuscript was under communication, a study has shown that TBC1D9B facilitates *in vitro* GTP hydrolysis activity against Arl8b (Duhay et al., 2026). It is plausible that once recruited on the LAMP1-positive vesicles, TBC1D9B has a second function to regulate the Arl8b GTPase cycle. Our unpublished preliminary observations also indicated that TBC1D9B, but not its GAP-defective mutant, had a weaker GAP activity toward Arl8b as compared to Rab11a.

Previously, we had shown RUFY1 is a dynein adaptor that mediates CI-M6PR retrieval from Rab14-positive endosomes to the TGN (Rawat et al., 2023). Taking into account our current work that suggests that newly synthesized LAMP1 also traffics likely via a RUFY1-positive endosome, we hypothesize that lysosomal hydrolases and glycoproteins likely co-traffic from this subcellular location, eventually forming hydrolase-containing storage lysosomes, while CI-M6PR is retrieved back to the TGN. Interestingly, a recent study has shown that an intrinsically disordered region within RUFY1 aids in the formation of a proteinaceous liquid-like matrix in mouse oocytes to form endolysosomal vesicular assemblies that harbor endolysosomes, autophagosomes, and proteasomes (Zaffagnini et al., 2024). It will be interesting and relevant to determine whether lysosome biogenesis similarly involves the assembly of lysosomal glycoproteins and hydrolases in the liquid-like proteinaceous matrix of RUFY proteins. TBC1D9B-mediated Rab11a-to-Arl8b handover would be a crucial step for preventing recycling of lysosomal cargo at this sorting and packaging station and also for CI-M6PR retrieval back to the TGN.

In summary, by governing the membrane association of its interaction partners, including RUFY1 (and potentially RUFY2), TBC1D9B, and the HOPS complex, Arl8b could serve as a master regulator for the delivery of lysosomal cargoes and lysosome function. Future studies employing the RUSH assay to study co-trafficking of newly synthesized hydrolases and lysosomal transmembrane proteins together could help in clarifying the model for lysosome biogenesis.

## Materials and methods

### Cell culture

HeLa and HEK293T cells were cultured in DMEM (Gibco) supplemented with 10% FBS (Gibco) at 37°C with 5% CO_2_ in a humidified cell culture chamber. RPE-1 cells were maintained in DMEM/F-12 (Gibco) media with 10% FBS and 0.01 mg/ml hygromycin (Invitrogen), while THP-1 cells were cultured in RPMI 1640 (Gibco) media with 10% FBS at 37°C with 5% CO_2_ in a humidified incubator. THP-1 monocytes were differentiated to macrophages by treatment with 30 ng/ml of phorbol 12-myristate 13-acetate (PMA; Sigma-Aldrich) for 24 h, followed by a resting period of 48 h in PMA-free medium. For live-cell imaging and flow cytometry, cells were incubated in phenol red-free DMEM (Gibco) supplemented with FBS as described above. All cell lines were routinely tested for *Mycoplasma* using MycoAlert *Mycoplasma* Detection Kit (Lonza), and cultures were limited to 18 passages. Arl8b KO HeLa cells were generated using CRISPR/Cas9 with the sgRNA target sequence: 5′-GAT​GGA​GCT​GAC​GCT​CG-3′, as previously described (Marwaha et al., 2017).

### Generation of Arl8b^EN^-mStayGold KI HeLa cells

To engineer HeLa cells expressing endogenous Arl8b in frame with fluorescent protein mStayGold (Arl8b^EN^-mStayGold), we used a previously described protocol (Stockhammer et al., 2024). Briefly, the guide RNA (gRNA) targeting the Arl8b gene locus was designed using the Benchling platform (https://www.benchling.com) and cloned into the SpCas9 pX330 (plasmid #42230; Addgene) vector (Cong et al., 2013). The gRNA sequences used to target the Arl8b locus were as follows: 5′-CAC​CGA​AGA​AGG​ACT​GGA​AGA​CTT​C-3′ and 5′-AAA​CGA​AGT​CTT​CCA​GTC​CTT​CTT​C-3′. The oligonucleotides were annealed and ligated into the vector linearized using the Bbs1 restriction enzyme.

For generating the homologous recombination (HR) donor plasmid, 1-kb homology arms were synthesized commercially (Twist Biosciences). A glycine–serine linker and a 5′ BamH1 and 3′ EcoR1 restriction enzyme sites were added between two homology arms for the insertion of a tag and resistance cassette. The mStayGold-PolyA-Hygromycin cassette was excised from AP1muA-mStayGold-PolyA-Hygromycin HR (plasmid #229679; Addgene) (Cong et al., 2013) and transferred to generate the final HR donor plasmid. HeLa cells were transiently co-transfected with 1 µg of gRNA and final HR donor plasmids using X-tremeGENE HP DNA transfection reagent (Roche). After 24 h of transfection, cells were selected by the addition of 300 µg/ml hygromycin for 72 h. The cells that survived the hygromycin selection were sorted based on the mStayGold signal using the BD FACSAria Fusion cytometer.

### Gene silencing

For gene silencing experiments, siRNA oligonucleotides were purchased from Dharmacon and prepared according to the manufacturer’s protocol. Transient transfection with 100 nM siRNA was carried out using DharmaFECT1 reagent (Dharmacon) for 65–72 h. The following siRNAs were used in this study: control siRNA, 5′-TGG​TTT​ACA​TGT​CGA​CTA​A-3′; human Arl8b siRNA, 5′-AGG​TAA​CGT​CAC​AAT​AAA​GAT-3′; human Vps33a siRNA, 5′-GGGAGGAGTACAG CTTAGATCTC-3′; ON-TARGETplus human AP-3 siRNA SMARTpool (L-016014); TBC1D9B siRNA, 5′-CAG​GAA​CAT​CTC​AGC​CCT​GAA-3′; and human Rab11a siRNA SMARTpool (L-004726).

### Plasmids, antibodies, and chemicals

All the plasmids and antibodies used in this study are listed in Tables S3 and S4, respectively. Most of the chemicals used in this study were purchased from Sigma-Aldrich. LTR Deep Red, epidermal growth factor (EGF), dextran (Alexa Fluor 568–conjugated dextran; red), DAPI, and hygromycin were purchased from Invitrogen. SiR-Lysosome was purchased from Cytoskeleton, and Halo ligands were purchased from Promega. The pitstop2 inhibitor was purchased from Abcam, and ionomycin was purchased from Millipore. Biotin, PMA, and puromycin were purchased from Sigma-Aldrich, while BODIPY FL-BSA was purchased from BioVision.

### Transfection, immunofluorescence staining, and live-cell imaging

For transfection, cells cultured on glass coverslips (VWR) were transfected with the desired DNA constructs using the X-tremeGENE HP DNA transfection reagent (Roche) for 16–20 h. For fixed-cell experiments, cells were fixed using 4% paraformaldehyde (PFA) in PHEM buffer (60 mM PIPES, 10 mM EGTA, 25 mM HEPES, 2 mM MgCl_2_, and final pH 6.8) for 10 min at room temperature (RT). For staining using rabbit anti-cathepsin D antibody (Abcam), cells were fixed using ice-cold methanol (Sigma-Aldrich) for 10 min at −20°C. For immunostaining after fixation, cells were first incubated with blocking solution (0.2% saponin plus 5% FBS prepared in PHEM buffer) for 30 min at RT. Coverslips were then washed three times with 1X PBS and proceeded with incubation with primary antibody in staining solution (0.2% saponin plus 5% FBS prepared in PHEM buffer) for 2 h at RT. Cells were again washed three times with 1X PBS before proceeding with secondary antibody staining for 1 h at RT using Alexa fluorophore-conjugated secondary antibodies prepared in staining solution. Following secondary antibody staining, coverslips were washed with 1X PBS three times and mounted on glass slides (HiMedia) using Fluoromount G (Southern Biotech). For imaging fixed cells, confocal images were acquired using Carl Zeiss 710 Confocal Laser Scanning Microscope with a Plan Apochromat 63×/1.4 NA oil immersion objective with ZEN 2012 v. 8.0.1.273 (Carl Zeiss) software for image acquisition. To ensure little to no pixel saturation during image capturing, optimized exposure and detector gain settings were used. Representative images were uniformly adjusted for brightness and contrast using Fiji software or Adobe Photoshop CS 2024.

For labeling Halo-tagged proteins in fixed or live-cell settings, cells were incubated with Halo ligands (Promega), either HaloTag TMR Ligand (71.4 nM) or Janelia Fluor 646 HaloTag Ligand (20 nM), for 30 min in phenol red–free complete DMEM containing 10% FBS. For fixed-cell imaging experiments, cells were then washed and incubated in fresh DMEM for 30 min and then fixed with 4% PFA.

Lysosome labeling in live-cell imaging experiments with LTR (Invitrogen) or SiR-Lysosome (Cytoskeleton, Inc.) probes was performed using the manufacturer’s instructions. Briefly, cells were incubated in phenol red–free complete DMEM supplemented with 10% FBS containing SiR-Lysosome (1 µM) or LTR Deep Red (100 nM) for 15 min at 37°C in a cell culture incubator before proceeding for imaging.

For live-cell imaging experiments, cells were cultured on glass-bottom live-cell dishes (ibidi). For transfection, a protocol similar to fixed-cell imaging experiments was followed using the desired plasmids. Cells were incubated in phenol red–free complete DMEM containing 10% FBS before proceeding with live-cell imaging to minimize background fluorescence. Live-cell imaging was performed using Olympus IXplore SpinSR Spinning Disk Super-Resolution Confocal Microscope using a 100× Apo/1.45 NA oil objective equipped with an environmental chamber set at 37°C and 5% CO_2_. The images were acquired using Olympus CellSens software.

### Image analysis and quantification

#### Colocalization analysis

For colocalization analysis, images were processed using Just Another Colocalization (JaCoP) Plugin in ImageJ. After splitting the channels and applying manual thresholding to determine appropriate intensity cutoffs, we quantified colocalization using either Pearson’s correlation coefficient (PCC) or Manders’ overlap coefficients (M1 and M2), as appropriate for each experiment. PCC measures the linear correlation between signal intensities in two channels, indicating both spatial overlap and proportionality of intensities, and is most suitable when the two molecules are expected to directly interact or covary in concentration. In contrast, Manders’ coefficients quantify the fraction of one signal overlapping with another, regardless of intensity correlation, and are more appropriate when assessing spatial co-occurrence between markers that do not directly interact. The rationale for the choice of metric in each experiment is clarified below.

PCC is a measure of linear relationship between signal intensities, while Manders’ coefficients assess co-occurrence by measuring the proportion of signal overlap (i.e., spatial colocalization irrespective of intensity proportionality). High PCC indicates that the two molecules are present in the same structure and their concentrations are correlated, while high Manders’ value only suggests that the two molecules occupy the same spatial region.

To assess the delivery of newly synthesized RUSH-LAMP1 to distinct cellular compartments (such as early or REs or active lysosomes), we quantified signal overlap using Manders’ colocalization coefficient. This metric was chosen because the compartment-specific markers (like Rab11a to mark REs or LTR to mark active lysosomes) used in these experiments do not directly interact with RUSH-LAMP1 but serve solely as indicators of cellular localization (Fig. 1 G; Fig. 2, B, D, and E; Fig. 3 B; Fig. 4, B, D, and F; Fig. 7, B and D; Fig. S1, J, L, N, and P; Fig. S2, C, E, and G; and Fig. S3, C and F). In contrast, colocalization of mCherry anti-GFP nanobody with RUSH-LAMP1 was quantified using PCC, as the nanobody directly binds the GFP tag on RUSH-LAMP1, and similarly, colocalization of GFP-TBC1D9B with Arl8b-Halo was quantified using PCC (Fig. 1 J and Fig. 6 B). Similarly, Manders’ colocalization coefficient is used in additional experiments assessing colocalization between markers that do not directly interact (Fig. 6, C, E, and F; Fig. 8 I; Fig. S3, H and K; and Fig. S5 A).

#### Intensity profile

To generate an intensity profile in ImageJ, images were converted to an RGB stack, a line was drawn across the region of interest (ROI), and the intensity profile was generated using the “RGB plot profile” tool.

#### Quantification of the punctum number

To quantify the p62 punctum number, Fiji software was used. Briefly, images were opened in the software, and a manual threshold was applied, followed by the calculation of the p62 punctum number using the “Analyze particles” option in Fiji under the “Analyze” menu.

#### Analysis of lysosome distribution

Fiji software was used to quantify the distribution of lysosomes in HeLa cells, ranging from 500 to 1400 µm^2^ based on LAMP1 signal intensity. The periphery of each cell was manually outlined using the freehand selection tool, and the signals of adjacent cells were excluded using the “Clear Outside” tool. A ROI was drawn around the nucleus, and the lysosomal signal within the region was measured. The ROI was then expanded by 5 µm, and LAMP1 intensity was measured within each zone. Perinuclear intensity (0–5 µm) was calculated by subtracting the signal of the nucleus from the next zone, while peripheral intensity was measured for distances >10 µm from the nucleus. These intensities were normalized to the total intensity of cells as *I*_< 5_ = *I*_perinuclear_/*I*_total_ and I_>10_ = *I*_periphery_/*I*_total_. The perinuclear index was calculated as (*I*_< 5_ – *I*_> 10_) X 100.

#### Quantification of corrected total cell fluorescence

The corrected total cell fluorescence (CTCF) values were calculated using the formula CTCF = integrated density - (area of the selected cell X mean fluorescence of background). Images were imported into ImageJ software, and the relevant parameters were measured using the “Measure” option under the Analyze menu.

### RUSH assay

For fixed-cell imaging experiments, HeLa cells were seeded on 12-mm glass coverslips (VWR) placed in a 35-mm culture plate 1 day before transfection. For live-cell imaging experiments, HeLa cells were seeded in a glass-bottom 35-mm live-cell imaging dish (ibidi) 1 day before transfection. For experiments involving gene knockdown using siRNA, cells seeded in either a 35-mm dish or glass-bottom live-cell imaging dish were treated with indicated siRNAs for 50 h, followed by transfection with the RUSH reporter construct Str-KDEL-IRES-SBP-GFP-LAMP1.

For fixed-cell imaging experiments, the 20-h post-transfection media of the cells were replaced with complete media containing biotin (Sigma-Aldrich) at a concentration of 40 µM for different time periods. At the end of the indicated time point after biotin addition, cells were fixed using 4% PFA (prepared in PHEM buffer) for 10 min at RT. Cells were immunostained for the indicated antibodies as described above, and single-plane confocal images were acquired on an LSM 710 confocal microscope using a 63×/1.4 NA oil immersion objective and Zen Black 2012 software (Carl Zeiss).

For live-cell imaging experiments, the 20-h post-transfection media of the cells were replaced with phenol red–free DMEM containing 10% FBS and biotin (40 µM), and confocal imaging of cells was performed at 37°C using Olympus IXplore SpinSR Spinning Disk Super-Resolution Confocal Microscope using a 100× Apo/1.45 NA oil objective and CellSens software.

### mCherry-tagged anti-GFP nanobody uptake assay

HeLa cells were seeded on 12-mm glass coverslips or glass-bottom live-cell imaging dishes for fixed- or live-cell imaging experiments, respectively. Cells were transfected with the RUSH reporter construct Str-KDEL-IRES-SBP-GFP-LAMP1 using X-tremeGENE HP. For fixed-cell imaging experiments, the 20-h post-transfection media of cells were replaced with complete DMEM containing biotin (40 µM) and supplemented with recombinantly purified mCherry-tagged anti-GFP nanobody (25 µg/ml), and this interval was considered as time 0. Cells were fixed using 4% PFA (prepared in PHEM buffer) for 10 min at RT for the indicated time points after biotin addition. The glass coverslips were then mounted on glass slides, and single-plane confocal images were acquired on an LSM 710 confocal microscope using a 63×/1.4 NA oil immersion objective and Zen Black 2012 software (Carl Zeiss).

### LAMP1 recycling assay

HeLa cells seeded on 12-mm glass coverslips (VWR) were treated with designated siRNAs for 48 h, followed by transfection with the RUSH reporter construct Str-KDEL-IRES-SBP-GFP-LAMP1 for 18 h. To assess the recycling of biosynthetic LAMP1, media containing primary rabbit anti-GFP antibody (dilution 1:1,000) and biotin (40 µM) were added to the cells and incubated for 30 min at 37°C to allow internalization of surface-labeled antibody-bound GFP-LAMP1. After incubation, the unbound and surface-associated antibody was removed by washing the cells three times with citric acid buffer containing 0.1 M citric acid anhydrous and 0.1 M trisodium citrate dihydrate (pH 4.5) for 3 min on ice. To allow recycling of surface internalized antibody–bound GFP-LAMP1, cells were incubated at 37°C for an additional 30 min in fresh complete media containing 30 µM pitstop2 inhibitor (Abcam). Following the recycling period, cells were fixed with 2.5% PFA prepared in 1X PBS for 15 min on ice. To label surface-recycled GFP-LAMP1, fixed cells were stained under non-permeabilized conditions using Alexa Fluor 568–conjugated secondary goat anti-rabbit antibody (dilution 1:500) prepared in PHEM buffer for 30 min at RT. The coverslips were mounted on glass slides and imaged using confocal microscope. To quantify the LAMP1 recycling to the plasma membrane, the CTCF values for both the surface LAMP1 (Alexa Fluor 568 signal) and total GFP-LAMP1 were measured using Fiji software as described previously, and the average ratio was calculated and plotted.

### Analysis of LAMP1 trafficking from the TGN to the plasma membrane

HeLa cells seeded on glass coverslips were treated with control or Arl8b siRNA for 48 h, followed by transfection with the RUSH construct Str-KDEL-IRES-SBP-GFP-LAMP1 for 18 h. To determine the trafficking of LAMP1 from the TGN to the plasma membrane, cells were incubated in complete DMEM containing rabbit anti-GFP primary antibody (dilution 1:1,000) and supplemented with biotin (40 μM) and pitstop2 (30 µM) for 30 min. Following incubation, coverslips were fixed with 2.5% PFA prepared in 1X PBS for 15 min on ice. The fixed coverslips were stained with an Alexa Fluor 568–conjugated goat anti-rabbit secondary antibody (diluted in PHEM buffer containing 5% FBS) for 30 min. The coverslips were mounted and imaged by confocal microscope as described above.

### Dextran uptake assay and analysis of dextran-loaded compartments

To perform dextran uptake, HeLa cells seeded in a glass-bottom live-cell imaging dish were treated with control or TBC1D9B siRNA. After 50 h of siRNA treatment, cells were incubated with Alexa Fluor 568–conjugated dextran (20 µg/ml) for 16 h. At the end of the incubation period, cells were washed with 1X PBS, and fresh phenol red–free complete DMEM were added, and the internalized dextran was chased for 6 h before proceeding for live-cell imaging in an environmental chamber set at 37°C and 5% CO_2_ using Olympus IXplore SpinSR Spinning Disk Super-Resolution Confocal Microscope using a 100× Apo/1.45 NA oil objective. The different analysis of dextran-loaded compartments in control or TBC1D9B siRNA-treated cells was done as described below.

#### Mean lysosome number analysis

Live-cell imaging videos of HeLa cells treated with designated siRNAs and incubated with Alexa Fluor–conjugated dextran to label lysosomes were analyzed using a custom Python pipeline. Lysosomes were first identified as individual objects within each frame of the time-lapse series. After analyzing all the frames for a given sample, a Python script was used to compute the mean values.

#### Area analysis

Live-cell imaging videos of HeLa cells treated with designated siRNAs and incubated with Alexa Fluor–conjugated dextran to label lysosomes were analyzed using a custom Python pipeline. Individual video frames were extracted, converted to grayscale, and preprocessed to suppress background noise. Below, we provide a detailed description of the process to quantify lysosomal area within defined subcellular regions.

We began by generating binary masks for the peripheral cytoplasm and the perinuclear region of the HeLa cell. The peripheral region was defined as the cytoplasmic band adjacent to the cell boundary, with a fixed thickness of 60 pixels (or 7.8 µm). To construct this region, the outermost distribution of lysosomes in the first frame was used as a proxy for the cell outline. The centroids of all detected lysosomes were subjected to the Delaunay triangulation (Ito, 2015), and the boundary was approximated using the alpha shape algorithm with an empirical α value of 0.02, which generates a concave hull that tightly follows the geometry of the cell periphery (Edelsbrunner et al., 2003). From this boundary, a binary mask was created and morphologically expanded inward by 60 pixels to yield a rim-shaped mask corresponding to the peripheral cytoplasm. The perinuclear region was defined as an outward concentric band surrounding the nucleus, with a fixed thickness of 40 pixels (5.2 µm). To delineate this region, the nuclear boundary was manually annotated in the second frame of each video. The resulting contour was converted into a binary mask and expanded outward through iterative morphological dilations. Subtraction of the original nuclear mask from the dilated mask led to a uniform annular region that captured the immediate cytoplasmic environment adjacent to the nucleus. For both regions, the corresponding masks were applied to the original grayscale images using bitwise logical operations. Lysosomes confined within each compartment were extracted, binarized via global thresholding (intensity threshold = 100), and segmented into connected components using the contour detection function. The projected 2D areas of the segmented lysosomes were measured using the OpenCV (https://github.com/opencv/opencv/wiki/CiteOpenCV) function, and the average lysosomal area per frame was computed separately for the peripheral and perinuclear compartments. Repeating this process across all frames resulted in compartment-specific lysosomal area distributions for subsequent statistical analysis.

#### Ripley’s K function analysis

Ripley’s K function analysis is a statistical method to quantify the degree of spatial clustering or dispersion as a function of distance (Dixon, 2002; Ripley, 1977). It quantifies how many neighboring points are found within a given radius “*r*” of each point in the dataset and compares this count with what would be expected under complete spatial randomness (CSR). If the observed value is greater than the theoretical value under CSR (which is πr^2^ in 2D), the pattern is considered clustered at that scale; if it is lower, the pattern is considered dispersed. Analysis of K(r) across a range of radii reveals whether clustering or dispersion exists and identifies the spatial scales at which these patterns are most pronounced.

We employed this methodology to quantify and compare the spatial distribution of lysosomes in HeLa cells treated either with control or with TBC1D9B siRNA using time-lapse data of our sample cells. From each video, the first 50 frames were extracted and converted to 8-bit grayscale. Lysosomal punctae were segmented using global intensity thresholding, and their centroids were extracted via contour detection and image moment calculations using OpenCV functions. These centroids formed the point pattern for each frame. For each frame, pairwise Euclidean distances between centroids were computed, and K(r) was calculated across a range of radii (1–250 pixels) using an area-normalized count of point pairs within each distance threshold. The theoretical K(r) under the CSR condition was also calculated. K(r) curves from all 50 frames of a video were averaged to find the mean distribution corresponding to that individual sample. Importantly, this frame selection did not compromise the temporal information, as the samples were imaged after sufficient resting time after perturbation, ensuring that a steady state had already been achieved. As a result, the extracted frames reliably captured the representative spatial organization of lysosomes without temporal bias.

To enable meaningful biological interpretation, we went beyond single-sample analyses and performed group-level averaging by aggregating these video-level distribution curves across 62 independent video samples from the control siRNA treatment group and 77 from TBC1D9B siRNA treatment group conditions. For each group, the mean K(r) and 95% confidence intervals were computed, providing a statistically robust representation of spatial organization trends within the population.

#### Diffusion analysis

To characterize lysosomal diffusion within different spatial regions of HeLa cells, we developed a custom image analysis pipeline that segments cellular regions, extracts lysosome coordinates, and tracks their motion using the open-source TrackPy library (https://zenodo.org/records/60550) (Crocker and Grier, 1996). We systematically studied lysosomal diffusion across the (1) cell, (2) the perinuclear region, and (3) the peripheral region.

We began by processing time-lapse fluorescence microscopy videos of individual HeLa cells, each containing lysosome-specific fluorescent signals. To carry out our analysis, we created spatial masks to segment the cytoplasm into three regions: the whole-cell region (entire visible cytoplasm), the perinuclear region, and the peripheral region, as described in the first section. Lysosome centroids from each region were tracked independently using TrackPy’s linking function in Python. This function reconstructs particle trajectories by linking detections across sequential frames. We used a maximum search range of nine pixels and a memory of five frames, allowing temporary disappearance of lysosomes without prematurely terminating their tracks. To ensure reliability, only trajectories with a minimum length of 20 frames were retained for analysis.

For each region, we computed the ensemble mean squared displacement (EMSD) as a function of lag time τ, which quantifies average lysosomal displacement over increasing time intervals. EMSD was defined as follows:

$$\text{EMSD}\left(\tau \right)=\langle {\left[x\left(t+\tau \right)‐x\left(t\right)\right]}^{2}+{\left[y\left(t+\tau \right)‐y\left(t\right)\right]}^{2}\rangle .$$

To assess the mode of the lysosomal motion, we fitted the EMSD curves to a power law of the form:

$$\text{EMSD}\left(\tau \right)=4D\tau^{\alpha }.$$

Here, D is the generalized (apparent) diffusion coefficient, and α is the diffusion exponent. Values of α <1 indicate subdiffusive behavior, α = 1 represents normal Brownian diffusion, and α >1 suggests superdiffusion, often due to directed transport. Fitting was performed in log–log space using linear regression over the EMSD vs. lag-time curve for each tracked region. The extracted diffusion exponents (α) were compiled for all videos and grouped by region. Statistical comparisons between the whole-cell, perinuclear, and peripheral compartments allowed us to identify spatially distinct patterns of the lysosomal motion.

### Flow cytometry

To perform quantitative analysis of surface levels of LAMP1, LAMP2, and EGFR, flow cytometry was performed. Briefly, cells were seeded in 12-well culture plates, followed by treatment with the indicated siRNAs. 60 h after siRNA treatment, cells were placed on ice and washed with ice-cold 1X PBS, and 1 ml of 0.5 mM EDTA (Sigma-Aldrich) prepared in 1X PBS was added to cells for 20 min. Cells were detached from the plate by gentle pipetting, followed by pelleting at 1600 × *g* for 2 min at 4°C. Cells were washed twice with ice-cold flow buffer (0.2% BSA prepared in 1X PBS) and resuspended in 100 μl of flow buffer. For determining the surface levels of LAMP1, an Alexa Fluor 647–conjugated mouse anti-LAMP1 antibody (dilution 1:20) prepared in flow buffer was added to cells for 50 min on ice. The cells were washed twice with flow buffer to ensure excess antibodies were washed away, and samples were acquired on a BD FACSAria Fusion cytometer, and data acquisition was performed using BD FACSDiva software (version 8.0.1; BD Biosciences). The data analysis was done using BD FlowJo version 10.0.1.

For quantifying surface levels of LAMP2, a similar staining protocol was performed using an Alexa Fluor 647–conjugated mouse anti-LAMP2 antibody (dilution 1:20). For determining surface levels of EGFR, cells were first stained with primary mouse anti-EGFR antibody (dilution 1:100) for 50 min on ice, followed by a subsequent incubation with an Alexa Fluor 488–conjugated secondary goat anti-mouse antibody (dilution 1:2,000) (prepared in flow buffer) for 40 min on ice. Both primary and secondary antibody incubations were followed by two washes with flow buffer, and then, the cells were acquired and analyzed as described above.

### β-Hexosaminidase assay

HeLa cells transfected with designated siRNAs were incubated for 10 min in Opti-MEM (Gibco) with or without 10 µM ionomycin (Millipore) and 4 mM CaCl_2_. The supernatant was collected, and cells were lysed in ice-cold TAP lysis buffer containing 20 mM Tris (pH 8.0), 150 mM NaCl, 0.5% NP-40, 1 mM MgCl_2_, 1 mM Na_3_VO_4_, 1 mM NaF, 1 mM PMSF, and protease inhibitor cocktail. The lysates were further diluted (1:5) in deionized water. β-Hexosaminidase activity was determined using the kit manufacturer’s protocol (MET-5095; Cell Biolabs, Inc.). Briefly, 50 μl of each sample was added to wells of a black 96-well microtiter plate (Thermo Fisher Scientific). Subsequently, 50 μl of 1X substrate solution was added and incubated at 37°C for 15 min, protected from light. Following incubation, 100 μl of 1X neutralization buffer was added to each well. Fluorescence was then measured using the microplate fluorometer (TECAN Infinite M PLEX) with excitation at 365 nm and emission at 450 nm. The protein content in both media and lysates was measured using a bicinchoninic acid (BCA) kit (Sigma-Aldrich). β-Hexosaminidase activity was calculated using the formula:

$$\beta ‐hex\;activity=\left(\text{fluorescence}\;{\text{readings}}_{365\;\text{nm}/450\;\text{nm}}\;–\;{\text{blank}}_{365\;\text{nm}/450\;\text{nm}}\right)\;\;/\;\text{protein}\;\left(µg\right)$$

$$Total\;\beta ‐hex\;activity\;=\left({\text{activity}}_{\text{supernatant}}\right)+\left({\text{activity}}_{\text{lysate}}\;X\;\text{dilution}\;\text{factor}\right)$$

$$\mathrm{Percentage}\;\beta ‐\mathrm{hex}\;\mathrm{released}\;=\left({\text{activity}}_{\text{supernatant}}\;/\;\text{total}\;\beta ‐\text{hex}\;\text{activity}\right)\;X\;100.$$

### Cathepsin D secretion assay

For measuring the secretion of mature cathepsin D into the extracellular culture media, HeLa cells were cultured in complete growth media and transfected with the desired siRNAs. After 48 h of siRNA treatment, the media were replaced with serum-reduced medium (Opti-MEM; Gibco), and cells were incubated for an additional 24 h. Following incubation, Opti-MEM was collected and centrifuged at 1,600 × *g* for 10 min to remove cell debris. The clarified supernatant was precipitated using 15 μl of concanavalin A resin (Sigma-Aldrich) for mature cathepsin D.

For estimating the secretion of pro-cathepsin D into the extracellular culture media, ice-cold acetone was added in a 1:4 ratio to the collected clarified Opti-MEM as described above, followed by incubation at −20°C for 4 h. After incubation, the media were centrifuged at 16,000 × *g* for 10 min, and the supernatant was removed. The resulting pellets were air-dried and resuspended in 1X PBS. Samples were boiled in 1X SDS sample loading buffer at 100°C for 10 min and further subjected to SDS-PAGE and immunoblotting as described previously.

### Whole-cell lysates and immunoblotting

For lysate preparation, cells were lysed in ice-cold RIPA buffer containing 10 mM Tris-Cl (pH 8.0), 1 mM EDTA, 0.5 mM EGTA, 1% Triton X-100, 0.1% SDS, 0.1% sodium deoxycholate, and 140 mM NaCl, supplemented with PhosSTOP (Roche), 1 mM PMSF (Sigma-Aldrich), and 1X protease inhibitor cocktail (Sigma-Aldrich). Lysates were incubated on ice for 2 min, followed by vortexing for 30 s. This cycle was repeated five times, and samples were then centrifuged at 16,627 × *g* for 10 min at 4°C. The resulting supernatants were collected, and protein concentrations were determined using the BCA kit (Sigma-Aldrich).

For immunoblotting, protein lysates were mixed with 4X Laemmli sample buffer, denatured by boiling, and resolved by SDS-PAGE. Proteins were transferred to PVDF membranes (Bio-Rad) and blocked overnight at 4°C in blocking buffer containing 10% skim milk (BD Difco) in 1X PBS with 0.05% Tween-20 (Sigma-Aldrich). After blocking, membranes were washed with 0.05% PBS/Tween buffer and incubated with primary antibodies diluted in the same buffer for 2 h at RT. The blots were then washed three times for 10 min each in 0.05% PBS/Tween buffer and incubated with HRP-conjugated secondary antibodies (dilution 1:5,000) for 1 h at RT. Following the secondary antibody incubation step, membranes were washed twice with 0.3% PBS/Tween buffer for 10 min and once with 0.05% PBS/Tween buffer. The blots were developed using ECL Plus Western Blotting Substrate (Thermo Fisher Scientific) and visualized on x-ray films (Carestream). For certain blots, membranes were stripped for 30 s at RT and washed three times for 5 min with 0.05% PBST before being blocked and probed with the next antibody. Densitometric analysis was carried out using ImageJ software.

### Co-IP assay

For the co-IP assay, cells were lysed in ice-cold TAP lysis buffer (20 mM Tris-Cl, pH 8.0, 150 mM NaCl, 0.5% NP-40, 1 mM MgCl_2_, 1 mM Na_3_VO_4_, 1 mM NaF, 1 mM PMSF, and 1X protease inhibitor cocktail) on a rotating Hula mixer (Thermo Fisher Scientific) for 30 min at 4°C. Lysates were centrifuged at 16,000 × *g* for 10 min at 4°C, and post-nuclear supernatants (PNS) were collected. The PNS was incubated with the indicated antibody-conjugated resin beads for 3 h at 4°C or overnight in the case of endogenous IP, followed by washes with 0.1% NP-40 TAP lysis buffer. Protein complexes were eluted by boiling the beads in 1X SDS sample loading buffer at 100°C for 10 min. Samples were analyzed by SDS-PAGE followed by immunoblotting as described previously.

### Recombinant protein purification

Bacterial expression vectors encoding GST- or His-tagged proteins were transformed into the *Escherichia coli* Rosetta (DE3) strain. For setting up the primary culture, a single colony was inoculated in Luria–Bertani broth (Difco) containing antibiotics and incubated at 37°C for 12 h. 1% of the primary culture was used to set up the secondary culture and incubated at 37°C until the O.D. at 600 nm reached 0.4–0.6. Protein expression was induced by adding 0.3 mM IPTG (Sigma-Aldrich), followed by incubation for 16–18 h at 18°C. Cells were harvested by centrifugation at 3,542 × *g* for 10 min and washed with 1X PBS or sonication buffer (50 mM Tris and 150 mM NaCl, pH 8.0) in the case of the GAP assay. The pellet was resuspended again in sonication buffer supplemented with 1 mM PMSF (Sigma-Aldrich) and a protease inhibitor tablet (Roche). Bacterial lysis was done by sonication, and the lysates were centrifuged at 15,557 × *g* for 45 min. The resulting supernatants were mixed with glutathione-conjugated agarose resin for purifying GST-tagged proteins (G-Biosciences) or Ni-NTA agarose (Takara) for purifying His-tagged proteins for 2–3 h at 4°C on a Hula mixer. The beads were washed five times with wash buffer: 20 mM Tris and 150 mM NaCl (pH 8.0) (for GST-tagged proteins) or 50 mM Tris, 300 mM NaCl, and 10 mM imidazole (pH 8.0) (for His-tagged proteins) to eliminate non-specific proteins.

GST-tagged proteins were eluted from glutathione-conjugated agarose resin using 50 mM Tris and 10 mM glutathione (pH 8.0), while His-tagged proteins were eluted using a buffer containing 50 mM Tris, 300 mM NaCl, and 250 mM imidazole. The eluted recombinant proteins were concentrated using Amicon Ultra centrifugal filter units (Millipore) and dialyzed twice in a buffer containing 50 mM Tris and 1 mM dithiothreitol for GST-tagged proteins or 50 mM Tris and 300 mM NaCl (pH 8.0) for His-tagged proteins. The concentration of recombinant proteins was measured by the BCA assay.

### GST-pulldown assay

For performing the GST-pulldown experiments, HEK293T cells were lysed in ice-cold TAP lysis buffer containing 20 mM Tris (pH 8.0), 150 mM NaCl, 0.5% NP-40, 1 mM MgCl_2_, 1 mM Na_3_VO_4_, 1 mM NaF, 1 mM PMSF, and protease inhibitor cocktail. Lysates were incubated for 30 min on a Hula mixer and centrifuged at 16,000 × *g* for 10 min. The supernatant was incubated with purified GST-tagged protein (5 µg) immobilized on glutathione-conjugated agarose resin and incubated for 2 h at 4°C. The resin was washed three times with wash buffer containing 20 mM Tris (pH 8.0), 150 mM NaCl, 0.1% NP-40, 1 mM MgCl_2_, 1 mM Na_3_VO_4_, and 1 mM NaF to remove non-specifically bound proteins. Protein complexes were eluted by boiling the resin in 1X SDS sample loading buffer at 100°C for 10 min and analyzed by SDS-PAGE followed by immunoblotting.

For nucleotide-dependent GST-pulldown assays, 5 µg of GST-Arl8b protein immobilized on glutathione-conjugated agarose beads was preloaded with 20 mM GTP (Jena Biosciences) or GDP (Sigma-Aldrich) in nucleotide-loading buffer (20 mM Tris, 2 mM EDTA, 10 mM MgCl_2_, and 25 mM EDTA) at 37°C for 30 min. After adding the lysates to nucleotide-loaded beads, 5 mM GTP or GDP along with 5 mM MgCl_2_ was added and incubated for 30 min at 4°C for 2 h on a Hula mixer. Following incubation, beads were washed three times with wash buffer containing 20 mM Tris (pH 8.0), 150 mM NaCl, 0.1% NP-40, 1 mM MgCl_2_, 1 mM Na_3_VO_4_, and 1 mM NaF. The bound proteins were analyzed by SDS-PAGE and immunoblotting.

### Yeast two-hybrid assay

The yeast two-hybrid assay was performed using Matchmaker Gold Yeast Two-Hybrid System (Clontech) according to the protocol described previously (Sharma et al., 2021). Briefly, the bait gene was cloned into the GAL4 DNA-binding domain vector (pGBKT7), and the prey gene was cloned into the GAL4 activation domain vector (pGADT7). The plasmids were co-transformed into *Saccharomyces cerevisiae* Y2H Gold Strain (Takara Bio Inc.). Transformants were plated on double-dropout (-Leu/-Trp) medium plates and incubated at 30°C for 3 days. Colonies were then spotted on double-dropout (-Leu/-Trp) and triple-dropout (Leu/-Trp/-His) medium plates to assess viability and protein–protein interactions, respectively.

### Immunopurification of lysosomes

To immunopurify lysosomes, the Lyso-IP method with modification was carried out as described previously (Abu-Remaileh et al., 2017; Walia et al., 2024). Briefly, HEK293T cells that were stably expressing TMEM192-2x-FLAG were treated with the desired siRNAs for 60–72 h. After knockdown, cells were harvested, washed with 1X PBS, and homogenized in ice-cold KPBS buffer (136 mM KCl, 10 mM KH_2_PO_4_ with pH adjusted to 7.25 using KOH) using a Dounce homogenizer (30 strokes). The homogenate was transferred to a microcentrifuge tube and centrifuged at 1,000 × *g* for 2 min to remove nuclei and intact cells. The resulting supernatant was incubated with 15 μl slurry of anti-FLAG antibody-conjugated agarose beads for 15 min at 4°C using a Hula mixer to allow lysosome capture. The beads were washed gently six times with KPBS buffer, and bound lysosomes were eluted in 1X Laemmli buffer by boiling prior to SDS-PAGE and immunoblotting.

### Immunopurification of REs

The immunopurification of REs was performed as described previously (Hundley et al., 2024) with some modifications. Briefly, HeLa cells that were stably expressing 3x-FLAG-TFR1 were treated with the desired siRNAs and incubated for 60–72 h. After knockdown, cells were harvested, washed with 1X PBS, and homogenized in ice-cold KPBS buffer (25 mM KCl, 100 mM KH_2_PO_4_ with pH adjusted to 7.2 using KOH) using a Dounce homogenizer (50 strokes). The homogenate was transferred to a microcentrifuge tube and centrifuged at 1,000 × *g* for 5 min at 4°C. The resulting supernatant was incubated with 15 μl slurry of anti-FLAG antibody-conjugated agarose beads for 30 min at 4°C using a Hula mixer to allow binding to endosomes. The beads were washed gently five times with KPBS buffer, and bound endosomes were eluted in 1X Laemmli buffer by boiling prior to SDS-PAGE and immunoblotting.

### Immunogold EM

To perform immunogold EM, HeLa cells treated with control or TBC1D9B siRNA for 72 h were gently detached using 5 mM EDTA. Approximately 1 ml of cell suspension was layered over 200 μl of 4% PFA (Electron Microscopy Sciences) prepared in 0.1 M sodium phosphate buffer (pH 7.4) in a 1.5-ml microcentrifuge tube and centrifuged at 750 × *g* for 3 min. The supernatant was gently removed, and fresh fixative was layered over the cell pellet without resuspending and kept at RT for 3 h in the dark. The fixative was further replaced by 0.25% formaldehyde (Sigma-Aldrich) prepared in 1X PBS for storage. Prior to freezing in liquid nitrogen, cell pellets were infiltrated with 2.3 M sucrose in PBS containing 0.2 M glycine for 15 min to quench free aldehyde groups. Frozen samples were sectioned at −120°C using a cryo-ultramicrotome, and ultrathin sections were collected on formvar/carbon-coated copper grids. Grids were placed on 2% gelatin and stored at 4°C. Grids were thawed and blocked in 1% BSA for 10 min, followed by 30-min incubation with primary mouse anti-LAMP1 antibody diluted (1:50) in 1% BSA in PBS, followed by rabbit anti-mouse secondary antibody (dilution 1:50) for 30 min. The grids were washed in four large drops of PBS for a total of 15 min, then incubated with Protein A–15-nm gold conjugate particles (diluted in 1% BSA) for 20 min. Afterward, grids were washed in four drops of PBS for 15 min and six drops of distilled water. The grids were contrasted and embedded on ice in 0.3% uranyl acetate in 2% methyl cellulose for 10 min and examined using an electron microscope (1200EX; JEOL), and images were recorded with an AMT 2k CCD camera at the Harvard Medical School Electron Microscopy Core Facility (Boston, USA). To measure the diameter of LAMP1-positive endosomes from immuno-EM micrographs, the “Line” tool of Fiji software was used to draw a straight line across the widest part of each vesicle or compartment, and the corresponding diameter was recorded.

### GAP assay

GTPase activity was measured by quantifying the amount of free inorganic phosphate (P_i_) released upon GTP hydrolysis using the malachite green assay following a modified protocol described previously (Wang et al., 2018). Briefly, reactions were assembled in a 96-well plate, with a total volume of 30 μl per well. Each reaction contained 5 µM substrate protein (GST-Rab5a, Rab11a, and Rab14), 1 µM enzyme (His-TBC1D9B), 5 mM MgCl_2_, and 1 mM GTP (Thermo Fisher Scientific) or GDP (Sigma-Aldrich), all prepared in reaction buffer consisting of 20 mM Tris-HCl (pH 8.0) and 150 mM NaCl. Following the addition of GTP or GDP, 200 μl of malachite green reagent (freshly prepared by dissolving 17 mg of malachite green, 525 mg of sodium molybdate, 4.132 ml of HCl, and 0.1% Triton X-100 in 50 ml of deionized water and filtered through a 0.45-µm syringe filter) was added to each well at desired time points to stop the reaction. After 2 min, the colorimetric reaction was quenched with 25 μl of 34% sodium citrate (prepared by dissolving 3.4 g sodium citrate in 10 ml deionized water). The plate was incubated at RT for 25 min, protected from light, and absorbance was recorded at 650 nm using a plate reader.

### EGFR degradation assay

To visualize EGFR turnover, HeLa cells seeded on glass coverslips were treated with the indicated siRNAs for 50 h and then transfected with the desired plasmids. 20 h after transfection, the media of the cells were changed with fresh DMEM containing 10% FBS. The unlabeled EGF (Invitrogen) was added at a final concentration of 100 ng/ml for 2 h at 37°C. Following this, the cells were fixed with 4% PFA prepared in PHEM buffer and immunostained with primary mouse anti-EGFR antibody (1:500), followed by Alexa Fluor–conjugated secondary antibody (dilution 1:500) staining as described previously. Single-plane confocal images were captured using an LSM-710 confocal microscope (Carl Zeiss). EGFR fluorescence intensity was quantified using ImageJ software. The CTCF values were calculated using the formula: CTCF = Integrated density - (area of selected cell × mean fluorescence of background).

### BODIPY FL-BSA trafficking assay

HeLa cells transfected with desired siRNAs for 72 h were incubated with BODIPY FL-conjugated BSA (20 µg/ml; BioVision) prepared in phenol red–free complete DMEM (Gibco) for 6 h at 37°C. Following incubation, the media were removed, and cells were harvested by trypsinization, washed, and resuspended in ice-cold 1X PBS. Samples were analyzed by flow cytometry using a BD FACSAria Fusion cytometer, and data acquisition was performed with BD FACSDiva software (version 8.0.1; BD Biosciences). The data analysis was done using BD FlowJo version 10.0.1.

### Puromycin-induced aggregate formation and clearance

To assess aggregation formation, control or TBC1D9B siRNA-depleted HeLa cells were treated with puromycin (3 µg/ml) for 2 h at 37°C. Following treatment, cells were fixed and subjected to immunostaining using a rabbit anti-p62 antibody (dilution 1:1,000). For the aggregation clearance, puromycin-treated cells were rinsed twice with 1X PBS and then incubated in fresh complete growth media for 3 h. After the clearance period, cells were fixed and immunostained for confocal imaging as described above.

### Statistical analysis

Quantified data were analyzed using GraphPad Prism 8.0. All representative graphs display data as the mean ± standard error of the mean (SEM), unless stated otherwise. All datasets were derived from three independent biological replicates, unless stated otherwise. The graphs are plotted as SuperPlots, in which individual data points from each biological replicate are shown as distinct colored dots, and larger dots represent the mean value of each replicate. Data distribution was assumed to be normal, although this was not formally tested. Statistical analyses were performed on the means of three independent experiments. Unpaired Student’s *t* test was used for comparisons between two groups (Fig. 1 G; Fig. 2, D and E; Fig. 3 B; Fig. 4, D and F; Fig. 7, B and D; Fig. 8, F and I; Fig. 9 E; Fig. S1, J, L, and N; Fig. S2 C; and Fig. S3, C and F). For comparisons among more than two groups, one-way ANOVA was used, followed by Dunnett’s post hoc test for comparisons with the control group (Fig. 1 J; Fig. 2 B; Fig. 4 B; Fig. 8 B; Fig. S1 P; and Fig. S2, E and G) and Tukey’s post hoc test for pairwise comparisons between groups (Fig. 6, B and C). For datasets where one group was normalized, one-sample *t* test was used (Fig. 1, B and D; Fig. 3 E; Fig. 4, H and I; Fig. 6 G; Fig. 7, F and G; Fig. 8 D; Fig. 9, A and C; Fig. S1, A, C, E–G, and J; Fig. S3 D; and Fig. S5, B–D). The P-values and the number of experiments (*n*) are indicated in figure legends.

### Online supplemental material

Fig. S1 shows increased surface LAMP1 and surface LAMP2 levels upon Arl8b depletion across different cell lines, and increased surface LAMP1 levels upon Arl8b depletion are independent of mature lysosomal exocytosis. Fig. S2 shows that Arl8b-Halo localizes to newly synthesized post-endocytic LAMP1 vesicles after 75–90 min of biotin addition. Fig. S3 shows that Arl8b colocalizes and transiently interacts with a subset of AP-3–positive endosomes, which represent the sorting station for the newly synthesized LAMP1 to active lysosomes. Fig. S4 shows that TBC1D9B is a Rab11a GAP and interacts with Arl8b via its N-terminal GRAM1 domain, and Arl8b also interacts with TBC1D9A. Fig. S5 shows that TBC1D9B depletion leads to increased surface LAMP1 levels across different cell lines and leads to altered lysosomal characteristics. Video 1 shows the time lapse of newly synthesized LAMP1 delivery to active lysosomes. Video 2 shows the dynamic and transient kiss-and-run events of newly synthesized LAMP1 with active lysosomes. Video 3 shows Arl8b-Halo localization on newly synthesized LAMP1 vesicles over time. Video 4 shows delivery of newly synthesized LAMP1 to active lysosomes in control and Arl8b-depleted cells. Video 5 shows the dynamics of Arl8b-positive vesicles interacting with AP-3–positive endosomes. Video 6 shows the colocalization of TBC1D9B with LAMP1-RFP or LTR upon Arl8b overexpression. Video 7 shows the dynamics of Arl8b, TBC1D9B, and RUFY1/Rab14. Video 8 shows the delivery of newly synthesized LAMP1 to active lysosomes in control and TBC1D9B-depleted cells. Video 9 shows the dynamics of dextran-loaded terminal compartments in control and TBC1D9B-depleted cells. Table S1 provides the AlphaMissense score and pathogenicity predicted for TBC1D9A and TBC1D9B mutants. Table S2 is the protein sequence alignment of human TBC1D9A and TBC1D9B. Table S3 is a list of DNA constructs used in this study. Table S4 is a list of antibodies used in this study. Data S1 shows source data for graphs from Fig. 1 as an Excel file. Data S2 shows source data for graphs from Fig. 2 as an Excel file. Data S3 shows source data for graphs from Fig. 3 as an Excel file. Data S4 shows source data for graphs from Fig. 4 as an Excel file. Data S5 shows source data for densitometric calculation for blot in Fig. 5 as an Excel file. Data S6 shows source data for graphs from Fig. 6 as an Excel file. Data S7 shows source data for graphs from Fig. 7 as an Excel file. Data S8 shows source data for graphs and densitometric calculation for blot from Fig. 8 as an Excel file. Data S9 shows source data for graphs from Fig. 9 as an Excel file. Data S10 shows source data for graphs and densitometric calculation for blots from Fig. S1 as an Excel file. Data S11 shows source data for graphs and densitometric calculation for blot from Fig. S2 as an Excel file. Data S12 shows source data for graphs and densitometric calculation for blots from Fig. S3 as an Excel file. Data S13 shows source data for graphs from Fig. S4 as an Excel file. Data S14 shows source data for graphs and densitometric calculation for blot from Fig. S5 as an Excel file.

## Declaration for use of generative AI-assisted technologies in manuscript preparation

During the preparation of this work, the authors sparingly used QuillBot and Nature Research Assistant to refine sentence structure and ensure grammatical accuracy.

## Supplementary Material

Table S1 shows AlphaMissense score and pathogenicity predicted for TBC1D9A and TBC1D9B mutants.

Table S2 shows protein sequence alignment of human TBC1D9A and TBC1D9B.

Table S3 shows list of DNA constructs used in this study.

Table S4 shows list of antibodies used in this study.

Data S1 shows source data for graphs from Fig. 1 as an Excel file.

Data S2 shows source data for graphs from Fig. 2 as an Excel file.

Data S3 shows source data for graphs from Fig. 3 as an Excel file.

Data S4 shows source data for graphs from Fig. 4 as an Excel file.

Data S5 shows source data for densitometric calculation for blot in Fig. 5 as an Excel file.

Data S6 shows source data for graphs from Fig. 6 as an Excel file.

Data S7 shows source data for graphs from Fig. 7 as an Excel file.

Data S8 shows source data for graphs and densitometric calculation for blot from Fig. 8 as an Excel file.

Data S9 shows source data for graphs from Fig. 9 as an Excel file.

Data S10 shows source data for graphs and densitometric calculation for blots from Fig. S1 as an Excel file.

Data S11 shows source data for graphs and densitometric calculation for blot from Fig. S2 as an Excel file.

Data S12 shows source data for graphs and densitometric calculation for blots from Fig. S3 as an Excel file.

Data S13 shows source data for graphs from Fig. S4 as an Excel file.

Data S14 shows source data for graphs and densitometric calculation for blot from Fig. S5 as an Excel file.

SourceData F4 is the source file for Fig. 4.

SourceData F5 is the source file for Fig. 5.

SourceData F6 is the source file for Fig. 6.

SourceData F7 is the source file for Fig. 7.

SourceData F8 is the source file for Fig. 8.

SourceData FS1 is the source file for Fig. S1.

SourceData FS2 is the source file for Fig. S2.

SourceData FS3 is the source file for Fig. S3.

SourceData FS4 is the source file for Fig. S4.

SourceData FS5 is the source file for Fig. S5.

## Data Availability

All relevant data supporting the findings of the study are included in the manuscript and supplementary material.
