# Supplementary material for: Arl8b inactivates the Rab11a recycling pathway to promote LAMP1 sorting and lysosome biogenesis
Source: J Cell Biol. 2026 May 21;225(7):e202509040. doi: 10.1083/jcb.202509040 (PMC13193097; doi:10.1083/jcb.202509040)
Supplement: Table S1 — shows AlphaMissense score and pathogenicity predicted for TBC1D9A and TBC1D9B mutants. [file jcb_202509040_tables1.docx]

**Supplementary Table I:** AlphaMissense score and pathogenicity predicted for TBC1D9A and TBC1D9B mutants.

| **Mutants** | **AlphaMissense Score** | **Pathogenicity** |
| --- | --- | --- |
| TBC1D9A p. Glu95Ala | 0.886 | Likely pathogenic |
| TBC1D9B p. Glu91Ala | 0.841 | Likely pathogenic |
| TBC1D9B p. Leu95Ala | 0.9 | Likely pathogenic |
