# Supplementary material for: Arl8b inactivates the Rab11a recycling pathway to promote LAMP1 sorting and lysosome biogenesis
Source: J Cell Biol. 2026 May 21;225(7):e202509040. doi: 10.1083/jcb.202509040 (PMC13193097; doi:10.1083/jcb.202509040)
Supplement: Table S2 — shows protein sequence alignment of human TBC1D9A and TBC1D9B. [file jcb_202509040_tables2.docx]

**Supplementary Table II:** Protein sequence alignment of human TBC1D9A and TBC1D9B

*Note*:

Arl8b binding residues are shown in **green** color

Arginine (R) finger within in IxxDxxR motif and glutamine finger within YxQ motif residues are shown in **red** color

IxxDxxR and YxQ motifs are highlighted in **yellow** color

# Identity: 811/1302 (62.3%)

# Similarity: 971/1302 (74.6%)

TBC1D9A 1 MWVNPEEVLLANALWITERANPYFILQRRKGHAGDGGGGGGLAGLLVGTL 50

||::|||||:|||||:||||||:|:||||:|| |.||||.|||||||

TBC1D9B 1 MWLSPEEVLVANALWVTERANPFFVLQRRRGH----GRGGGLTGLLVGTL 46

Arl8b-binding site

TBC1D9A 51 DVVLDSSARVAPYRILYQTPDSLVYWTIACGGSRKEITEHWEWL**E**QNL**L**Q 100

||||||||||||||||:||.||.||||:|||.||||||:||||||.||||

TBC1D9B 47 DVVLDSSARVAPYRILHQTQDSQVYWTVACGSSRKEITKHWEWL**E**NNL**L**Q 96

TBC1D9A 101 TLSIFENENDITTFVRGKIQGIIAEYNKINDVKEDDDTEKFKEAIVKFHR 150

|||||::|.||||||:|||.|||||.||....:.|:|..|||||.:|..:

TBC1D9B 97 TLSIFDSEEDITTFVKGKIHGIIAEENKNLQPQGDEDPGKFKEAELKMRK 146

TBC1D9A 151 LFGMPEEEKLVNYYSCSYWKGKVPRQGWMYLSINHLCFYSFLMGREAKLV 200

.|||||.||||||||||||||:||||||:||::|||||||||:|:|..||

TBC1D9B 147 QFGMPEGEKLVNYYSCSYWKGRVPRQGWLYLTVNHLCFYSFLLGKEVSLV 196

TBC1D9A 201 IRWVDITQLEKNATLLLPDVIKVSTRSSEHFFSVFLNINETFKLMEQLAN 250

::|||||:||||||||.|:.|:|.||..|.|||:||||.|||||||||||

TBC1D9B 197 VQWVDITRLEKNATLLFPESIRVDTRDQELFFSMFLNIGETFKLMEQLAN 246

TBC1D9A 251 IAMRQLLDNEGFEQDRSLPKLKRKSPKKVSALKRDLDARAKSERYRALFR 300

:|||||||:|||.:|::||:..|.. :.:||||||||||||:|.|||.||

TBC1D9B 247 LAMRQLLDSEGFLEDKALPRPIRPH-RNISALKRDLDARAKNECYRATFR 295

TBC1D9A 301 LPKDEKLDGHTDCTLWTPFNKMHILGQMFVSTNYICFTSKEENLCSLIIP 350

||:||:|||||.|||||||||:||.||||:|.|||||.||||:.|.||||

TBC1D9B 296 LPRDERLDGHTSCTLWTPFNKLHIPGQMFISNNYICFASKEEDACHLIIP 345

TBC1D9A 351 LREVTIVEKADSSSVLPSPLSISTRNRMTFLFANLKDRDFLVQRISDFLQ 400

||||||||||||||||||||||||:::|||||||||||||||||||||||

TBC1D9B 346 LREVTIVEKADSSSVLPSPLSISTKSKMTFLFANLKDRDFLVQRISDFLQ 395

TBC1D9A 401 QTTSKIYSDKEFAGSYNSSDDEVYSRPSSLVSSSPQRSTSSDAD-----G 445

:|.|| ..||..|....|.. ||:..|.:||..:...|. .

TBC1D9B 396 KTPSK------QPGSIGSRKASVVD-PSTESSPAPQEGSEQPASPASPLS 438

TBC1D9A 446 ERQFNLNGNSVPTATQTLMTMYRRRSP-EEFNPKLAKEFLKEQAWKIHFA 494

.|| :......|||:|.|:.::::.|| |:...|.|||.:||::|.|||.

TBC1D9B 439 SRQ-SFCAQEAPTASQGLLKLFQKNSPMEDLGAKGAKEKMKEESWHIHFF 487

TBC1D9A 495 EYGQGICMYRTEKTRELVLKGIPESMRGELWLLLSGAINEKATHPGYYED 544

|||:|:|||||.|||.|||||||||:|||||||.|||.||..||||||.:

TBC1D9B 488 EYGRGVCMYRTAKTRALVLKGIPESLRGELWLLFSGAWNEMVTHPGYYAE 537

IxxDxxR motif

TBC1D9A 545 LVEKSMGKYNLATEEIERDLH**R**SLPEHPAFQNEMGIAALRRVLTAYAFRN 594

|||||.|||:|||||||||||||:|||||||||:||||||||||||||||

TBC1D9B 538 LVEKSTGKYSLATEEIERDLH**R**SMPEHPAFQNELGIAALRRVLTAYAFRN 587

YxQ motif

TBC1D9A 595 PNIG**Y**C**Q**AMNIVTSVLLLYAKEEEAFWLLVALCERMLPDYYNTRVVGALV 644

|.|||||||||||||||||..|||||||||||||||||||||||||||||

TBC1D9B 588 PTIG**Y**C**Q**AMNIVTSVLLLYGSEEEAFWLLVALCERMLPDYYNTRVVGALV 637

TBC1D9A 645 DQGVFEELARDYVPQLYDCMQDLGVISTISLSWFLTLFLSVMPFESAVVV 694

|||:||||.||::|||.:.||||||||:|||||||||||||||||||||:

TBC1D9B 638 DQGIFEELTRDFLPQLSEKMQDLGVISSISLSWFLTLFLSVMPFESAVVI 687

TBC1D9A 695 VDCFFYEGIKVIFQLALAVLDANVDKLLNCKDDGEAMTVLGRYLDSVTNK 744

||||||||||||.|:||||||||:::||.|.|:|||||:||||||:|.||

TBC1D9B 688 VDCFFYEGIKVILQVALAVLDANMEQLLGCSDEGEAMTMLGRYLDNVVNK 737

TBC1D9A 745 DSTLPPIPHLHSLLSDDVEPYPEVDIFRLIRTSYEKFGTIRADLIEQMRF 794

.|..||||||.:|||...:|..|||||.|::.|||||.::||:.||||||

TBC1D9B 738 QSVSPPIPHLRALLSSSDDPPAEVDIFELLKVSYEKFSSLRAEDIEQMRF 787

TBC1D9A 795 KQRLKVIQTLEDTTKRNVVRTIVTETSFTIDELEELYALFKAEHLTSCYW 844

||||||||:||||.||:|||.|..:..|:|:|||:||.:|||:||.|.||

TBC1D9B 788 KQRLKVIQSLEDTAKRSVVRAIPVDIGFSIEELEDLYMVFKAKHLASQYW 837

TBC1D9A 845 GGSSNALDRHDPSLPYLEQYRIDFEQFKGMFALLFPWACGTHSDVLASRL 894

|.|.....|.|||||||||||||..||:.:||.|.|||||:|:.:||.|:

TBC1D9B 838 GCSRTMAGRRDPSLPYLEQYRIDASQFRELFASLTPWACGSHTPLLAGRM 887

TBC1D9A 895 FQLLDENGDSLINFREFVSGLSAACHGDLTEKLKLLYKMHVLPEPSSDQD 944

|:|||||.||||||:|||:|:|...|||||||||:|||:| || |:...:

TBC1D9B 888 FRLLDENKDSLINFKEFVTGMSGMYHGDLTEKLKVLYKLH-LP-PALSPE 935

TBC1D9A 945 EPDSAFEATQYFFEDITPECTHVV-------------GLDSRSKQGADDG 981

|.:||.||..||.||.:.|.:.:. .|....::|:.

TBC1D9B 936 EAESALEAAHYFTEDSSSEASPLASDLDLFLPWEAQEALPQEEQEGSG-- 983

TBC1D9A 982 FVTVSLKPDKGKRANSQENRNYLRLWTPENKSKSKNAKDLPKLNQGQFIE 1031

|.:..:.|..:|.:.|:|||:|..|.:::.:..|||||:||.||||

TBC1D9B 984 ----SEERGEEKGTSSPDYRHYLRMWAKEKEAQKETIKDLPKMNQEQFIE 1029

TBC1D9A 1032 LCKTMYNMFSEDPNEQELYHATAAVTSLLLEIGEVGKLFVAQPAKEGGSG 1081

||||:||||||||.||:||||.|.|.||||.||||||.|.|:..::....

TBC1D9B 1030 LCKTLYNMFSEDPMEQDLYHAIATVASLLLRIGEVGKKFSARTGRKPRDC 1079

TBC1D9A 1082 GS------GPSCHQGIPGVLFPKKGPGQPYVVESVEPLPASLAPDSEEHS 1125

.: .|..||.....|.| ||:..|.::

TBC1D9B 1080 ATEEDEPPAPELHQDAARELQP----------------PAAGDPQAK--- 1110

TBC1D9A 1126 LGG--------QMEDIKLEDSSPRDNGACSSMLISDDDTKDDSSMSSYSV 1167

.|| |...:.:|..|....|: .|.|:|||:||||.|||||||

TBC1D9B 1111 AGGDTHLGKAPQESQVVVEGGSGEGQGS-PSQLLSDDETKDDMSMSSYSV 1159

TBC1D9A 1168 LSAGSHEEDKLHCEDIGEDTVLVRSGQGTAALPRST---SLDRDWAITFE 1214

:|.|| |.|||:.:||||| .|.|..|.:. ::|.||.|:||

TBC1D9B 1160 VSTGS-----LQCEDLADDTVLV---GGEACSPTARIGGTVDTDWCISFE 1201

TBC1D9A 1215 QFLASLLTEPALVKYFDKPVCMMARITSAKNIRMMGKPLTSASDYEISAM 1264

|.|||:|||..||.:|:|.|.:..:|...|.:. :..::|||:|...:

TBC1D9B 1202 QILASILTESVLVNFFEKRVDIGLKIKDQKKVE---RQFSTASDHEQPGV 1248

TBC1D9A 1265 SG 1266

||

TBC1D9B 1249 SG 1250
