# Supplementary material for: Arl8b inactivates the Rab11a recycling pathway to promote LAMP1 sorting and lysosome biogenesis
Source: J Cell Biol. 2026 May 21;225(7):e202509040. doi: 10.1083/jcb.202509040 (PMC13193097; doi:10.1083/jcb.202509040)
Supplement: Table S3 — shows list of DNA constructs used in this study. [file jcb_202509040_tables3.docx]

**Supplementary Table III:** List of DNA constructs used in this study.

| **Plasmid Name** | **Description** | **Source** |
| --- | --- | --- |
| ***Mammalian expression constructs:*** | | |
| SBP-GFP-LAMP1 | RUSH construct expressing the hook protein (Streptavidin-KDEL) and cargo protein (Streptavidin-binding protein-GFP-LAMP1 (wild-type; WT) | Kind gift from Prof. Juan Bonifacino (NIH, USA) |
| SBP-mCherry-LAMP1 | RUSH construct expressing the hook protein (Streptavidin-KDEL) and cargo protein (Streptavidin-binding protein-mCherry-LAMP1 (wild-type; WT) | Kind gift from Prof. Juan Bonifacino (NIH, USA) |
| SBP-GFP-LAMP1 (Y404A) | RUSH construct expressing the hook protein (Streptavidin-KDEL) and cargo protein (Streptavidin-binding protein-GFP-LAMP1 with Y404A mutation | Kind gift from Prof. Juan Bonifacino (NIH, USA) |
| CD63-RFP | CD63 with C-term RFP tag | Kind gift from Prof. Ursula Rescher (University of Münster, Germany) |
| pHTC Halo-vector | Mammalian expression vector with C-term Halo tag | From Promega (G7711) |
| pEGFP-C1 vector | Mammalian expression vector with N-term GFP tag | Kind gift from Prof. Peter Cullen (University of Bristol, UK) |
| Arl8b-Halo | Human Arl8b cloned with C-term Halo tag in pHTC-Halo tag vector | This study |
| Arl8b-Halo (Q75L) | Human Arl8b with Q75L mutation and C-term Halo tag cloned in pHTC-Halo tag vector | This study |
| Arl8b-Halo (T34N) | Human Arl8b with Q75L mutation and C-term Halo tag cloned in pHTC-Halo tag vector | This study |
| pX330-sgRNA-hArl8b | gRNA for targeting human Arl8b cloned in pX330 vector expressing human codon-optimized SpCas9 | This study |
| pTwist-hArl8b-Homology arms-mStayGold-hygromycin | Homology repair donor plasmid for endogenous tagging of Human Arl8b with C-terminal mStayGold tag. | Synthesised from Twist Biosciences |
| Arl8b-GFP | Arl8b with C-term GFP tag | Described previously (Marwaha et al., 2017) |
| AP3 (M1)-mScarlet | AP3 (M1) with C-term mScarlet tag | Kind gift from Prof. Serhiy Pankiv (Institute of Clinical Medicine, Oslo) |
| GFP-TBC1D9B | Human TBC1D9B with N-term GFP tag cloned in pEGFP-C1 vector | This study |
| GFP-TBC1D9B (RYQ →A) | Human TBC1D9B with R559A/Y592A/Q594A mutations and N-term GFP tag cloned in pEGFP-C1 vector | This study |
| GFP-TBC1D9B (E91A) | Human TBC1D9B with E91A mutation and N-term GFP tag cloned in pEGFP-C1 vector | This study |
| GFP-TBC1D9B (1-270 aa) | Human TBC1D9B expressing 1-270 aa and N-term GFP tag cloned in pEGFP-C1 vector | This study |
| GFP-TBC1D9B (1-410 aa) | Human TBC1D9B expressing 1-410 aa and N-term GFP tag cloned in pEGFP-C1 vector | This study |
| GFP-TBC1D9B (401-1250 aa) | Human TBC1D9B expressing 401-1250 aa and N-term GFP tag cloned in pEGFP-C1 vector | This study |
| Mito-Arl8b (Q75L)-HA | Human Arl8b (lacking first 17 aa and Q75L mutation) cloned into the Rab7A (QL)-BirA HA-MAO vector by replacing Rab7 (QL) cassette (Addgene plasmid # 128904) | Described previously (Kumar et al., 2022) |
| Mito-Arl8b (T34N)-HA | Human Arl8b (lacking first 17 aa and T34N mutation) cloned into the Rab7A (QL)-BirA HA-MAO vector by replacing Rab7 (QL) cassette (Addgene plasmid # 128904) | Described previously (Kumar et al., 2022) |
| LAMP1-RFP | LAMP1 with C-terminal RFP tag | From Addgene (#1817) |
| Arl8b-DsRed | Human Arl8b with C-terminal DsRed tag cloned in pdsRedN1 vector | This study |
| RUFY1-Halo | Human RUFY1 with C-term Halo tag cloned in pHTC-Halo tag vector | This study |
| Halo-Rab14 | Human Rab14 with N-term Halo tag cloned in pHTN-Halo tag vector | This study |
| HA-TBC1D9B (WT)  [Rescue construct] | N-terminal HA-tagged human TBC1D9B rescue construct against TBC1D9B siRNA and cloned into the pcDNA3.1(-) vector | This study |
| HA-TBC1D9B (RYQ →A) [Rescue construct] | N-terminal HA-tagged human TBC1D9B (R559A/Y592A/Q594A) rescue construct against TBC1D9B siRNA and cloned into the pcDNA3.1(-) vector | This study |
| HA-TBC1D9B (E91A)  [Rescue construct] | N-terminal HA-tagged human TBC1D9B (E91A) rescue construct against TBC1D9B siRNA and cloned into the pcDNA3.1(-) vector | This study |
| FLAG-TBC1D9B | Human TBC1D9B with N-terminal FLAG tag cloned in pcDNA3.1(-) vector | This study |
| FLAG-TBC1D9B (E91A) | Human TBC1D9B with E91A mutation and N-terminal FLAG tag cloned in pcDNA3.1(-) vector | This study |
| GFP-Rab11a | Rab11a with N-term GFP tag | Kind gift from Prof. Martin Lowe (University of Manchester, UK) |
| Arl8b (WT)-HA | Human Arl8b (WT) with C-terminal HA tag cloned in pcDNA3.1(-) vector | Described previously (Marwaha et al., 2017) |
| Arl8b (Q75L)-HA | Human Arl8b containing Q75L mutation and C-terminal HA tag cloned in pcDNA3.1(-) vector | Described previously (Marwaha et al., 2017) |
| RUFY1-FLAG | Human RUFY1 with C-terminal FLAG tag cloned in pcDNA3.1(+) vector | Described previously (Rawat et al., 2023) |
| pHAGE-3xFLAG-TFR1 | N-terminal 3xFLAG-tagged Transferrin receptor cloned into pHAGE vector | From Addgene (#176490) |
| pLJC5-TMEM192-2xFLAG | C-terminal 2xFLAG-tagged TMEM192 cloned into pLJC5 vector | From Addgene (#102929) |
| ***Bacterial expression constructs:*** | | |
| pDX130-His-mCherry-GFP nanobody | His-mCherry tagged nanobody against GFP | Kind gift from Prof. David C. Gershlick (Cambridge Institute of Medical Research, UK) |
| pGEX-4T3 vector | Bacterial protein expression vector for making N-terminal GST   proteins | Available in the lab |
| GST-Arl8b | Human Arl8b with N-terminal GST tag cloned in pGEX-4T3 vector | Described previously (Marwaha et al., 2017) |
| GST-Rab5a | Human Rab5a with N-terminal GST tag cloned in pGEX6P1 vector | Kind gift from Prof. Sunnando Dutta (IISER Bhopal, India) |
| GST-Rab11a | Human Rab11a with N-terminal GST tag cloned in pGEX-5x vector | Kind gift from Prof. Sunnando Dutta (IISER Bhopal, India) |
| GST-Rab14 | Human Rab14 with N-terminal GST tag cloned in pGEX4T3 vector | This study |
| His-TBC1D9A | Human TBC1D9A cloned in pcold-II vector | This study |
| His-TBC1D9A (RYQ→A) | Human TBC1D9A with R559A/Y592A/Q594A mutations and cloned in pcold-II vector | This study |
| His-TBC1D9B | Human TBC1D9B cloned in pcold-II vector | This study |
| His-TBC1D9B (RYQ→A) | Human TBC1D9B with R559A/Y592A/Q594A mutations and cloned in pcold-II vector | This study |
| ***Yeast two-hybrid constructs:*** | | |
| Gal4AD | GAL4-activation domain expressing yeast two-hybrid vector | Clontech |
| Gal4AD-Arl8b | Human Arl8b cloned in pGADT7 vector | This study |
| Gal4AD-Arl8b (Q75L) | Human Arl8b (Q75L) cloned in pGADT7 vector | This study |
| Gal4AD-Arl8b (T34N) | Human Arl8b (T34N) cloned in pGADT7 vector | This study |
| Gal4AD-TBC1D9A | Human TBC1D9A cloned in pGADT7 vector | This study |
| Gal4AD-TBC1D9A (E95A) | Human TBC1D9A (E95A) cloned in pGADT7 vector | This study |
| Gal4AD-LC3B | Human LC3B cloned in pGADT7 | This study |
| Gal4BD | GAL4-DNA binding domain expressing yeast two-hybrid vector | Clontech |
| Gal4BD-Arl8b (Q75L) | Human Arl8b (Q75L) cloned in pGBKT7 vector | This study |
| Gal4BD-TBC1D9B | Human TBC1D9B cloned in pGBKT7 vector | This study |
| Gal4BD-TBC1D9B (RYQ→A) | Human TBC1D9B (RYQ→A) cloned in pGBKT7 vector | This study |
| Gal4BD-TBC1D9B (E91A) | Human TBC1D9B (E91A) cloned in pGBKT7 vector | This study |
| Gal4BD-TBC1D9B (L95A) | Human TBC1D9B (L95A) cloned in pGBKT7 vector | This study |
| Gal4BD-TBC1D9B (E91A/L95A) | Human TBC1D9B (E91A/L95A) cloned in pGBKT7 vector | This study |
| Gal4BD-LC3B | Human LC3B cloned in pGBKT7 vector | Described previously (Marwaha et al., 2017) |

**References:**

Kumar, G., P. Chawla, N. Dhiman, S. Chadha, S. Sharma, K. Sethi, M. Sharma, and A. Tuli. 2022. RUFY3 links Arl8b and JIP4-Dynein complex to regulate lysosome size and positioning. Nat. Commun. 13, 1540.

Marwaha, R., S.B. Arya, D. Jagga, H. Kaur, A. Tuli, and M. Sharma. 2017. The Rab7 effector PLEKHM1 binds Arl8b to promote cargo traffic to lysosomes. *Journal of Cell Biology*. 216:1051-1070.

Rawat, S., D. Chatterjee, R. Marwaha, G. Charak, G. Kumar, S. Shaw, D. Khatter, S. Sharma, C. de Heus, N. Liv, J. Klumperman, A. Tuli, and M. Sharma. 2023. RUFY1 binds Arl8b and mediates endosome-to-TGN CI-M6PR retrieval for cargo sorting to lysosomes. *J Cell Biol*. 222.
