# Supplementary material for: Arl8b inactivates the Rab11a recycling pathway to promote LAMP1 sorting and lysosome biogenesis
Source: J Cell Biol. 2026 May 21;225(7):e202509040. doi: 10.1083/jcb.202509040 (PMC13193097; doi:10.1083/jcb.202509040)
Supplement: Table S4 — shows list of antibodies used in this study. [file jcb_202509040_tables4.docx]

**Supplementary Table IV**: List of antibodies used in this study.

(IF: Immunofluorescence; WB: Western Blotting; IP: Immunoprecipitation; FCM: Flow Cytometry)

| Antibody | Source | Catalog # |
| --- | --- | --- |
| Alexa Fluor 647-conjugated mouse anti-human CD107A (LAMP1)  (FCM-1:20) | BD Biosciences | 562622 |
| Alexa Fluor 647-conjugated mouse anti-human CD107B (LAMP2)  (FCM-1:20) | Biolegend | 354312 |
| Mouse anti-EGFR  (FCM-1:100 and IF-1:500) | Invitrogen | MA5-13269 |
| Mouse anti-α-tubulin  (WB-1:5000) | Sigma-Aldrich | T9026 |
| Mouse anti-LAMP2  (IF-1:1000 and WB-1:5000) | BD Biosciences | 555803 |
| Rabbit anti-Cathepsin D  (IF-1:500 and WB-1:2000) | Abcam | ab75852 |
| Rabbit anti-Arl8b  (WB-1:1000) | CST | 56085 |
| Mouse anti-Arl8b  (WB-1:5000) | Protein Tech | 67891-1 |
| Rabbit Alpaca anti-mStayGold (WB- 1:1000) | HUABIO | HA710349 |
| Rabbit anti-Vps33a  (WB- 1:2000) | Protein Tech | 16896-1-AP |
| Mouse anti-GFP  (WB-1:3000) | Santa Cruz Biotechnology | sc-9996 |
| Rabbit anti-GFP  (WB-1:2000 and IF- 1:1000) | In-house | -- |
| Rabbit anti-TBC1D9A  (WB-1:2000) | Abcam | ab72236 |
| Mouse anti-AP3  (IF-1:50 and WB- 1: 1000) | Santa Cruz Biotechnology | sc-136277 |
| Rabbit anti-Rab11a  (IF-1:250 and WB- 1:2000) | Abcam | 128913 |
| Rabbit anti-EEA1  (WB- 1:2500) | Abcam | ab2900 |
| Rabbit anti-Rab5  (WB- 1:2000) | CST | 3547 |
| Rabbit anti-Calreticulin  (WB-1:2000) | Abcam | ab92516 |
| Mouse anti-GAPDH  (WB- 1:3000) | Santa Cruz Biotechnology | sc-166374 |
| Mouse anti-Rab7  (WB- 1:1000) | Santa Cruz Biotechnology | sc-376362 |
| Rabbit anti-TBC1D9B  (WB-1:2000) | Abcam | 187168 |
| Rabbit anti-p62  (IF-1:1000) | MBL | PM074 |
| Mouse anti-CI-M6PR  (IF-1:500) | Abcam | ab2733 |
| Rabbit anti-CI-M6PR  (IF-1:500) | Abcam | ab124767 |
| Mouse anti-LAMP1  (IF-1:1000 and WB-1:1000 and Immuno-EM-1:50) | BD Biosciences | 555798 |
| Rabbit anti-LAMP1  (IF-1:2500) | Abcam | ab24170 |
| Rabbit anti-Rab14  (IF-1:500) | Sigma-Aldrich | R0656 |
| Mouse anti-HA  (IF-1:500 and WB-1:3000) | BioLegend | MMS-101P |
| Rabbit anti-HA  (IF-1:250 and WB-1:3000) | Sigma-Aldrich | H6908 |
| Mouse anti-FLAG  (IF-1:500 and WB-1:3000) | Sigma-Aldrich | F1804 |
| Rabbit anti-FLAG  (IF-1:500) | Sigma-Aldrich | F7425 |
| Rabbit anti-FLAG  (WB-1:3000) | Invitrogen | PA1-984B |
| Rabbit anti-Giantin  (IF-1:4000) | Abcam | ab24586 |
| Alexa Fluor 488-conjugated  goat anti-rabbit IgG  (IF-1:500) | Thermo Fisher Scientific | A11034 |
| Alexa Fluor 568-conjugated  goat anti-rabbit IgG  (IF-1:500) | Thermo Fisher Scientific | A11036 |
| Alexa Fluor 647-conjugated  goat anti-rabbit IgG  (IF-1:500) | Thermo Fisher Scientific | A21245 |
| Alexa Fluor 488-conjugated  goat anti-mouse IgG  (IF-1:500 and FCM: 1:2000) | Thermo Fisher Scientific | A11029 |
| Alexa Fluor 568-conjugated  goat anti-mouse IgG  (IF-1:500) | Thermo Fisher Scientific | A11031 |
| Alexa Fluor 647-conjugated  goat anti-mouse IgG   (IF-1:500) | Thermo Fisher Scientific | A21235 |
| HRP-conjugated goat anti-rabbit IgG  (WB-1:5000) | Jackson ImmunoResearch | 111-035-144 |
| HRP-conjugated goat anti-mouse IgG  (WB-1:5000) | Jackson ImmunoResearch | 115-035-166 |
| Mouse IgG-conjugated agarose beads  (IP: 3 μL slurry) | Sigma-Aldrich | A0919 |
| Mouse anti-Arl8-conjugated agarose beads  (IP: 30 μL slurry) | Santa Cruz Biotechnology | sc-398635 AC |
| Anti-FLAG affinity gel  (IP: 15 μL slurry) | BioLegend | 651503 |
| Anti-HA affinity gel  (IP: 13 μL slurry) | Sigma-Aldrich | A2095 |
