# Supplementary figures and images for: Arl8b inactivates the Rab11a recycling pathway to promote LAMP1 sorting and lysosome biogenesis
Source: J Cell Biol. 2026 May 21;225(7):e202509040. doi: 10.1083/jcb.202509040 (PMC13193097; doi:10.1083/jcb.202509040)

Source Data Figure 4

Figure 4G

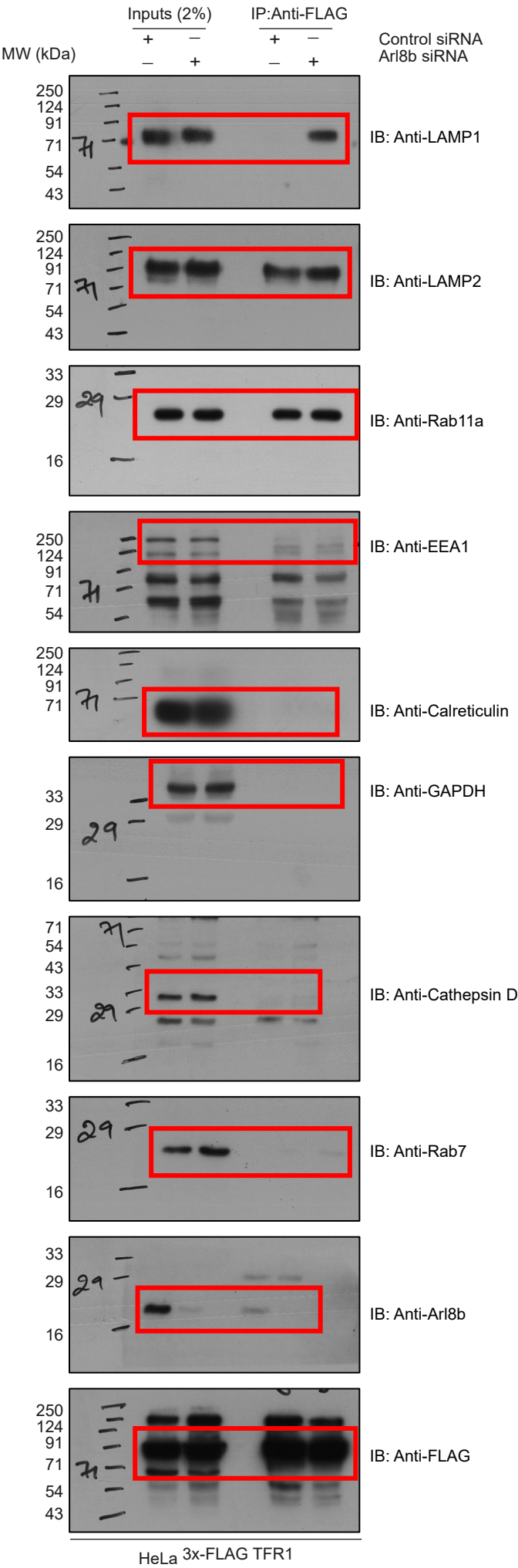

Supplement: SourceData F4 — is the source file for Fig. 4. [file jcb_202509040_sourcedataf4.pdf]

Source Data Figure 5

Figure 5A

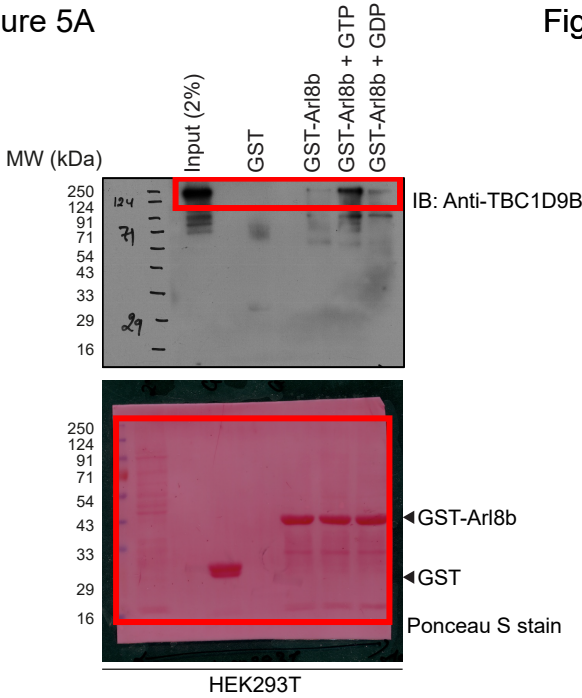

Figure 5B

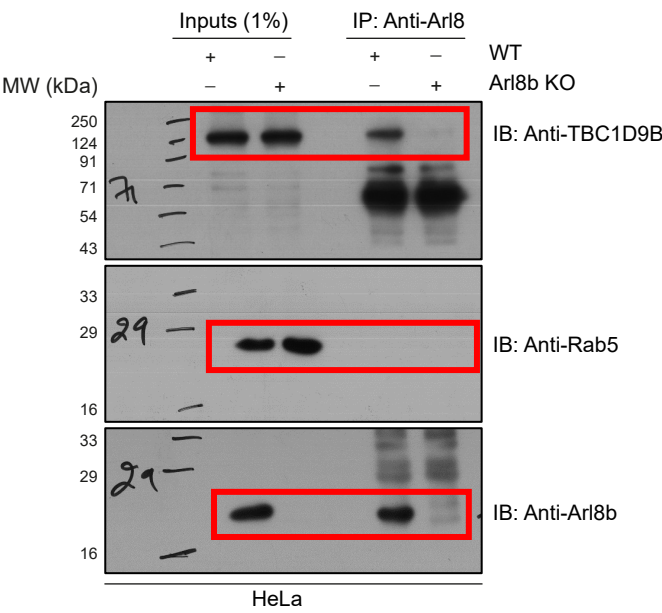

Figure 5D

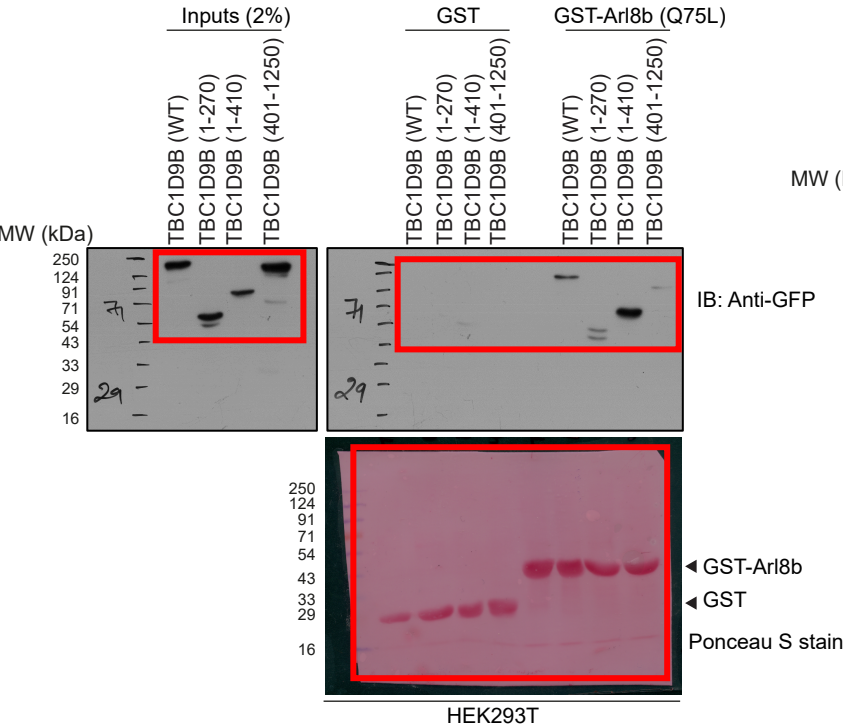

Figure 5E

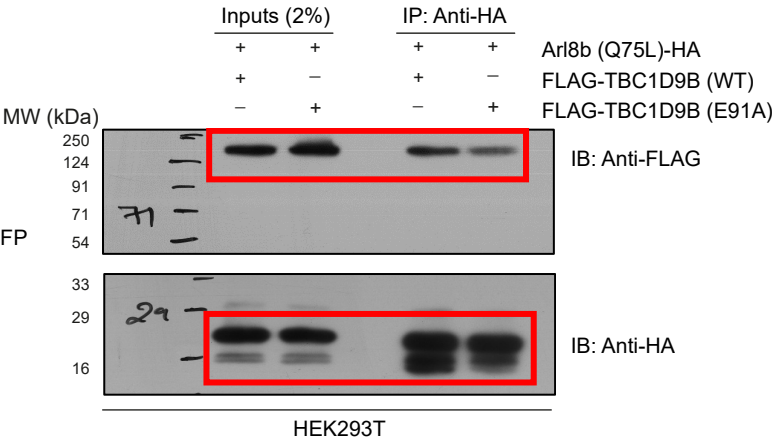

Supplement: SourceData F5 — is the source file for Fig. 5. [file jcb_202509040_sourcedataf5.pdf]

Source Data Figure 6

Figure 6F

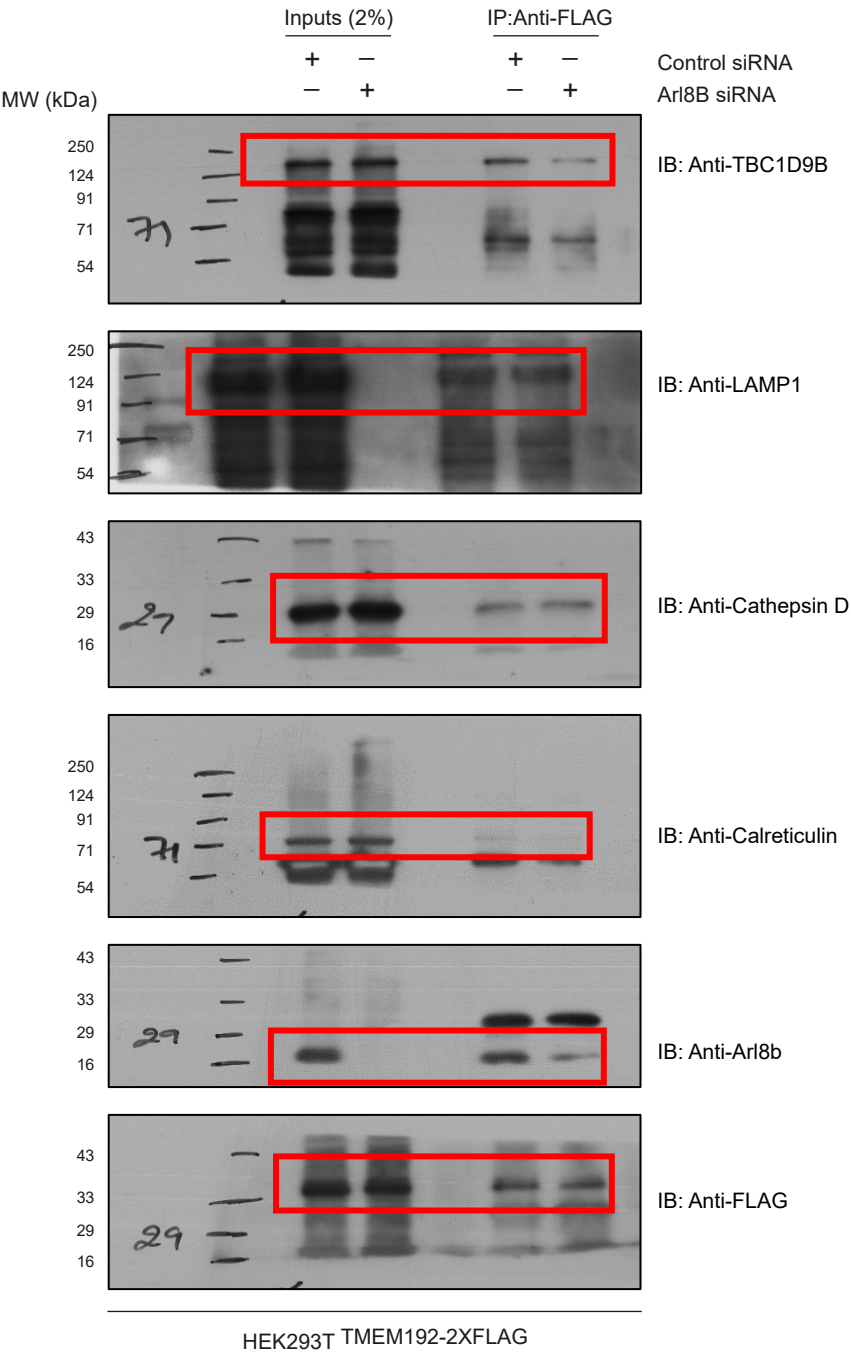

Supplement: SourceData F6 — is the source file for Fig. 6. [file jcb_202509040_sourcedataf6.pdf]

Source Data Figure 7

Figure 7E

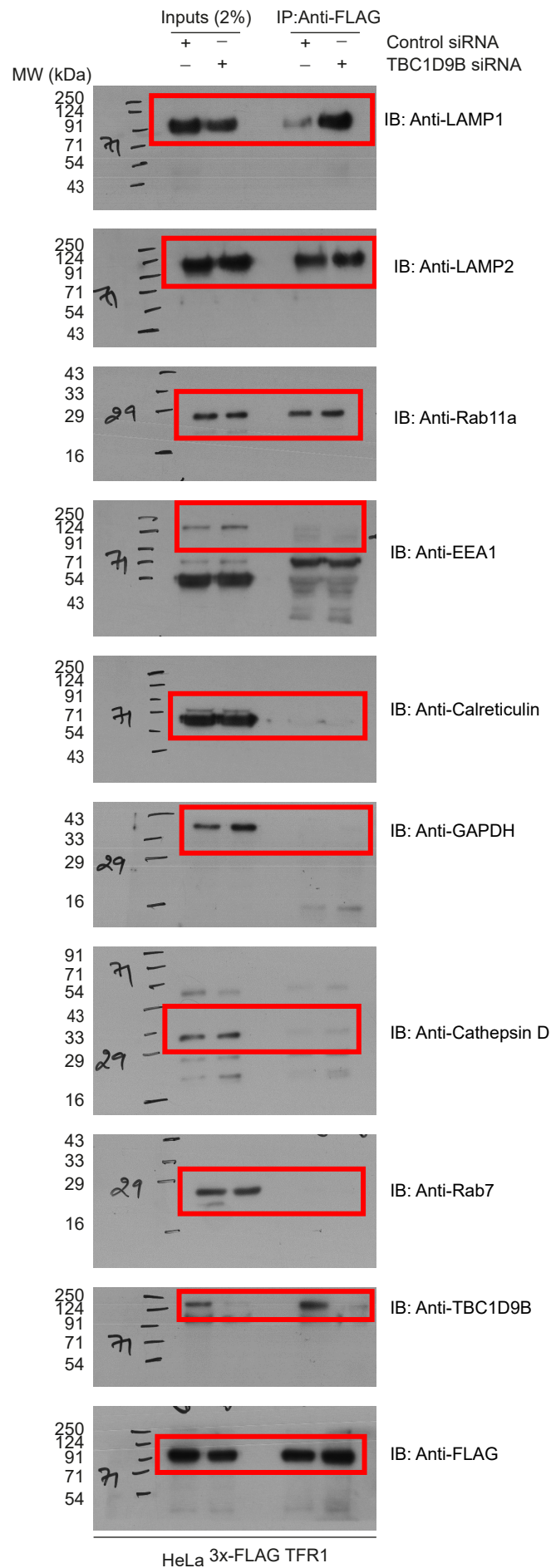

Supplement: SourceData F7 — is the source file for Fig. 7. [file jcb_202509040_sourcedataf7.pdf]

Source Data Figure 8

Figure 8G

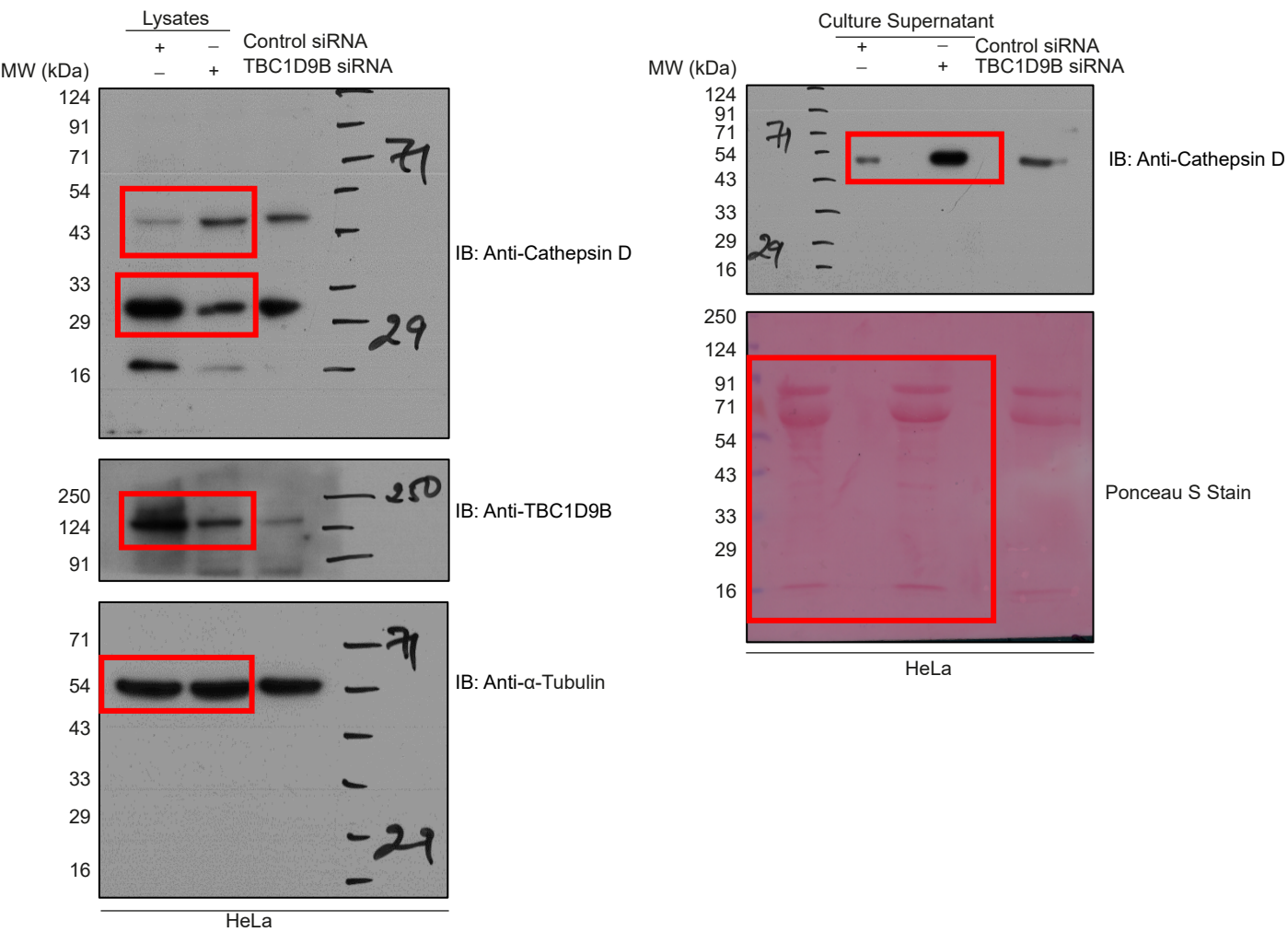

Supplement: SourceData F8 — is the source file for Fig. 8. [file jcb_202509040_sourcedataf8.pdf]

Source Data Supplementary Figure 1

Figure S1A

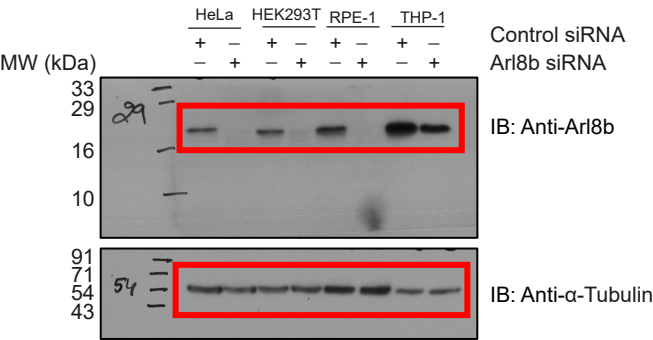

Figure S1B

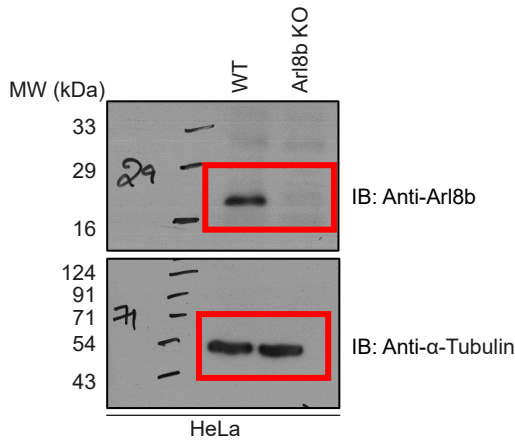

Figure S1H

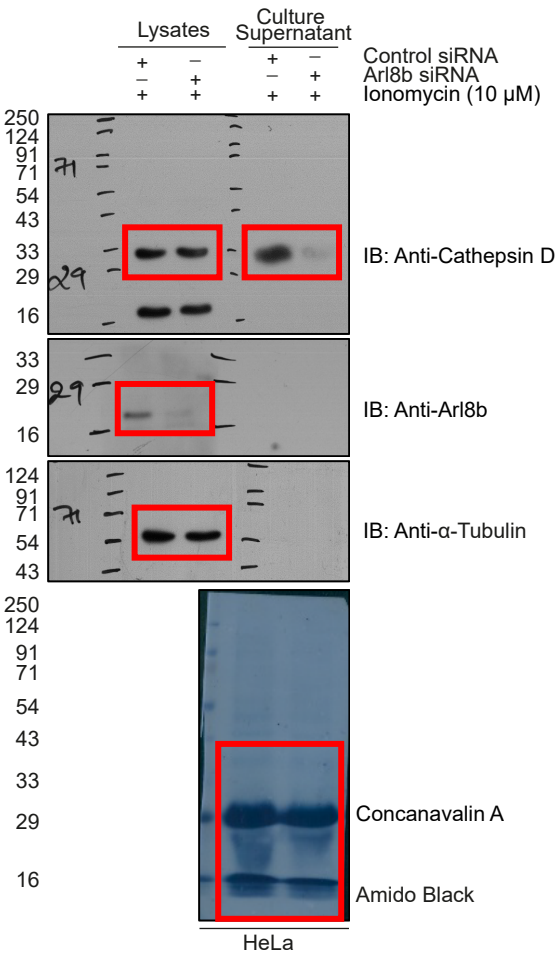

Supplement: SourceData FS1 — is the source file for Fig. S1. [file jcb_202509040_sourcedatafs1.pdf]

Source Data Supplementary Figure 2

Figure S2A

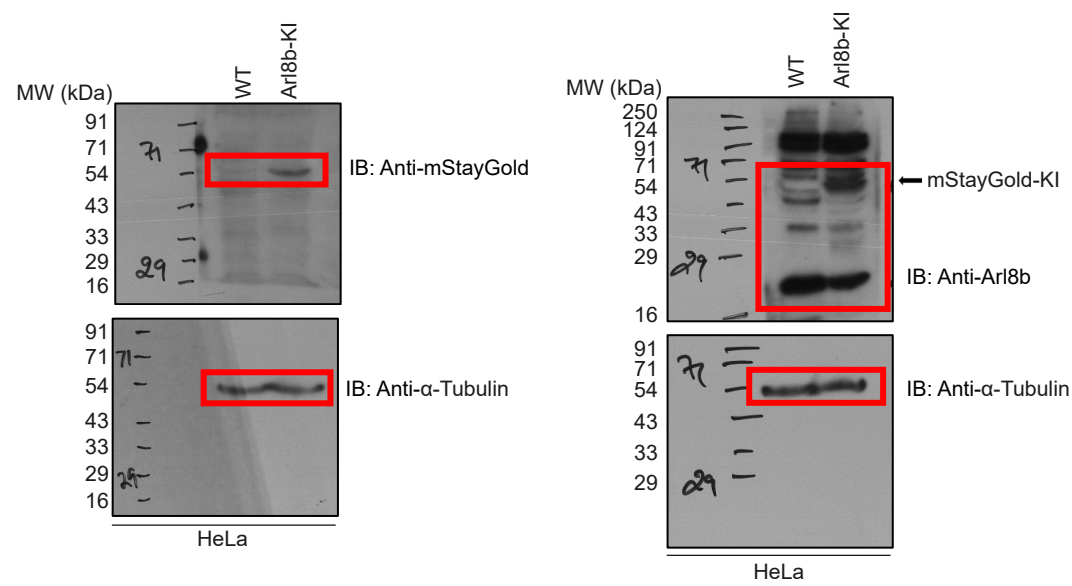

Figure S2H

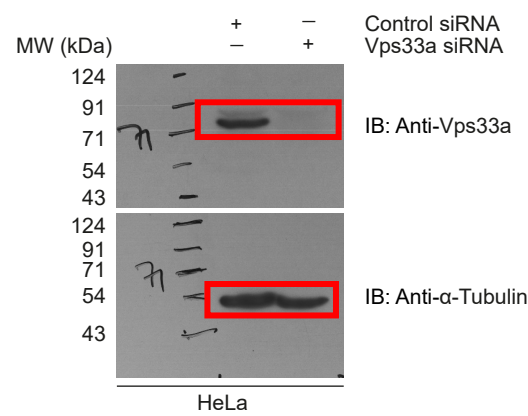

Supplement: SourceData FS2 — is the source file for Fig. S2. [file jcb_202509040_sourcedatafs2.pdf]

Source Data Supplementary Figure 3

Figure S3A

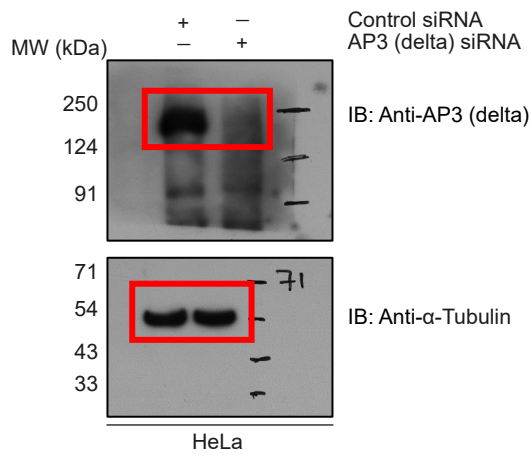

Figure S3M

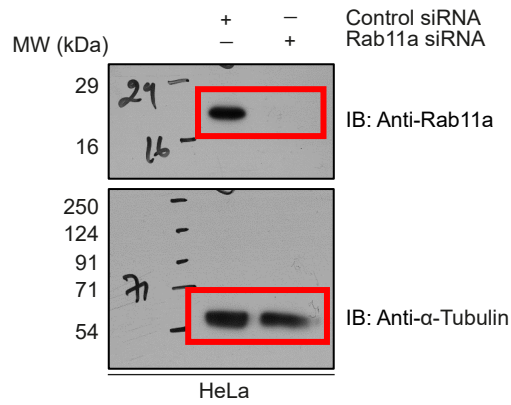

Supplement: SourceData FS3 — is the source file for Fig. S3. [file jcb_202509040_sourcedatafs3.pdf]

Source Data Supplementary Figure 4

Figure S4A

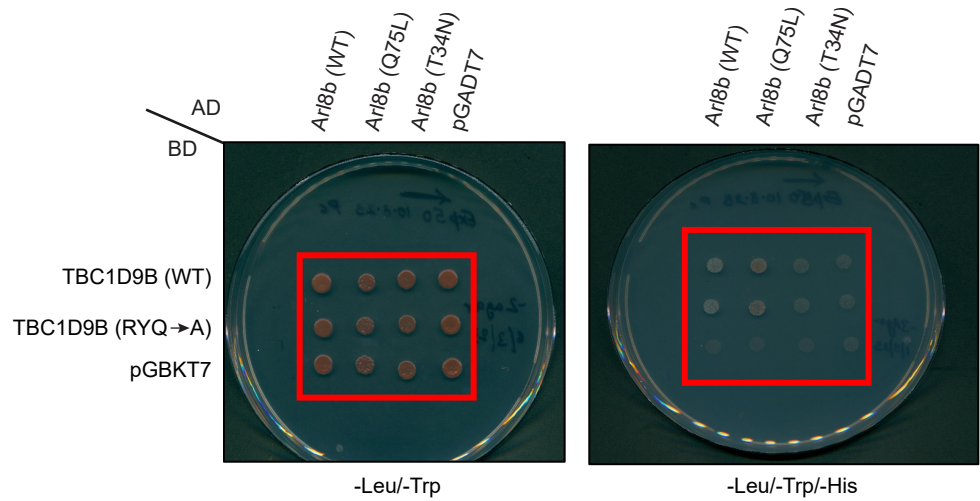

Figure S4D

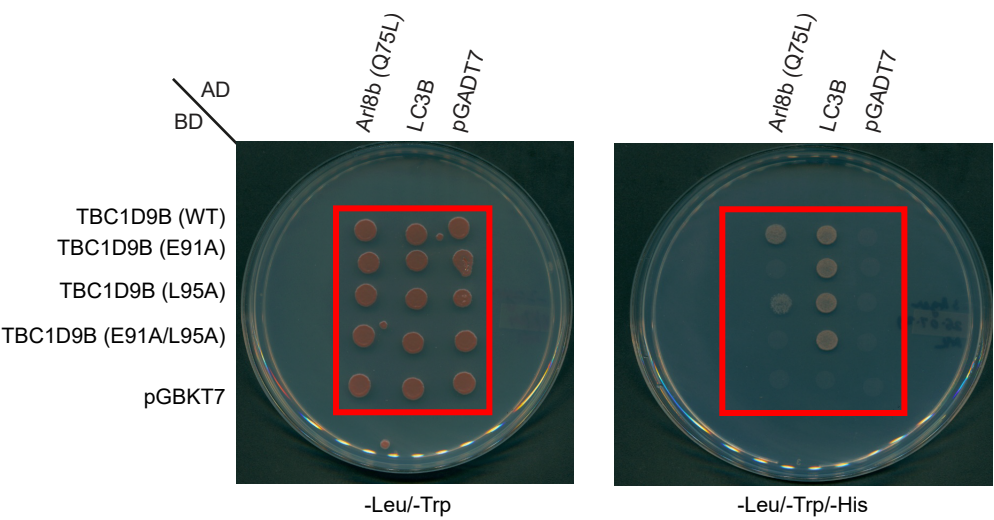

Figure S4G

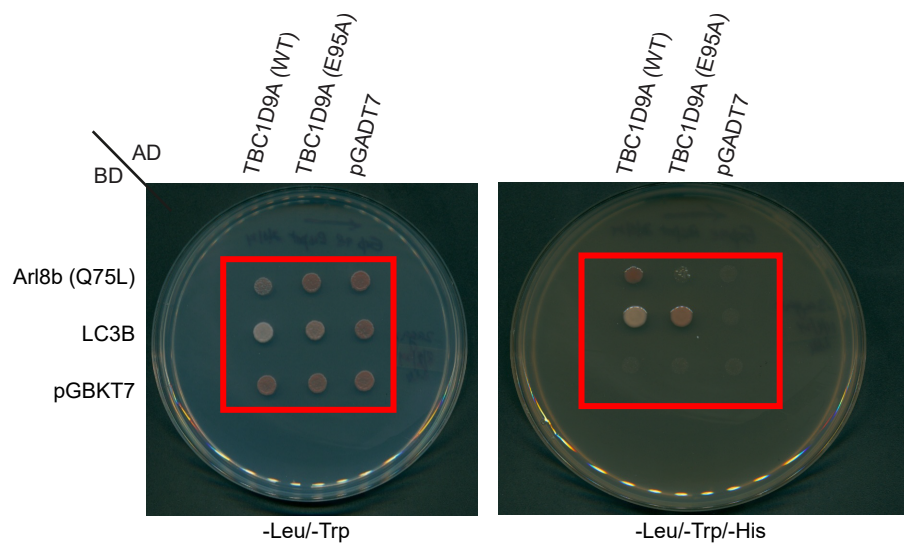

Figure S4H

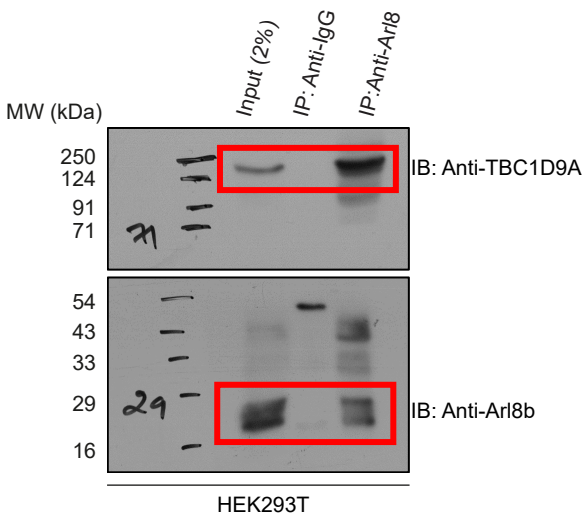

Supplement: SourceData FS4 — is the source file for Fig. S4. [file jcb_202509040_sourcedatafs4.pdf]

Source Data Supplementary Figure 5

Figure S5B

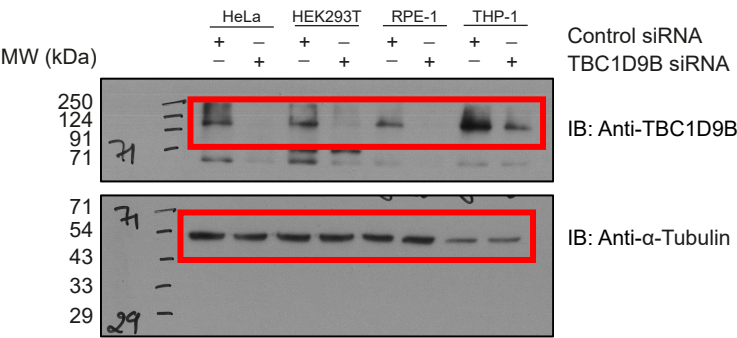

Supplement: SourceData FS5 — is the source file for Fig. S5. [file jcb_202509040_sourcedatafs5.pdf]
